# Cell facilitation promotes growth and survival under drug pressure in breast cancer

**DOI:** 10.1038/s41467-023-39242-6

**Published:** 2023-06-29

**Authors:** Rena Emond, Jason I. Griffiths, Vince Kornél Grolmusz, Aritro Nath, Jinfeng Chen, Eric F. Medina, Rachel S. Sousa, Timothy Synold, Frederick R. Adler, Andrea H. Bild

**Affiliations:** 1 Department of Medical Oncology and Therapeutics Research, Beckman Research Institute, City of Hope National Medical Center, Monrovia, CA 91016 USA; 2 Department of Mathematics, University of Utah, Salt Lake City, UT 84112 USA; 3 School of Biological Sciences, University of Utah, Salt Lake City, UT 84112 USA; 4 Present Address: Department of Mathematical, Computational, and Systems Biology, University of California, Irvine, CA 92697 USA

**Keywords:** Breast cancer, Population dynamics, Evolutionary ecology, Tumour heterogeneity, Computational models

## Abstract

The interplay of positive and negative interactions between drug-sensitive and resistant cells influences the effectiveness of treatment in heterogeneous cancer cell populations. Here, we study interactions between estrogen receptor-positive breast cancer cell lineages that are sensitive and resistant to ribociclib-induced cyclin-dependent kinase 4 and 6 (CDK4/6) inhibition. In mono- and coculture, we find that sensitive cells grow and compete more effectively in the absence of treatment. During treatment with ribociclib, sensitive cells survive and proliferate better when grown together with resistant cells than when grown in monoculture, termed facilitation in ecology. Molecular, protein, and genomic analyses show that resistant cells increase metabolism and production of estradiol, a highly active estrogen metabolite, and increase estrogen signaling in sensitive cells to promote facilitation in coculture. Adding estradiol in monoculture provides sensitive cells with increased resistance to therapy and cancels facilitation in coculture. Under partial inhibition of estrogen signaling through low-dose endocrine therapy, estradiol supplied by resistant cells facilitates sensitive cell growth. However, a more complete blockade of estrogen signaling, through higher-dose endocrine therapy, diminished the facilitative growth of sensitive cells. Mathematical modeling quantifies the strength of competition and facilitation during CDK4/6 inhibition and predicts that blocking facilitation has the potential to control both resistant and sensitive cancer cell populations and inhibit the emergence of a refractory population during cell cycle therapy.

## Abstract (web summary)

In cancer, interactions between treatment-sensitive and resistant cells can influence the effectiveness of therapies. Here, the authors use experimental and mathematical models to explore interactions between ER+ breast cancer cell lineages that are sensitive or resistant to CDK4/6 inhibition, revealing the role of facilitative growth.

## Introduction

Breast cancer is the most common cancer worldwide and the second leading cause of cancer death in American women. The majority (~80%) of these breast tumors are estrogen receptor-positive (ER+), and the majority of metastatic patients who die from their cancer have this breast cancer subtype^1–3^. In these tumors, estrogen receptor activity leads to cancer cell proliferation through cyclin-dependent kinase 4 and 6 (CDK4/6) activation and cell cycle progression^4–6^. In order to target both upstream ER and downstream CDK4/6 signaling for cancer control, the combination of CDK inhibitors with endocrine therapy has been used successfully in metastatic ER+ breast cancer, and to a moderate extent in earlier-stage, non-metastatic breast cancer^7–12^. However, tumors can develop resistance to both single and combination endocrine and cell cycle therapy regimens^7,8,10–15^. Understanding the underlying causes of resistance to endocrine and cell cycle therapies is a critical area of research for this major cancer subtype and cause of death in women.

Cancerous tumors consist of genetically and phenotypically heterogeneous cells^16^. Despite advancements in understanding tumor heterogeneity, this feature is not used to design cancer treatment strategies. ER+ breast cancer is often polyclonal and phenotypically heterogeneous, with co-existing cells of different levels of estrogen receptor expression and different levels of estrogen addiction^17^. Heterogeneous tumors create three major obstacles to treatment. First, cells can have different susceptibilities to treatment, meaning that even a targeted treatment with high efficacy can fail to kill or inhibit a subset of cancer cells^18,19^. Second, this differential survival and proliferation can promote the continued evolution of tumor resistance during drug therapy^16,20^. Third, heterogeneous cell populations create the possibility for interactions among cells that can alter responses to treatment and patient outcomes^21–27^. In this case, understanding how subpopulations communicate could help design therapy strategies that disrupt and/or exploit these interactions for clinical benefit.

This study explores the dynamics of cell interactions, including cooperation and competition, in ER+ breast cancer. Cooperation takes multiple forms and seldom exists in isolation from competition^28,29^. We investigate **facilitation**, the widely used ecological term describing cooperative cases where competitors of one type benefit those of another under appropriate conditions^30^. For example, facilitation emerges among plants competing for water when one plant gains from water that leaks from the roots of a deeper-rooted neighbor^31^. This coincidence of competitive and facilitative interactions requires detailed mathematical models to disentangle their concurrent effects and experiments to identify mechanisms.

To investigate the type, strength, and mechanism of cell interactions in heterogeneous cancer populations during treatment, we use ER+ breast cancer cell lineages that are sensitive or resistant to a CDK4/6 cell cycle inhibitor (ribociclib), a standard therapy used to treat this cancer. Using mathematical models of population growth and interaction, applied to data from mono- and coculture spheroids with mixtures of sensitive and resistant lineages under diverse treatments, we find that ribociclib-resistant cells facilitate sensitive cancer cell growth during cell cycle treatment. Experimental analysis using liquid chromatography-tandem mass spectrometry (LC-MS/MS) assays show that resistant cells secrete excess estradiol. Western blot analysis in resistant cells detects higher levels of aromatase and HSD17β1, key enzymes in the estrogen biosynthesis pathway, contributing to higher production of estradiol, which, given the sensitive cells’ high dependence on estrogen for proliferation, leads to their growth promotion in coculture. Single-cell RNA-sequencing (scRNAseq) analysis reveals that during coculture, sensitive cells acquire the traits of resistant cells, activate oncogenic pathways that drive treatment resistance, and increase proliferation levels. Examining mono- and coculture growth trajectories across a broader range of drug doses, we parameterize a mechanistic model of facilitation. This consumer-resource model reveals that facilitation impairs cell cycle inhibition therapy during the rapid cancer population growth phase and predicts that blocking facilitation can jointly control resistant and sensitive populations. These studies uncover a mechanism of resistance in which the level of local estradiol, the active estrogen metabolite, is increased through production by a subset of cancer cells, leading to cooperative survival and growth of normally therapy-sensitive cells. By understanding cooperative interactions like facilitation, we have the potential to better control co-existing populations within a heterogeneous tumor and inhibit the emergence of resistant phenotypes.

## Results

### Sensitive and resistant cell growth and treatment effects

**Fig. 1:**
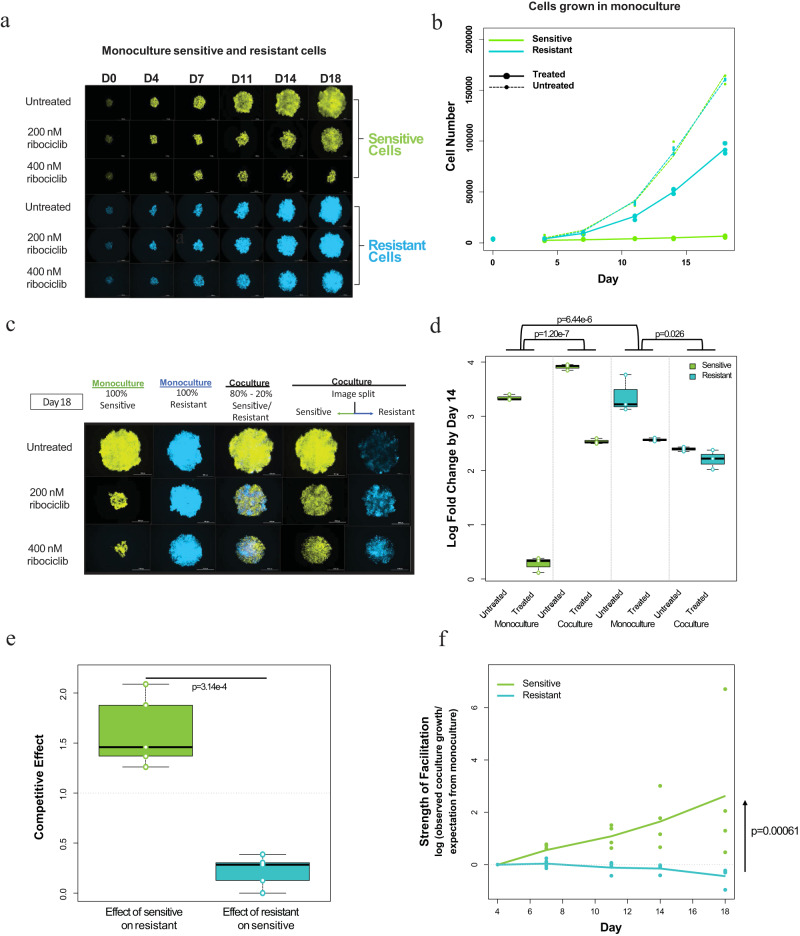
Sensitive and resistant cell growth, treatment effects, and ribociclib-induced facilitation. **a** CAMA-1 spheroids of 100% monoculture sensitive (venus, green) or resistant cells (cerulean, blue) cultured in untreated control, 200 nM ribociclib, and 400 nM ribociclib-treated medium for 18 days. Images taken on Day 18. **b** Growth curves of CAMA-1 untreated and ribociclib-treated (400 nM) sensitive and resistant cell populations. **c** CAMA-1 spheroids of different cell compositions (100% sensitive—green, 50% sensitive–50% resistant, 100% resistant—blue) cultured in untreated, 200 nM ribociclib, and 400 nM ribociclib-treated medium for 18 days. Images taken on Day 18. **d** Box plot of CAMA-1 log fold change of untreated and ribociclib-treated (400 nM) sensitive or resistant cells from day 0 to day 14 in monoculture and coculture. Box plots with centerline = median, box = 25th–75th percentile, and whiskers = 5th–95th percentile, outliers = open circles, *n* = 3 for each case, tests are two-tailed linear models. In monoculture, sensitive cells have a significantly higher reduction in growth than resistant cells (*p* = 6.44e-6 estimated interaction term between cell type and treatment, coefficient = −0.00565, SE = 0.000544, t = 10.376). Sensitive cells have a significantly lower growth reduction in coculture than in monoculture (*p* = 1.20e-7 estimated interaction term between composition and treatment, coefficient = −0.00422, SE = 0.000242, t = 17.423). Resistant cells show a more weakly significant growth reduction in coculture than in monoculture (*p* = 0.026 estimated interaction term between composition and treatment, coefficient = −0.00154, SE = 0.000565, t = 2.722). **e** Box plots of the competitive effect of sensitive (S) cells on resistant (R) cells and vice versa for CAMA-1 cells (*p* = 3.14e-4 with a two-tailed *t*-test, t = −8.0377, the difference in means is 1.391 with 95% confidence interval (−1.824973,−0.957466), *n* = 5). Box plots with centerline = median, box = 25th–75th percentile, and whiskers = 5th–95th percentile, outliers = open circles, *n* = 3 for each case, tests are two-tailed. **f** Facilitation measured as log observed growth relative to expected growth averaged over all replicates for CAMA-1 cells (*p* = 0.00061 for interaction of cell type with day using a linear model of the log of observed over expected cell number as a function of time, coefficient = 0.2125, SE = 0.0566, t = 3.76). Source data provided in Source Data files fig1ddat.csv and fig1edat.csv.

We developed an in vitro model to study ER+ breast cancer cell interactions under selective drug pressure (see “Methods”). To generate cell lineages, we applied ribociclib to cell cultures for 6-9 months until tolerance to the drug (resistance) was developed compared to the control drug-sensitive parental lineage not exposed to treatment^32^. These resistant and sensitive lineages, derived from CAMA-1 ER+ breast cancer cell lines, were labeled with lentivirus to express a fluorescent protein for monitoring each population’s growth when cocultured, and cell counts were calculated as a measure of spheroid area and fluorescence intensity integrated into fitted growth equations (Supplementary Fig. 1a, b). This procedure was repeated for two other cell lines, MCF7 and LY2 (an MCF7 cell line resistant to antiestrogen) (Supplementary Fig. 1c–f)^33,34^. When grown as 3D spheroids in monoculture, untreated sensitive CAMA-1 cell populations grew more quickly than resistant cells. In contrast, while under both 200 and 400 nM concentrations of ribociclib, resistant cells grew more quickly than sensitive cells (Fig. 1a, b). CAMA-1 sensitive cell proliferation was inhibited at higher ribociclib concentrations (200 and 400 nM) whereas resistant cell proliferation was inhibited much less (Fig. 1c, d). The log fold change of the sensitive cell population between days 4 and 14 is reduced by a factor of 7.8 by 400 nM ribociclib treatment, and that of the resistant cell population by a factor of 1.25. Similarly, LY2 and MCF7 sensitive cell proliferation decreased with an increase in ribociclib treatment compared to resistant lineages, but to a lesser extent in MCF7 (Supplementary Fig. 2a, b, e, f).

### Ribociclib tolerance provided by facilitation of sensitive by resistant cells

To investigate whether cell interactions alter the response to therapy, we compared the growth of monocultured and cocultured sensitive and resistant cells treated with different doses of ribociclib over 18 days. We used Lotka–Volterra competition models^35^ to quantify cell interactions and to predict the joint effect of treatment and coculture if one cell type does not alter the drug tolerance of the other. In particular, we determined the expected effects by estimating the cost of treatment and the cost of competition and predicted the combined effect (null model expectation) by multiplying the reductions in the growth rate and the carrying capacity (see “Methods”). Facilitation is identified by a positive difference between observed growth in treated coculture and the expected combined effect from the null model.

Using the competition model, we find that sensitive cells strongly suppress resistant cells in untreated cocultures, with a competitive effect 50% larger than that of resistant cells on themselves (scaled to 1.0 in these models). In contrast, resistant cells had almost no detectable competitive effect on sensitive cells (Fig. 1e). Facilitation was measured as the log observed growth relative to expected growth. Based on the individual effects of ribociclib and coculture, sensitive cells are expected to grow slightly more slowly in ribociclib-treated coculture than in treated monoculture due to competition. Instead, we observed markedly increased growth (Fig. 1c) showing facilitation of sensitive cells by resistant cells (facilitation under 200 nM ribociclib = 0.973; facilitation under 400 nM ribociclib = 2.39) (Fig. 1f). Reciprocally, resistant cells grew more slowly than expected in treated coculture due to the increased suppression by the sensitive cells they facilitate (facilitation under 200 nM ribociclib = −0.055; facilitation under 400 nM ribociclib = −0.227). Despite differences in competitive interactions, facilitation of sensitive cells was also observed in the additional ER+ cell lines, MCF7 (facilitation under 2.4µM ribociclib = 0.239; facilitation under 5µM = 0.4) and LY2 (facilitation under 3µM = 0.659; facilitation under 5µM = 0.479), with LY2 more closely resembling CAMA-1 cell growth and facilitation (Supplementary Fig. 2c, d, g, h).

### Mechanisms of facilitation under ribociclib treatment

To determine the mechanisms driving facilitation during ribociclib treatment, we tested whether resistant cells metabolize ribociclib more effectively than sensitive cells, reducing the ribociclib concentration and allowing an increased proliferation of cocultured sensitive cells. Using an optimized HPLC/MS method, we found that the ribociclib concentration was not decreased more in media incubated with resistant spheroids than with sensitive spheroids, and that ribociclib remained at high doses (Supplementary Fig. 3).

**Fig. 2:**
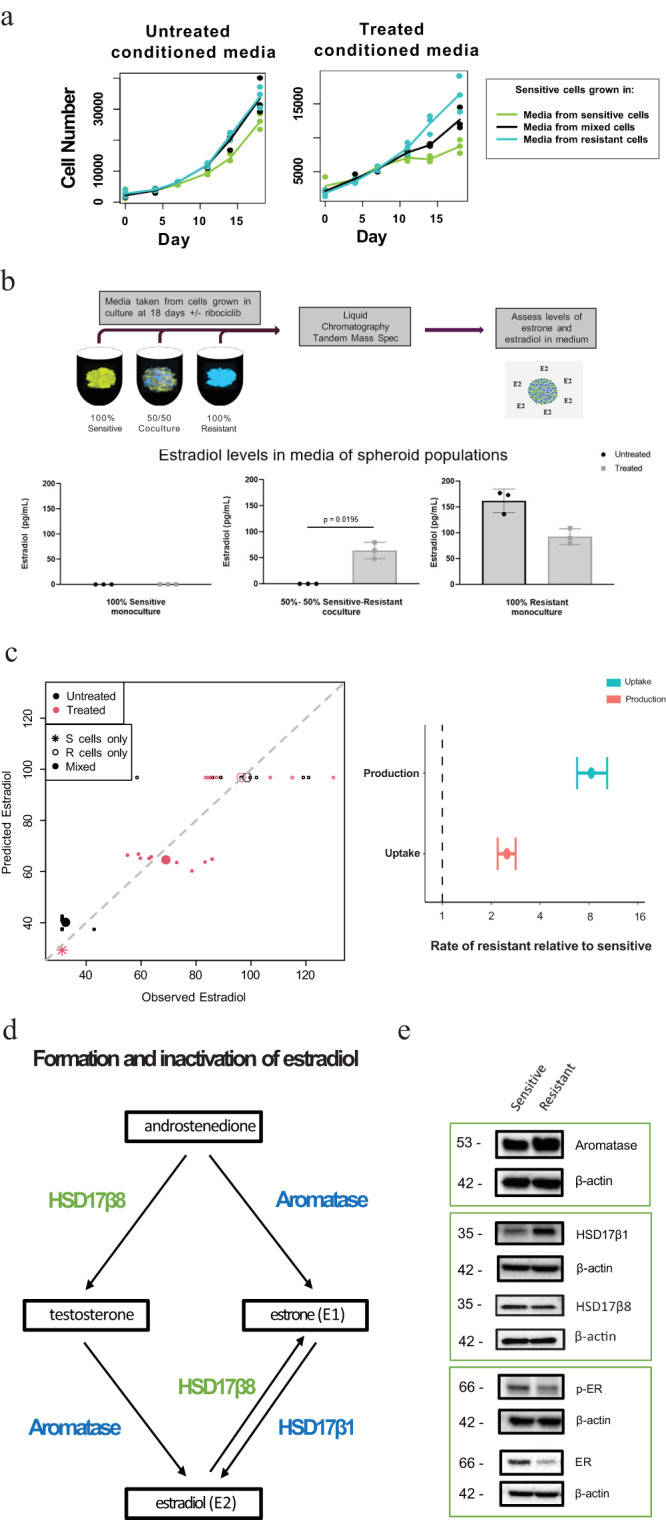
Underlying biological processes driving facilitation in response to ribociclib treatment. **a** Growth of CAMA-1 spheroids under drug pressure with supplemented conditioned media from spheroids of different compositions. Sensitive and resistant cells were plated by the indicated composition of sensitive and resistant cells in a 3D spheroid format and underwent exposure to untreated or 400 nM treated medium to produce conditioned media. 100% sensitive spheroids under drug pressure were then supplemented with different concentrations of conditioned media. Normalized cell counts of sensitive cells supplemented with untreated or treated conditioned media generated from different spheroid compositions. **b** Liquid chromatography-tandem mass spectrometry (LC-MS/MS) assay for estradiol detection performed on CAMA-1 untreated or ribociclib 200 nM treated samples of the following compositions at Day 21: 100% sensitive, 50% sensitive–50% resistant, 100% resistant samples. Box plots are plotted as mean with SD, the boxes range from the 25th to 75th percentile, the whiskers range from the min to max value. Sample size is *n* = 3 for each case. Comparison of estradiol concentration differences between untreated and treated conditions in 50%–50% coculture media (*p* = 0.0195 with a two-tailed *t*-test (t = 7.061). **c** Michaelis–Menten model of estradiol production and uptake fitted to estradiol concentrations in sensitive (S cells: stars) or resistant (R cells: open circles) monocultures and cocultures (Mixed: filled circles) when treated with (red) or without (black) ribociclib. Large points indicate means within treatment groups, small points = replicate measurements. Comparing observed versus predicted estradiol concentrations (left panel) shows the model captured differential estradiol fluxes of sensitive and resistant monocultures and cocultures. Variance explained = 85%, fit line slope = 0.999, adjusted ${R}^{2}$ = 0.85). Comparing estimated estradiol production and uptake of resistant and sensitive cells (right panel points) indicates that resistant cells produce ~8 times more estradiol and use only 2.5 times more than sensitive. Parameter uncertainty is indicated by 95% confidence intervals (CI = error bars). **d** Diagram depicting CAMA-1 resistant and sensitive cell contributions of estradiol metabolism enzymes Aromatase, HSD17β1, and HSD17β8. **e** Western blot analysis of Aromatase, HSD17β1, HSD17β8, phosphorylated-ER, total ER, and β-actin (control) in monoculture, untreated sensitive and resistant CAMA-1 cells. Western Blots were performed in triplicates with consistent findings. Source data provided in Source Data file.

Alternatively, ribociclib-resistant cells may release signaling molecules that enhance the growth of sensitive cells under drug treatment. To test this hypothesis, we compared the proliferation of sensitive cells when supplemented with conditioned media transferred from spheroids of different compositions (100% sensitive, 50%–50% sensitive/resistant, 100% resistant) with or without treatment (0 or 400 nM ribociclib). Conditioned media originating from spheroids containing resistant cells increased the proliferation of sensitive cells significantly more than conditioned media produced by sensitive cell spheroids, with the largest benefit from cells that were themselves treated (Fig. 2a). This indicates that resistant cells secrete signaling molecules that provide pro-growth benefits for sensitive cells under drug pressure. Comparisons of sensitive cell proliferation identified no effect of exosomal transfer on sensitive cell growth under treatment (Supplementary Fig. 4).

To identify the molecules secreted by resistant cells that may promote growth under drug pressure, we measured the concentrations of a broad range of growth-promoting factors in the media of cultured spheroids. Custom multiplex cytokine analysis for detection of known growth factors (TGFβ 1–3, TGFα, EGF, and FGF2, FGF21, FGF23) showed no significant increases in levels in cocultured sensitive and resistant cells compared to sensitive monoculture cells (Supplementary Fig. 5a, b). However, TGFβ3 was found to be higher in the media of monoculture-resistant cells under treatment compared to no treatment, which may be worth future investigation into alternative resistance mechanisms. Interestingly, the concentration of estradiol, a potent estrogen compared to estrone, significantly differed between resistant and sensitive cell spheroids. We used a liquid chromatography-tandem mass spectrometry (LC-MS/MS) assay to detect estrone (Supplementary Fig. 5c) and estradiol concentration under no treatment or 200 nM ribociclib treatment (Fig. 2b; Supplementary Fig. 5c). Estradiol was not detectable in media from 100% sensitive cell cultures in either treatment; in contrast, samples from cultures containing resistant cells showed measurable levels of 64 pg/mL when treated with 200 nM ribociclib. Estradiol production and uptake by resistant and sensitive cells were estimated by fitting a Michaelis–Menten model^35^ and accounting for limits of detection (Fig. 2c, left panel). Resistant cells are estimated to produce approximately 8 times more estradiol and use only 2.5 times more than sensitive cells (Fig. 2c, right panel), generating a substantial source of the growth-promoting hormone.

Given the higher estimation of estradiol produced by resistant cells, we investigated the estrogen biosynthesis pathway and possible differences in the levels of enzymes involved in estradiol metabolism between sensitive and resistant cells (Fig. 2d). Western blot analysis detected higher aromatase levels in resistant cells compared to sensitive cells (Fig. 2e; Supplementary Fig. 6). In looking at a subset of the core estradiol metabolism conversion enzymes, namely HSD17β1 (involved in the conversion of estrone to estradiol, the more active estrogen metabolite) and HSD17β8 (an oxidative enzyme responsible for inactivating estradiol), Western blot analysis indicated higher detectable levels of HSD17β1 in CAMA-1 resistant cells and slightly higher levels of HSD17β8 in sensitive cells (Fig. 2e). Similarly, levels of HSD17β1 were found to be increased in resistant LY2 and MCF7 cells while HSD17β8 levels were found to be increased in sensitive LY2 and MCF7 cells (Supplementary Fig. 6). Furthermore, higher levels of phosphorylated ER and total protein ER levels were identified in sensitive cells compared to resistant cells (Fig. 2e; Supplementary Fig. 6) suggesting a greater activity of and dependence on estrogen signaling in sensitive cells. Conversely, resistant cells displayed lower levels of activated and total ER which supports the notion that localized estradiol is not as necessary or utilized to the same degree as it is in sensitive cells and that resistant cells may rely on other growth factor signaling pathways for proliferation independent of estradiol. Together, these results suggest that increased levels of estradiol may be driven through multiple mechanisms involving aromatase and conversion enzymes of the HSD17β family (Fig. 2d). With an excess of localized estradiol synthesized and supplied by resistant cells, estrogen-dependent sensitive cells can then be facilitated in growth by resistant cells when cocultured under drug treatment. While LY2, like CAMA-1, exhibited higher levels of HSD17β1 in resistant cells (Supplementary Fig. 6a), a smaller distinction was found between sensitive and resistant MCF7 cells (Supplementary Fig. 6b), consistent with the lower facilitation in MCF7 cells than in LY2 and CAMA-1. Experiments in media deprived of androgens from charcoal stripped fetal bovine serum produced total growth inhibition in both resistant and sensitive cells (Supplementary Fig. 7). These analyses support a model of facilitation whereby ribociclib-resistant cells produce excess estradiol via an androgen synthesis pathway with increased aromatase and HSD17β1, countering sensitive cells’ inactivation of estradiol via HSD17β8, and ultimately promoting sensitive cell growth during treatment through increased estrogen signaling (Fig. 2d, e).

### Modulation of estrogen signaling attenuates facilitation

**Fig. 3:**
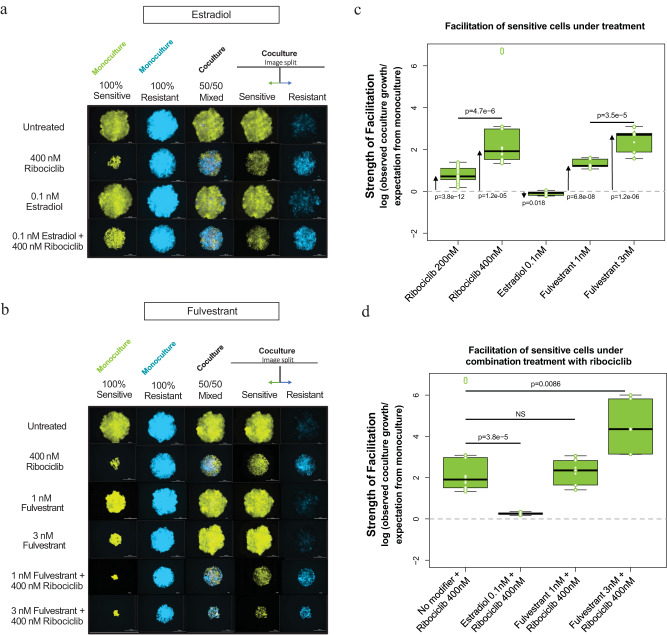
Modifying endocrine signaling pathways affects spheroid growth and facilitation. **a** CAMA-1 spheroids of different cell compositions (100% sensitive—green, 50% sensitive–50% resistant, 100% resistant—blue) cultured in untreated, 400 nM ribociclib, 0.1 nM estradiol, or combination 400 nM ribociclib with 0.1 nM estradiol-treated medium for 18 days; images taken on day 18. **b** CAMA-1 spheroids of different cell compositions (100% sensitive—green, 50% sensitive–50% resistant, 100% resistant—blue) cultured in untreated, 400 nM ribociclib, 1 nM or 3 nM fulvestrant, or combination 400 nM ribociclib with 1 nM or 3 nM fulvestrant treated medium for 18 days; images taken on day 18. **c** Facilitation of sensitive cells across monotherapy treatments (over days 11, 14, and 18). Box plots with centerline = median, box = 25th–75th percentile, and whiskers = 5th–95th percentile. For each treatment, two-tailed *t*-tests assessed the deviation (arrows) of observed growth in coculture from the expected combined effect of treatment and coculturing produced by the null model (dashed line: zero facilitation) (sample size = 27 for 200 nM ribociclib, 18 for 400 nM ribociclib, 9 for other treatments). All differences from zero facilitation are significant (ribociclib 200 nM, *p* = 3.8e-12, t = 12.05, mean = 0.783, 95% CI = 0.649:0.917; ribociclib 400 nM, *p* = 1.2e-05, t = 6.09, mean = 2.736, 95% CI = 1.788:3.684; Estradiol 0.1 nM, *p* = 0.0186, t = 2.95, mean = −0.010, 95% CI = −0.179:−0.022; fulvestrant 1 nM, *p* = 6.8e-08, t = 18.75, mean = 1.307, 95% CI = 1.146:1.468; fulvestrant 3 nM, *p* = 1.2e-06, t = 13.01, mean = 2.443, 95% CI = 2.010:2.876). Facilitation increased with dose of ribociclib (linear model, *p* = 4.7e-06, coefficient = 0.0098, SE = 0.00187, t = 5.23) and fulvestrant (*p* = 3.5e-5, coefficient = 0.568, SE = 0.100, t = 5.67). **d** Facilitation of sensitive cells under combination treatments across days 11, 14, and 18. Box plots with centerline = median, box = 25th–75th percentile, and whiskers = 5th–95th percentile. Two-sided ANOVA compared facilitation measured under ribociclib monotherapy and under estrogen-modulating combination treatments. Combination with 0.1 nM estradiol significantly reduced facilitation (*p* = 3.8e-05, t = 5.51, estimate = 2.478, 95% CI = 1.530:3.427), although some facilitation remained (*T*-test of deviation from zero facilitation (dashed line): *p* = 6.319e-07, t = 14.07, mean = 0.258, 95% CI = 0.215:0.300). Facilitation was unchanged by combination with low-dose fulvestrant (1 nM) but increased by combination with higher-dose fulvestrant (3 nM) (*p* = 0.0086, t = 2.866, estimate = 1.720, 95% CI = 0.480:2.960). Sample size = 18 for 400 nM ribociclib and 9 for other treatments. Source data provided in Source Data files fig3cdat.csv and fig3ddat.csv.

Applying ribociclib treatment to cocultures revealed the facilitation of sensitive cells by resistant cells. Furthermore, the degree of facilitation increased with ribociclib in a dose-dependent manner (Fig. 3). Our experiments indicate that resistant cells facilitate sensitive cells by increasing the level of local estradiol. To test this mechanism and assess disruption of facilitation, we added exogenous estrogen pathway modifiers to cells in monoculture and coculture, both with and without ribociclib treatment. Estrogen pathway modifiers included supplemented estradiol (Fig. 3a) and a panel of endocrine therapies with differing mechanisms of action (Fig. 3b; Supplementary Fig 8; Supplementary Fig. 9). SERMs (selective estrogen receptor modulators, like tamoxifen and raloxifene) bind to ER, resulting in an inactive complex, while SERDs (selective estrogen receptor degraders, like fulvestrant) disrupt signaling by preventing dimerization and targeting ER for degradation. Aromatase inhibitors (such as letrozole and exemestane) block the production of estradiol and can be reversible (temporary enzyme inhibition) or irreversible (promoting enzyme destruction). We used a range of concentrations in 2D and 3D cultures to gauge the cell lines’ dose response to drugs of each class. For simplicity, effective concentrations (but not overly detrimental to cell viability) were chosen for representation. We then measured the strength of facilitation of sensitive cells with and without the addition of estrogen pathway modifiers to ribociclib treatment (Fig. 3c, d; Supplementary Fig. 9b, d, f, h) again by quantifying the log of observed growth of cells relative to expected with a null model that assumes that the effects of treatments on growth are independent and multiplicative.

We tested two specific predictions:

1. Facilitation of sensitive cells will not be observed in cocultures treated with supplementary estradiol, as further provision by resistant cells is surplus.
  Estradiol monotherapy improved the growth of sensitive cells in monoculture, consistent with sensitive cells being dependent on estradiol signaling. In estradiol-treated cocultures, the observed growth of sensitive cells was slightly lower than expected if the effects of competition with resistant cells and estradiol treatment were independent, indicating cancellation of facilitation (Fig. 3a, c; Supplementary Fig. 8b, c). This result supports the hypothesis that estradiol facilitates sensitive cell growth. Further, without ribociclib selective pressure, competition from sensitive cells blocked resistant cell growth and the facilitation of sensitive cells.
  Under combination therapy, the addition of estradiol with ribociclib also canceled the facilitation of sensitive cells by resistant cells observed when treated with ribociclib alone (Fig. 3a, d, Supplementary Fig. 8c, e). We saw no dose-dependent response, likely due to the saturation of the estradiol-binding capacity of sensitive cells, as local estradiol from resistant cells will then be redundant.
  Overall, the results indicate that complete estradiol deprivation halts sensitive cell proliferation (Supplementary Fig. 7) and that excess estradiol supplementation cancels sensitive cell facilitation due to a saturation of estradiol binding capacity.
2. Facilitation of sensitive cells will be observed in cocultures treated with partial estrogen signal-blocking endocrine therapies as estradiol from resistant cells maintains ER pathway activity. However if this pathway is more completely inhibited, we expect cancellation of facilitation.

We observed that some endocrine therapies produce partial blockage of estrogen signaling and resistant cell growth in our system (perhaps due to cross-resistance mechanisms), including fulvestrant (Fig. 3b–d; Supplementary Fig. 8d) and letrozole (Supplementary Fig. 9a, b). Under these therapies, resistant cells facilitated sensitive cell growth in coculture compared to monoculture. This result supports the hypothesis that without effectively disrupting the ER pathway, the facilitation of sensitive by resistant cells will persist. In contrast, endocrine therapies that more completely block estrogen signaling and proliferation of resistant cells in our system, such as exemestane (Supplementary Fig. 9c, d), tamoxifen (Supplementary Fig. 9e, f), and raloxifene (Supplementary Fig. 9g, h), effectively inhibited facilitation.

While each drug class inhibits ER signaling, the differing degree of facilitation under treatment with an AI, SERM, or SERD reflects their differing abilities to block resistant cell provision of estradiol to stimulate sensitive cell receptors. In our in vitro system, cells resistant to ribociclib are also cross-resistant to the SERD, fulvestrant. However, SERMs are more damaging to resistant cell growth while also preventing estradiol from binding to the estrogen receptors of sensitive cells which leads to the cancellation of facilitation. Cells resistant to ribociclib are also more resistant to the AI, letrozole, compared to exemestane, a more potent and non-reversible steroidal AI. Similarly, exemestane is also effective at inhibiting resistant cell growth, disrupting estrogen production, and blocking facilitation.

Together, these results show that resistant cells can facilitate sensitive cell growth under partial blockage of ER signaling by providing supplementary estradiol signals. However, endocrine therapies that more completely block estradiol production or receipt of estradiol by sensitive cells block facilitation. The degree to which facilitation may be blocked is dependent on the resistant cells’ level of sensitivity to each modifier and, as a result, to what extent estradiol production or estrogen signaling is disrupted.

### Coculture results in a shift of sensitive cells to a more resistant cell state as revealed by scRNAseq

**Fig. 4:**
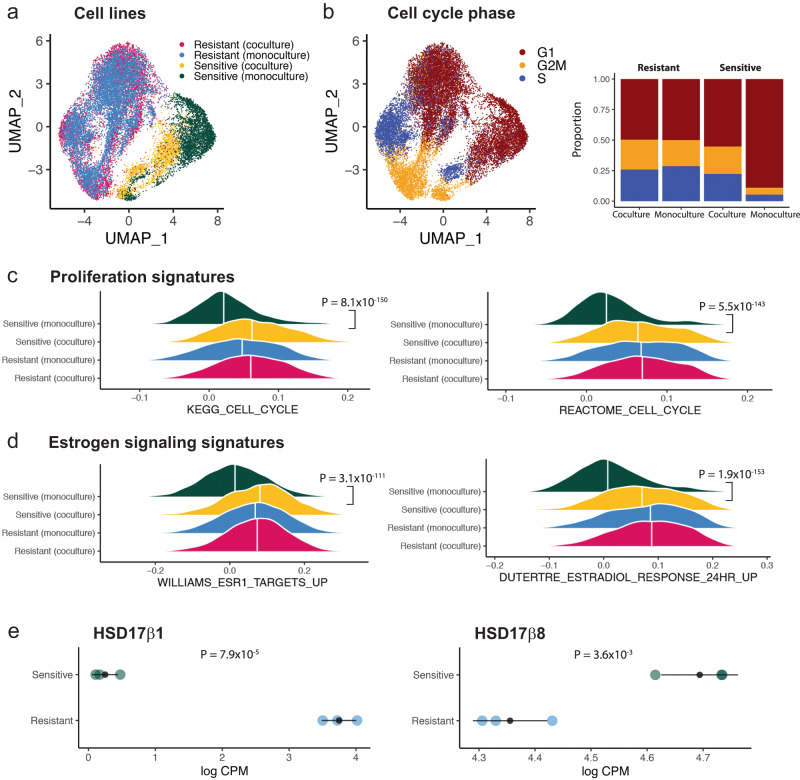
RNA sequencing reveals transcriptomic differences between mono- and cocultured sensitive and resistant cells. **a** UMAP of scRNAseq profiles from 6292 resistant (coculture), 7557 resistant (monoculture), 2059 sensitive (coculture) and 3329 sensitive (monoculture) CAMA-1 cells. **b** Cell phenotypic heterogeneity visualized using UMAP and colored according to cell phase estimated using the scRNAseq profiles of CAMA-1 cells. Stacked bar plots on the right show proportion of cells within each phase of the cell cycle. **c** Ridge density plots showing the density of ssGSEA enrichment scores for cell cycle pathways across different cell lines. The white vertical line indicates the median of the distribution, with FDR adjusted *p*-value from the non-parametric (two-sided) Wilcoxon rank-sum test between sensitive monoculture vs. sensitive coculture indicated to the right. **d** Ridge density plots showing the density of ssGSEA enrichment scores for estrogen signaling response pathways across different cell lines. The white vertical line indicates the median of the distribution, with FDR adjusted *p*-value from the non-parametric (two-sided) Wilcoxon rank-sum test between sensitive monoculture vs. sensitive coculture indicated to the right. **e** Dot plots showing gene expression levels measured in ribociclib-resistant and sensitive cells using bulk RNAseq. Each colored dot indicates one independent biological replicate (*n* = 3 for sensitive and *n* = 3 for resistant cells), with the gray dot and bars indicating mean and standard deviations. The *p*-values indicate the significance of the difference in means from the two-sided Welch’s *t*-test. Source data provided in Source Data file.

We hypothesized that hormone communications from resistant to sensitive cells activate proliferative pathways of sensitive cells when cocultured during ribociclib treatment resulting in a more resistant phenotype. We performed scRNAseq on sensitive and resistant CAMA-1 cells grown in spheroids after 11 days of treatment, both in monoculture and coculture with initially equal proportions. Following quality control filtering and normalization of the scRNAseq profiles (see “Methods”), we performed UMAP dimension reduction to compare the phenotypic similarity of cells across the transcriptome. Monoculture-sensitive and resistant lineages formed separate clusters, indicating that they are phenotypically distinct, with a shift of sensitive cells toward resistant cells seen when grown in coculture (Fig. 4a). We next calculated the cell cycle phase of each cell using canonical markers (Fig. 4b) and found a striking increase in the proportion of cycling sensitive cells in coculture compared to sensitive cells in monoculture. This analysis revealed that resistant cells facilitated the cell cycle progression of sensitive cells under drug pressure.

We next investigated the key pathway phenotypes acquired by the sensitive cells in coculture compared to monoculture using single sample gene set enrichment analysis. We found a highly significant increase in the KEGG and REACTOME cell cycle pathway enrichment scores (both *p* < 0.00001), supporting the cell cycle phase analysis results (Fig. 4c). Differential expression analysis confirmed that both proliferation and estrogen signaling gene expression was elevated in sensitive cells when cocultured compared to cells in monoculture (Supplementary Fig. 10).

Gene expression signatures indicative of estrogen signaling activation, including ESR1 targets (*p* = 3.1 × 10^−^^111^) and estradiol response (*p* = 1.9 × 10^−^^153^), were also elevated in sensitive coculture cells (Fig. 4d). Finally, differential expression of estradiol production enzymes were detected between resistant and sensitive cells with increased levels of HSD17β1 in resistant cells while HSD17β8 was elevated in sensitive cells (Fig. 4e), with low/non-detectable aromatase expression.

### Measuring the impacts of growth factor-mediated facilitation

**Fig. 5:**
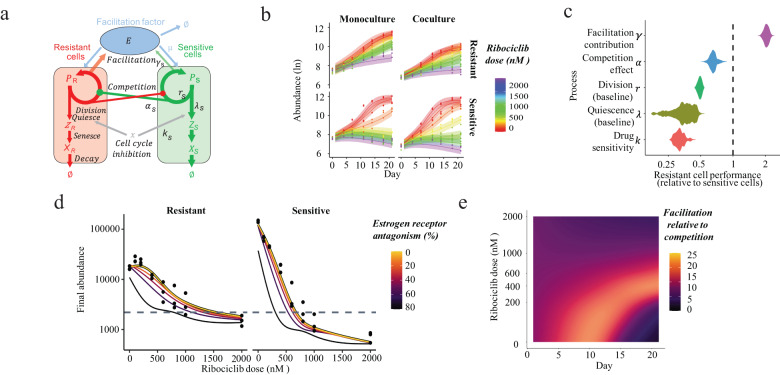
Measuring the strength and role of facilitation during treatment. **a** Structure of mechanistic stage-structured differential equation model of facilitation fitted to data. This describes the abundance of resistant (red) and sensitive (green) cells that compete (circle-headed arrows) and facilitate one another by producing facilitation factors (E; estradiol; blue) used by both cell types to promote division. Cancer cells transition through proliferative (P), quiescent (Z), and then senescent (X) stages. Proliferative cells enter the G1/S cell cycle checkpoint at a rate depending on competitor abundance and facilitation factor concentration. Cells entering the G1/S checkpoint either divide or enter the quiescent state. Cell cycle inhibitor therapy increases the cell fraction entering the quiescent stage. **b** Spheroid growth of sensitive and resistant cells (rows), alone or in coculture (columns) and across ribociclib doses (color). Observed cell counts (points) are predicted by the facilitation model (lines) across doses and cell compositions. Model uncertainty captured by 95% high credibility intervals (shaded regions). **c** Quantification of resistant cells’ abilities, relative to sensitive cells, to facilitate neighboring cell growth, compete for resources, divide or quiesce in the absence of therapy, and their drug sensitivity. Violin plots show ranges of relative performance yielding spheroid trajectories consistent with observations (shaded region = Bayesian posterior parameter distributions; HMC samples = 3000). The vertical dashed line indicates the equal performance of cell types. **d** Predicted impact of blocking facilitation on sensitive and resistant cell growth in coculture, across drug dose. Observed (points) and model predicted (thick black bordered lines) final spheroid size shown across ribociclib doses without facilitation blocking (0% ER antagonism). Thinner lines show the predicted size achievable by reducing facilitation (brighter lines = greater facilitation levels). The horizontal line indicates initial abundance. **e** The role of facilitation, relative to competition, on cocultured sensitive spheroid proliferation over time (*x*-axis) and across drug doses (*y*-axis). Coloration shows the relative contribution of facilitation to sensitive cells G1/S phase entry (${{{{{{\rm{G}}}}}}}_{{{{{{\rm{Sensitive}}}}}}}$) at specific moments of treatment (brighter coloration = greater importance of facilitation). The bright band shows the facilitation window (cell abundance increasing but carrying capacity not reached). Source data provided in Source Data file.

To test whether our proposed facilitation mechanisms can predict the growth of sensitive and resistant cells under a broad range of conditions, we constructed stage-structured consumer-resource models that describe the production and uptake of estradiol, separate cell division and death, and include functional forms for the effects of ribociclib and estradiol (Fig. 5a) (detailed in “Methods” and Supplementary Information). To parameterize these mechanistic models, we performed mono- and coculture experiments across a wider range of ribociclib drug doses (Fig. 5b). The estimated parameters quantify three expected changes in resistant cells: (1) Reduced growth inhibition by ribociclib, (2) Reduced growth and competition effect, (3) Increased production of the facilitation factor estradiol (Fig. 5c). Finally, we use the parameterized models to test whether facilitation-blocking therapy can reduce the growth of cancer cell populations (Fig. 5d) and to identify the phase of spheroid growth and drug doses under which facilitation is most impactful (Fig. 5e).

We used Bayesian inference to identify the biological rates (indicated in Fig. 5a and detailed in “Methods”) under which the model most accurately predicts spheroid growth trajectories of monocultures and cocultures of both sensitive and resistant cells across eight doses of ribociclib. Supporting our hypothesis that facilitation played a key role in promoting spheroid growth, we found that the model of estradiol-mediated facilitation accurately describes the growth of resistant, sensitive, and coculture spheroids across all drug doses (Fig. 5b), explains the frequently observed lag in initial spheroid growth and the delayed shrinkage of spheroids at high drug doses after an initial growth phase.

We verified that models of alternative mechanisms of cell interaction could not produce the diversity of spheroid growth trajectories observed across drug doses and compositions. Alternative models included direct competition for resources and phenotypic plasticity in which cells transition from a naive to a resistant state either in response to drug induction or via random switching (detailed model comparisons provided in Supplementary Information). We performed a formal probabilistic model comparison of the non-nested set of model hypotheses using Watanabe–Akaike information criterion (WAIC). The facilitation model greatly outperformed models of competition or phenotypic plasticity (Supplementary Fig. 11). This analysis guarded against model overfitting and supports the estradiol-mediated facilitation hypothesis.

The fitted model of estradiol-mediated facilitation quantifies how biological processes differed between resistant and sensitive cells (Fig. 5c). Given our hypothesis that sensitive cell growth under ribociclib treatment is promoted by estradiol released by ribociclib-resistant cells, we expected to measure higher estradiol production rates in resistant cells. The net production of estradiol ($\gamma$) by resistant cells was estimated to be approximately twice that of sensitive cells, consistent with the ratio of production/use estimated directly (8.0/2.5 = 3.2, Fig. 2c). Under the hypothesis that resistance is costly, we also expected sensitive cells to be competitively dominant over resistant cells. In agreement, resistant cells were found to be approximately 60% as competitive ($\alpha$), consistent with the 1.5 times higher competitive effect of sensitive cells (Fig. 1e). In further support of the cost of resistance, in the absence of treatment, the less competitive resistant cells had a baseline division rate ($r$) that was 50% of sensitive cells despite a lower quiescence rate ($\lambda$). However, they were far less sensitive to the cell cycle inhibitory effects of ribociclib ($k$), allowing their spheroids to continue to grow at high doses. This indicates that resistant cells evolved a slower-growing, longer-lived cell strategy as well as bypassing the G1-S phase cell cycle blockade of ribociclib.

We use this parameterized model to explore the impact of therapeutically blocking facilitation on the growth of sensitive and resistant cocultured populations. To do so, we reduced the binding of estradiol to its cellular receptor ($\mu$) by differing degrees to reflect exposure to different doses of an ER-blocking drug such as fulvestrant. In the absence of facilitation blocking, the facilitation model accurately predicts the final resistant and sensitive cell population size (Fig. 5d; points show observed final cell counts at day 21, black line + yellow overlay shows model predictions under the experimental conditions). However, facilitation needed to be reduced by more than 50% before substantial reductions in spheroid growth were predicted, suggesting that resistant cells produce sufficiently large amounts of estradiol to saturate the growth benefits of facilitation. At lower ribociclib doses (<500 nM), facilitation blocking caused a more severe reduction in the abundance of sensitive than resistant cells, suggesting that resistance could be promoted under these circumstances. This result shows that facilitation targeting treatments will have dose-dependent consequences on the evolution of resistance that will require a quantitative understanding of underlying molecular mechanisms.

Finally, to investigate our hypothesis that facilitation is a key process promoting sensitive cell proliferation, we used the fitted model to assess the importance of facilitation relative to resource competition in determining sensitive cell proliferation throughout treatment with various ribociclib dosages. To do this, we decomposed the model-inferred rate of G1/S phase entry into the contributions of facilitation and competition processes (Fig. 5e). Across ribociclib doses (<600 nM), facilitation was many times more important than competition during the period after the accumulation of sufficient population size to generate high levels of estradiol but prior to the spheroid reaching maximal density and becoming regulated by resource limitation. At moderate ribociclib doses (200–600 nM), the facilitation window shifts later into the treatment, as resistant cell abundances increase more slowly, and the window also extends for a longer duration. At the highest ribociclib doses (>600 nM), the window of facilitation closes completely because the proliferation of resistant facilitating cells is increasingly controlled (Fig. 5e). Coculture spheroid size then shrinks during treatment, as the small resistant population alone cannot maintain estradiol levels to overcome treatment. This mechanism of facilitation predicts and quantitatively explains the observed initial lag in spheroid growth and delayed spheroid shrinkage, following a growth phase, at high ribociclib doses (Fig. 5b). The closure of the facilitation window at high ribociclib doses is driven by a lack of resistant facilitating cells, not driven by increasing competition (lower cancer cell abundances make more resources available per cell).

Overall, the results support our experimental findings that strong inhibition of ER signaling is needed to overcome the facilitation of sensitive cells. However, blocking facilitation has the potential to control both resistant and sensitive cancer cell growth, inhibiting the emergence of a refractory population during cell cycle therapy.

## Discussion

We developed in vitro model systems to investigate how interactions between cancer lineages impact the growth of heterogeneous ER+ breast cancer populations. We found that cells sensitive to ribociclib (a CDK4/6 cell cycle inhibitor) grow faster in untreated monoculture and outcompete resistant cells in coculture. However, in the presence of ribociclib, resistant cells facilitate the growth of sensitive cells by producing local estradiol, a potent estrogen metabolite, resulting in an upregulation of estrogen signaling and proliferation. Although resistant cells were developed by long-term growth with ribociclib, they also acquired cross-resistance to endocrine therapy and also facilitate the growth of sensitive cell populations in the presence of ER antagonists unless there is a complete inhibition of estrogen signaling at higher doses with effective drugs.

Analysis of scRNAseq data of resistant and sensitive cell populations uncovered some potential mechanisms of cross-resistance to cell cycle and endocrine therapies. We found an increase in the MYC, EGFR, and TGFβ receptor signaling pathway activity in resistant cells (Supplementary Table 1). These pathways have been found to underlie resistance to both endocrine and cell cycle therapies in independent studies^36–42^. Patterns of cross-resistance and cell–cell interactions complicate patient treatment even beyond the challenge of the evolution of resistance.

We use mathematical models to quantify competitive and facilitative interactions and to predict how different conditions will alter facilitation. Mathematical models that include facilitation improve our ability to describe the growth of heterogeneous cancer populations. These models predict that blocking facilitation has the potential to control both resistant and sensitive cell populations and inhibit the emergence of a refractory population, prolonging the benefits of ribociclib. This result may explain why ribociclib is more effective when combined with antiestrogen therapy than as monotherapy for some patients^43–45^.

The dynamical models highlight that the contribution of facilitation to proliferation and cancer population growth shifts during the growth of cancer. As cell abundance increases, facilitation-driven growth diminishes as carrying capacity is reached, due to an increase in resource competition. This shift suggests that competitive ability may be more essential at later stages in disease progression, while facilitation may be more important in earlier stages. Future studies may also integrate local spatial effects in our measurements and modeling to account for any impact on cell–cell interactions.

Mathematical modeling^46–48^ and experimental^49–51^ research proposes that tumor growth and progression may be promoted by cooperation among diverse cell populations by sharing resources or products through mechanisms including neoangiogenesis^46^ and growth factor production^51^. Our results support the concept that cancer cell facilitation may impact heterogeneous tumor growth during treatment in ER+ breast cancer. No current therapy is directed specifically at cooperative cancer cell interactions. However, as tumors are often comprised of multiple subclonal populations, interactions between cells may provide targets for long-term treatment strategies. Given that facilitation can increase both persistence and biomass relative to simpler systems with resource competition only^52,53^, it is likely to be frequent in heterogeneous cancer populations. As we gain capabilities to measure the growth of heterogeneous subclone populations during treatment, we may tailor strategies to control faster-growing yet drug-sensitive cells while preventing the dominance of refractory cells through competition with more fit subclones.

This study describes facilitation through an increase of local estradiol concentration by resistant cells that stimulates the proliferation of sensitive cells during selective pressure. It is known that estrogen biosynthesis and metabolism can be upregulated in cancer cells^54–56^. It remains unknown whether resistant cells in our system acquired mutations to increase estradiol levels or if changes are epigenetic. The increase of active estrogen is important given that the majority of breast cancers are hormone receptor-positive and resistance to hormone therapy is common in late-stage breast cancer patients^57–59^. In patients with increased levels of estradiol, potential treatment strategies include blocking the enzymes that synthesize estradiol in resistant cells or modulating the level or type of endocrine therapy given to a patient. Targeting the diffusible facilitation factor has been proposed to be a more evolutionarily stable treatment strategy than targeting the cancer cell receptors or cell intrinsic signaling pathways^60^, as the production of a public good provides little benefit to the producing cell, weakening the fitness advantage of the resistance trait. This effect lessens the selection pressure for resistance and if sufficiently costly could robustly halt the evolution of this phenotype. An important next step is to study these mechanisms in other settings such as endocrine-resistant specific cell lines and patient tumors, and to better understand the presence and impact of cell cooperation in response to therapy and outcomes.

In conclusion, our data indicate that the facilitation within heterogeneous cancer cell populations shapes the dynamics of resistance and growth of cancer cell populations. We show that local production of a highly active estrogen, estradiol, by resistant cells facilitates the growth of sensitive cells in the presence of drug treatment, and conveys resistance to anti-proliferative effects of cell cycle inhibition in sensitive cells. We use mathematical models to measure these processes and show that blocking this facilitation could promote the response of the entire cancer population, reducing selection for refractory resistant states. This work provides support for the development of strategies to modulate facilitation to create robust and durable cancer control.

## Methods

### Cell lines and reagents

The previously authenticated estrogen receptor-positive (ER+) CAMA-1 breast cancer cell lines (ATCC, Cat # HTB-21) were maintained in DMEM+10% FBS+1% antibiotic–antimycotic solution. The ribociclib-resistant CAMA-1 cell line creation (ribociclib-resistant CAMA-1) was previously reported^32^. Briefly, cells were cultured and continuously treated with ribociclib (Selleck Chemicals, Cat. No: S7440) at 1 µM for 1 month. Following the initial 1 µM ribociclib treatment, cells were treated with 250 nM for 4 months to develop resistance. Maintenance of ribociclib-resistant CAMA-1 cells continued in complete culture medium + 250 nM ribociclib. Resistance against ribociclib was detected by the alteration of the dose–response curve measured using CellTiterGlo Chemiluminescent Kit (Promega Corporation, Cat. No.: G7573).

### Lentiviral labeling of sensitive and resistant cells

Using lentiviruses incorporating distinct fluorescent proteins, we labeled CAMA-1 parental sensitive cells (venus; LeGO-V2) and ribociclib-resistant cells (cerulean; LeGO-Cer2). LeGO-V2 and LeGO-Cer2 vectors were provided by Boris Fehse (Addgene plasmids #27340 and #27338). Lentiviruses with fluorescent proteins were created using Lipofectamine 3000 reagent (Thermo Fisher Scientific) following the manufacturer’s protocol. CAMA-1 sensitive and resistant cell lines were transduced with lentivirus using reverse transduction. Briefly, 1 mL of polybrene-containing cell suspension of 200,000 cells was plated in a well of a 6-well plate. Previously, 0.5 mL of viral aliquot had been dispensed in plate. Following 48 h of incubation at 37 °C with 5% CO_2_, cells were washed and given fresh regular culture medium. To select for fluorescence-activated cells, fluorescently labeled cells were flow-sorted after further subculture of transduced cells to attain homogenously labeled cell populations.

### Mono- and coculture 3D spheroid experiments

The 18- to 21-day experiments were initiated with fluorescently labeled sensitive and resistant cell lines in different compositions. For CAMA-1 spheroid experiments, as earlier reported^32^, 2000 cells were plated in different proportions (100% CAMA-1 sensitive, 50% CAMA-1 sensitive–50% CAMA-1 resistant, 100% CAMA-1 resistant) in 96-well round-bottom ultra-low attachment spheroid microplate (Corning, Cat. No.: 4520). After 24 h, spheroids were washed and a fresh medium including treatment drugs was applied. Spheroids were treated for a total of 18–21 days with imaging and media change performed every fourth and seventh day of the week. Spheroids were treated with the following drug therapies at specified doses described in “Results” and Figs. 1 and 4: ribociclib (Selleck Chemicals, Cat. No: S7440), estradiol (Peprotech, Cat. No: 5022822), fulvestrant (Selleck Chemicals, Cat. No: S1191). Imaging was performed using Cytation 5 imager (Biotek Instruments) recording signal intensity from brightfield, YFP (for Venus fluorescence), and CFP 450/440 (for Cerulean fluorescence) channels. Raw data processing and image analysis were performed using Gen5 3.05 and 3.10 software (Biotek Instruments). Briefly, the stitching of 2 × 2 montage images and Z-projection of six layers using focus stacking was performed on raw images followed by spheroid area analysis. To quantify growth under these conditions, we measured fluorescence intensity and growth of the spheroid area over the total time of the experiment. For cell count calculations, a standard curve was created by measuring the area of spheroids 24 h after plating at different cell numbers. A resulting equation by fitting a curve to the data was performed by GraphPad Prism 7.02 software (second-order polynomial–quadratic–curve fit used). The whole spheroid area and fluorescence intensity measurements of each population were integrated into the fitted equation, and cell counts for each population were produced from fluorescence intensities relative to spheroid size. All coculture experiments were performed in triplicates.

### Cell number quantification

For CAMA-1 cells, cell numbers were quantified as described previously by Grolmusz et al.^32^. In brief, the relationship of area to cell counts follows a nonlinear curve (Supplementary Fig. 1a), and sensitive and resistant CAMA-1 cells have similar relationships of area to fluorescence (Supplementary Fig. 1b). Cell numbers were estimated by inverting the nonlinear function, with proportions of the two cell types estimated by the normalized relative fluorescence of each wavelength.

To estimate cell numbers for MCF7/LY2 cells, we measured cell numbers and spheroid area for a range of initial fractions of sensitive (S) and resistant (R) cells and fit to a Michaelis–Menten function with maximum value A and half-saturation constant K (Supplementary Fig. 1c). The relationship differs depending on the proportion of S cells. Parameters are K = 2.753 × 10^5^, A = 1.094 × 10^7^ with pure R cells and K = 2.119 × 10^5^ and A = 5.459 × 10^6^ with pure S cells, and K = 2.330 × 10^5^ and A = 7.546e6 × 10^5^ with mixed cultures (Supplementary Fig. 1c: dashed red curve).

To correct for possible differences in per cell fluorescence, we regressed the fluorescence of pure cultures against the known numbers of S and R cells (Supplementary Fig. 1d). We find a slope of 8.04 × 10^4^ for S cells and 1.349 × 10^5^ for R cells and thus estimate that each R cell produces 1.677 times as much fluorescence. To find the numbers of S and R cells in coculture from the area and the fluorescence, we find the total cells by inverting the relationship between cells and area, and the proportion of each cell type from the fraction of fluorescence, with R cells reduced by the weighting factor of 1.677.

To estimate cell numbers for MCF7 cells, we followed the same procedure, finding Michaelis–Menten fits (Supplementary Fig. 1e) with parameters K = 1.772 × 10^5^, A = 9.061 × 10^6^ with pure R cells and K = 5.315 × 10^5^ and A = 1.138 × 10^7^ when there are any S cells in the culture. We correct for fluorescence in the same way (Supplementary Fig. 1f), finding a slope of 8.36 × 10^4^ for S cells and 2.029 × 10^5^ for R cells, and thus estimate that each R cell produces 2.426 times as much fluorescence.

### Liquid chromatography-tandem mass spectrometry (LC-MS/MS)

Media samples taken on day 21 from 3D spheroid experiments, treated with or without ribociclib (experimental setup previously described in “Results” and “Methods”—“Mono- and coculture 3D spheroid experiments”), and plated in different compositions (100% sensitive, 50% sensitive–50% resistant, and 100% resistant) were spun down at 300 g and frozen at −80 °C. Samples were then prepared by the Analytical Pharmacology Core of City of Hope National Medical Center for LC-MS/MS for estrone and estradiol detection. LC-MS/MS system consisted of a Shimadzu Prominence HPLC system interfaced to an AB SCIEX QTRAP® 5500 system (Foster City, CA, USA). HPLC separation was achieved using an XSELECT CSH Phenyl-Hexyl 3.5 µm, 2.1 × 150 mm analytical column (Waters). The column temperature was maintained at 50 °C, and the flow rate was 0.38 mL/min. The mobile phase consisted of A (Water: 1000 ml + 60 µL 30% NH_4_OH) and B (Methanol: 1000 mL + 60 µL 30% NH_4_OH). The following gradient program was used: 55% B (0.01 min), 70% B (0.01–4.0 min), 100% B (5.5 min), 30%B (8.5 min). The total run time was 8.5 min. The auto-injector temperature was maintained at 15 °C. The atmospheric pressure chemical ionization (APCI) source of the mass spectrometer was operated in negative ion mode with ion source gas (55), curtain gas (20), collision gas (High), and nebulizer current −4.0. The entrance potential was set to −10V. Declustering potential (DP) was −110, collision energy (CE), and collision cell exit potential (CXP) was optimized to −50V, −21V for Estrone, −160V, −50V, −17V for Estrone IS (internal standard), −210V, −58V, −19V for Estradiol, and −205V, −52V, −13V for Estradiol IS respectively. The source temperature was 400 °C. A solvent delay program was used from 0 to 3.5 min and from 6.5 to 8.5 min to minimize the mobile phase flow to the source. Analyst software version 1.5.1 was used for data acquisition and processing. Atmospheric pressure chemical ionization of Estrone, Estrone D4, Estradiol, and Estradiol D5 produced abundant protonated molecular ions (MH-) at m/z 268.980, 272.983, 270.969, and 275.981 respectively. Fragmentation of these compounds was induced under collision-induced dissociation conditions. The precursor→product ion combinations at m/z 268.980→145.200 for Estrone and 272.983→147.100 for Estrone IS. 270.969→182.800 and 275.981→147.000 for Estradiol and Estradiol IS were used in multiple-reaction monitoring mode for quantitation. Under optimized assay conditions, the retention times for Estrone, Estrone IS and Estradiol, Estradiol IS were 4.89, and 4.60 min, respectively.

### Sequencing and bioinformatic analysis

For bulk RNA sequencing, parental sensitive CAMA-1 and CAMA-1_ribociclib_resistant cell lines were plated at 500,000 cells/well in a 6-well plate in triplicates. Twenty-four hours after plating 1 µM ribociclib or vehicle (dimethyl sulfoxide, DMSO) treatment was applied for 12 h, after which cells were trypsinized, washed and the pellet was frozen at −80 °C for subsequent RNA isolation. RNA was isolated using the RNeasy Plus Mini Kit (Qiagen, Cat. No.: 74136) following the manufacturer’s protocol. RNA-seq libraries were prepared using Illumina TruSeq Stranded Total RNA library Prep Ribo-zero Gold following the manufacturer’s protocol. Libraries were sequenced with biological triplicates on an Illumina NovaSeq6000 instrument with 2×150 paired-end reads resulting in an average of 25 million reads per sample. Samples were aligned to the human reference genome (hg19) using the STAR (v2.7.0) aligner. Transcripts were quantified by RSEM (v1.3.1) and library read depth was normalized with edgeR (v3.40.2) using TMM normalization. Log_2_ CPM transformed counts were used for ssGSEA pathways analysis using the R packages GSVA (v1.30.0)^61^. Genes with at least a twofold change in expression with FDR ≤ 0.05 after a two-sided Welch *t*-test were considered statistically significant. Signature scores were generated using the Molecular Signatures Database (v6) Hallmark signature sets. Pathway enrichment with global *p*-value < 0.05 and FDR < 0.25 were considered statistically significant. Differentially expressed genes were also subjected to pathway analysis regarding the Biocarta pathways using DAVID Bioinformatics Resources. In this analysis, an FDR-corrected *p*-value < 0.05 was considered statistically significant.

For single-cell RNA sequencing, spheroids of different compositions (100% sensitive, 50% sensitive–50% resistant, 100% resistant) were initiated from Venus-labeled and mCherry-labeled CAMA-1_ribociclib_resistant cells and were subjected to 1 µM ribociclib treatment. After 11 days, spheroids were harvested, washed and cell suspensions were viably frozen for further processing. Once thawed, cells were centrifuged at 300×*g* and washed twice with 37 °C pre-warmed 1x PBS, pH 7.4 (Gibco, Cat #10010) + 0.04% Nuclease-Free Bovine Serum Albumin (BSA, EMD Millipore, Cat # 12661525 mL). Cells were resuspended to a target concentration of 1000 cells/µL, concentrations were confirmed using trypan blue staining and counted on a hemocytometer. scRNAseq was performed on resuspended cells using the 10X Genomics Chromium Single Cell 3’ GEM, Library & Gel Bead Kit v3 (10X Genomics, Cat # 1000075) according to manufacturer instructions at a target of 10,000 cells per sample. Each sample was barcoded with a unique i7 Index during library preparation using the Chromium i7 Multiplex Kit (10X Genomics, Cat# 120262) to allow sample multiplexing during sequencing. Libraries were sequenced by Fulgent Genetics, on an Illumina HiSeq X instrument with 2×150 paired-end reads and a read depth of 10,000 reads per cell; additional sequencing was performed to increase read depth to a total depth of 50,000 reads per cell. Sequence reads were processed with CellRanger v3.0.2 using a reference genome (GRChg37). A gene-barcode matrix was generated for each sample containing counts of unique molecular identifiers (UMIs) for each gene in each barcode (cell). The matrix was processed with Seurat v3.1.1.9023^62^ to identify cell populations. A series of filters were applied to the data before performing clustering. First, high-quality cells were retained by using the following filters in Seurat: subset = nFeature_RNA < 7000 & nFeature_RNA > 3000 & nCount_RNA > 2000 & nCount_RNA < 60000 & percent.mt < 30. Second, doublet cells were predicted with scrublet (threshold = 0.25)^63^ and the predicted doublets were removed from further analysis. Third, cells expressing mCherry or mVenus were retained by using cells having two or more UMI counts of either mCherry or mVenus. The filtered UMI count matrix was normalized with method “LogNormalize” and “scale.factor=10000”. The top variable 2000 genes were identified and were used to perform Principal Component Analysis (PCA) and clustering in Seurat. Cell clusters were visualized using Uniform Manifold Approximation and Projection (UMAP). Differential expressed genes between cell populations were identified using MAST^64^ as implemented in Seurat using FindMarkers function (abs(foldchange) >= 0.2 & Adjust *p*-value <= 0.05). Genes were ranked based on fold change. Pathway analyses were performed on 50 hallmark signatures (MSigDB, hallmark)^65^ and 4725 curated pathway signatures (MSigDB, c2) using single samples gene set enrichment analysis implemented in the R package GSVA^61^. Significant differential pathway activity was identified using the Wilcoxon rank-sum test.

### Western blot analysis

Lysates of CAMA-1 cells were separated by SDS-polyacrylamide gel electrophoresis and proteins were transferred electrophoretically to a polyvinylidene difluoride membrane using Invitrogen iBlot 2 device and Invitrogen iBlot Transfer Stacks. Membranes were blocked with Tris-buffered saline with 0.05% tween 20 (TTBS) and 5% BSA for 1 h at room temperature. After washing with TTBS, membranes were then probed with anti-aromatase (Invitrogen, MA5-32628, 1:7000 dilution, overnight 4 °C), anti-HSD17β1 polyclonal antibody (Abnova, H00003292-M03A, 1:1000 dilution, overnight 4 °C; R&D systems, MAB7178, 1:2000 dilution, overnight 4 °C), anti-HSD17β8 polyclonal antibody (Proteintech, 16752-1-AP, 1:1000 dilution, overnight 4 °C), anti-ER (Cell Signaling, 8644S, 1:3000 dilution, overnight 4 °C), anti-phospho-ER (Cell Signaling, 2511S, 1:500 dilution, overnight 4 °C) and anti-β-actin monoclonal antibody (Santa Cruz Biotechnology, sc-47778, 1:500 dilution, 1 h room temperature) and detected using SuperSignal West Pico PLUS Chemiluminescent Substrate (Thermo Scientific) with anti-rabbit (GE Healthcare NA9341ML) or anti-mouse (GE Healthcare NXA9311ML) peroxidase-linked secondary antibody (1:6000 dilution). The molecular weight was determined using a pre-stained protein marker (BioRad). Western blots were performed in triplicates. Uncropped and unprocessed images are found in the source data file provided with this paper.

### Estradiol production and uptake analysis

To estimate the production and use of estradiol by sensitive and resistant cells, we compared the fits of three functions, each based on Michaelis–Menten kinetics, by minimizing the least squares difference from the observed estradiol concentrations across cultures with varying resistant and sensitive cell abundances. The full model includes separate production and uptake rates for each cell type. We estimate the production parameters ${\rho }_{R}$ and ${\rho }_{S}$ and the uptake parameters ${a}_{R}$ and ${a}_{S}$, where $R$ and $S$ represent the measured cell numbers, with the function:

1 $${f}_{4}=\frac{{{\rho }_{R}R+\rho }_{S}S}{1+{a}_{R}R+{a}_{S}S}$$

We examined simpler models with fewer parameters. To test a model without production by sensitive cells, we set ${\rho }_{S}=0$, giving:

2 $${f}_{3}=\frac{{\rho }_{R}R}{1+{a}_{R}R+{a}_{S}S}.$$

Because uptake by cells is large compared with background usage (scaled to 1 in each of these models), we tested a simplified model with background usage set to zero. Without that term, the model is overparameterized, and we scale ${a}_{R}=1$. In this case, ${a}_{S}$ represents per cell uptake by sensitive cells relative to resistant cells, and the function simplifies to:

3 $${f}_{2}=\frac{{{\rho }_{R}R+\rho }_{S}S}{R+{a}_{S}S}.$$

During model fitting, we eliminated one outlier (Sample F8). We used the reported lower limit of detection of LLOD = 62.5 and set all measured values of 0 to LLOD/2. Neglecting this adjustment leads to significantly worse fits.

Model ${f}_{3}$ provided a poor fit. The best fit with model ${f}_{4}$ estimated ${\rho }_{R}=83.58$, ${\rho }_{S}=10.11$, ${a}_{R}=0.864$, ${a}_{S}=0.345$ and had a residual sum of squares of 7321.1. The best-fit parameters of model ${f}_{2}$ yielded the same residual sum of squares, with estimates ${\rho }_{R}=96.79$, ${\rho }_{S}=11.71$, ${a}_{S}=0.400$. Due to the equally good fit with one fewer parameter, we choose this as our final model.

To find confidence limits, we used the residual sum of squares to estimate the variance and convert the least squares into a likelihood. For each parameter, we identified the range of values with log likelihood within 2 of the maximum. With model ${f}_{2}$, we found limits ${\rho }_{R}\in \left[{{{{\mathrm{91.49,102.08}}}}}\right]$, ${\rho }_{S}\in \left[{{{{\mathrm{9.88,13.54}}}}}\right]$, ${a}_{S}\in [{{{{\mathrm{0.355,0.460}}}}}].$

### Mathematical modeling—FACT analysis

#### Facilitation analysis through combination therapy

The FACT algorithm breaks into six steps, which we outline here before describing in detail. Modifiers refer to estrogen pathway-modifying treatments, including estradiol itself and three ER antagonists: fulvestrant, tamoxifen, and raloxifene. In each step, we build on the parameters from earlier steps as a null model and ensure that parameters are identifiable.

Algorithm steps:

1. **Fit growth and carrying capacity** for each cell type in monoculture with no treatment.

2. **Treatment effects:** Using the carrying capacity in the absence of treatment, find the effects of ribociclib or modifier treatment on cell growth and death rates (“treatment cost arrow” in Fig. 1e). We make the assumption that carrying capacity is unchanged to focus on the primary effects of treatment on growth.

3. **Synergy:** From growth with combined ribociclib and modifier treatment, compare observed growth with that expected under a null model without interaction.

4. **Competitive effect:** Using the carrying capacities and growth in sensitive and resistant cells in monoculture from Step 1, estimate competition coefficients between the two cell types in coculture (“competition cost” arrow in Fig. 1e).

5. **Facilitation:** Using the carrying capacities, growth, and death rates of single cell types with or without ribociclib from Steps 1 and 2, and the competition coefficients in the absence of treatment from Step 3, quantify facilitation as deviations of growth from predicted under a null model (“facilitation arrow” in Fig. 1e, the difference between observed growth and the null model). To quantify facilitation in the presence of a modifier we follow the same steps but with modifier treatment.

6. **Facilitation modification:** Using the growth of cells in competition with ribociclib (Step 5) and the direct effects of the modifier (Step 2), quantify whether the modifier enhances or reduces facilitation.

#### Mathematical methods

We fit each model with least squares, using all three replicates and the observed mean on day 4 for the initial condition. We exclude the measurements before day 4 because the cells often show a lag before beginning their growth. Models that include a lag require additional parameters and did not provide improved fits or additional insight^66^.

**1. Fit growth and carrying capacity to single-cell data in the absence of treatment**. We use the logistic model, as defined by the differential equations for sensitive and resistant cells

$$\frac{{{{{{\rm{dS}}}}}}}{{{{{{\rm{dt}}}}}}}={{{{{{\rm{r}}}}}}}_{{{{{{\rm{S}}}}}}}\left(1-\frac{{{{{{\rm{S}}}}}}}{{{{{{{\rm{K}}}}}}}_{{{{{{\rm{S}}}}}}}}\right){{{{{\rm{S}}}}}}$$

4 $$\frac{{{{{{\rm{dR}}}}}}}{{{{{{\rm{dt}}}}}}}={{{{{{\rm{r}}}}}}}_{{{{{{\rm{R}}}}}}}\left(1-\frac{{{{{{\rm{R}}}}}}}{{{{{{{\rm{K}}}}}}}_{{{{{{\rm{R}}}}}}}}\right){{{{{\rm{R}}}}}}$$

to estimate *r*_*S*_, *r*_*R*_, *K*_*S*_, and *K*_*R*_ as the growth rates and carrying capacities of sensitive (S) cells and resistant (R) cells.

**2. Treatment and modifier effects:** Using the carrying capacity in the absence of treatment we estimate growth with treatment (*r*_*ST*_, *r*_*RT*_) as shown, and with modifier (*r*_*SM*_, *r*_*RM*_) with the model

$$\frac{{{{{{\rm{dS}}}}}}}{{{{{{\rm{dt}}}}}}}={{{{{{\rm{r}}}}}}}_{{{{{{\rm{ST}}}}}}}\left(1-\frac{{{{{{\rm{S}}}}}}}{{{{{{{\rm{K}}}}}}}_{{{{{{\rm{S}}}}}}}}\right){{{{{\rm{S}}}}}}$$

5 $$\frac{{{{{{\rm{dR}}}}}}}{{{{{{\rm{dt}}}}}}}={{{{{{\rm{r}}}}}}}_{{{{{{\rm{RT}}}}}}}\left(1-\frac{{{{{{\rm{R}}}}}}}{{{{{{{\rm{K}}}}}}}_{{{{{{\rm{R}}}}}}}}\right){{{{{\rm{R}}}}}}$$

**3. Synergy:** To quantify how ribociclib interacts with modifiers in monoculture, we fit the logistic model using carrying capacities from Step 1, and compare the estimated growth *r*_*S*_ with the null model (with a similar form for *r*_*R*_):

6 $${{{{{{\rm{r}}}}}}}_{{{{{{\rm{S}}}}}}}(\frac{{{{{{{\rm{r}}}}}}}_{{{{{{\rm{ST}}}}}}}}{{{{{{{\rm{r}}}}}}}_{{{{{{\rm{S}}}}}}}})(\frac{{{{{{{\rm{r}}}}}}}_{{{{{{\rm{SM}}}}}}}}{{{{{{{\rm{r}}}}}}}_{{{{{{\rm{S}}}}}}}})$$

**4. Estimate competition coefficients:** Using the estimates of the growth rates and carrying capacities from Step 1, we fit untreated coculture data to a Lotka-Volterra competition model with competition coefficients *α*_*SR*_ and *α*_*RS*_.

$$\frac{{{{{{\rm{dS}}}}}}}{{{{{{\rm{dt}}}}}}}={{{{{{\rm{r}}}}}}}_{{{{{{\rm{S}}}}}}}\left(1-\frac{{{{{{\rm{S}}}}}}+{{{{{{\rm{\alpha }}}}}}}_{{{{{{\rm{SR}}}}}}}{{{{{\rm{R}}}}}}}{{{{{{{\rm{K}}}}}}}_{{{{{{\rm{S}}}}}}}}\right){{{{{\rm{S}}}}}}$$

7 $$\frac{{{{{{\rm{dR}}}}}}}{{{{{{\rm{dt}}}}}}}={{{{{{\rm{r}}}}}}}_{{{{{{\rm{R}}}}}}}\left(1-\frac{{{{{{{\rm{\alpha }}}}}}}_{{{{{{\rm{RS}}}}}}}{{{{{\rm{S}}}}}}+{{{{{\rm{R}}}}}}}{{{{{{{\rm{K}}}}}}}_{{{{{{\rm{R}}}}}}}}\right){{{{{\rm{R}}}}}}$$

**5. Estimate facilitation:** We estimate the strength of facilitation as the deviation between log observed and expected growth. To find expected growth, we use the estimates of the growth rates in monoculture with treatment, carrying capacities from the untreated monoculture, and the competition coefficients from the untreated cocultures. We numerically solve the differential Eq. (7) with these parameters and compare with the observed growth by taking the log of the ratio of predicted and observed.

**6. Facilitation modification:** If the presence of the other cell type enhances growth in the presence of ribociclib, a modifier could cancel or **enhance** this effect. To quantify this, we use the growth parameter from step 4 in the presence of ribociclib and account for the direct effects of the modifier as in step 2.

### Facilitation analysis by combination therapy—mechanistic model

#### Facilitation factor production, flux, and utilization

To describe the mechanisms of facilitation between resistant (${{{{{\rm{R}}}}}}$) and sensitive (${{{{{\rm{S}}}}}}$) cells, we model the intracellular concentration of the facilitation factor (${{{{{\rm{E}}}}}}$) in single cells of each cell type (${{{{{{\rm{E}}}}}}}_{{{{{{\rm{S}}}}}}},\, {{{{{{\rm{E}}}}}}}_{{{{{{\rm{R}}}}}}}=$ intracellular concentration within a single sensitive or resistant cell respectively) and the flux of this factor between cell types via the shared extracellular environment (${{{{{{\rm{E}}}}}}}_{{{{{{\rm{E}}}}}}}$). We describe the intracellular production of this factor by sensitive and resistant cells at rate ${\rho }_{{{{{{\rm{S}}}}}}}$ and ${\rho }_{{{{{{\rm{R}}}}}}}$ and the receptor binding for utilization as a growth-promoting signal at rate ${{{{{{\rm{\mu }}}}}}}_{{{{{{\rm{S}}}}}}}$ and ${{{{{{\rm{\mu }}}}}}}_{{{{{{\rm{R}}}}}}}$. Facilitation factors diffuse between cell types and the extracellular environment at rate ${{{{{\rm{\eta }}}}}}$. Finally, we describe the influx (${{{{{{\rm{\sigma }}}}}}}_{{{{{{\rm{E}}}}}}}$ sources = renewed medium in vitro and endocrine signaling from the ovaries in vivo) and decay (${{{{{{\rm{\delta }}}}}}}_{{{{{{\rm{E}}}}}}}$) of facilitation factors from the external environment. This leads to the following set of differential equations describing cellular and environmental concentrations of facilitation factors:

$$\frac{{{{{{\rm{d}}}}}}{{{{{{\rm{E}}}}}}}_{{{{{{\rm{S}}}}}}}}{{{{{{\rm{dt}}}}}}}={\rho }_{{{{{{\rm{S}}}}}}}+{{{{{\rm{\eta }}}}}}\left({{{{{{\rm{E}}}}}}}_{{{{{{\rm{E}}}}}}}-{{{{{{\rm{E}}}}}}}_{{{{{{\rm{S}}}}}}}\right)-{{{{{{\rm{\mu }}}}}}}_{{{{{{\rm{S}}}}}}}{{{{{{\rm{E}}}}}}}_{{{{{{\rm{S}}}}}}}$$

$$\frac{{{{{{\rm{d}}}}}}{{{{{{\rm{E}}}}}}}_{{{{{{\rm{R}}}}}}}}{{{{{{\rm{dt}}}}}}}={\rho }_{{{{{{\rm{R}}}}}}}+{{{{{\rm{\eta }}}}}}\left({{{{{{\rm{E}}}}}}}_{{{{{{\rm{E}}}}}}}-{{{{{{\rm{E}}}}}}}_{{{{{{\rm{R}}}}}}}\right)-{{{{{{\rm{\mu }}}}}}}_{{{{{{\rm{R}}}}}}}{{{{{{\rm{E}}}}}}}_{{{{{{\rm{R}}}}}}}$$

8 $$\frac{{{{{{\rm{d}}}}}}{{{{{{\rm{E}}}}}}}_{{{{{{\rm{E}}}}}}}}{{{{{{\rm{dt}}}}}}}={{{{{{\rm{\sigma }}}}}}}_{{{{{{\rm{E}}}}}}}+{{{{{\rm{\eta }}}}}}{{{{{\rm{S}}}}}}\left({{{{{{\rm{E}}}}}}}_{{{{{{\rm{S}}}}}}}-{{{{{{\rm{E}}}}}}}_{{{{{{\rm{E}}}}}}}\right)+{{{{{\rm{\eta }}}}}}{{{{{\rm{R}}}}}}\left({{{{{{\rm{E}}}}}}}_{{{{{{\rm{R}}}}}}}-{{{{{{\rm{E}}}}}}}_{{{{{{\rm{E}}}}}}}\right)-{{{{{{\rm{\delta }}}}}}}_{{{{{{\rm{E}}}}}}}{{{{{{\rm{E}}}}}}}_{{{{{{\rm{E}}}}}}.}$$

The equilibrium of this system is:

$${{{{{{\rm{E}}}}}}}_{{{{{{\rm{E}}}}}}}^{*}=\frac{{{{{{{\rm{\sigma }}}}}}}_{{{{{{\rm{E}}}}}}}+{\rho }_{{{{{{\rm{S}}}}}}}{{{{{\rm{S}}}}}}\frac{{{{{{\rm{\eta }}}}}}}{{{{{{\rm{\eta }}}}}}+{{{{{{\rm{\mu }}}}}}}_{{{{{{\rm{S}}}}}}}}+{\rho }_{{{{{{\rm{R}}}}}}}{{{{{\rm{R}}}}}}\frac{{{{{{\rm{\eta }}}}}}}{{{{{{\rm{\eta }}}}}}+{{{{{{\rm{\mu }}}}}}}_{{{{{{\rm{R}}}}}}}}}{{{{{{\rm{\eta }}}}}}{{{{{\rm{S}}}}}}\frac{{{{{{{\rm{\mu }}}}}}}_{{{{{{\rm{S}}}}}}}}{{{{{{\rm{\eta }}}}}}+{{{{{{\rm{\mu }}}}}}}_{{{{{{\rm{S}}}}}}}}+{{{{{\rm{\eta }}}}}}{{{{{\rm{R}}}}}}\frac{{{{{{{\rm{\mu }}}}}}}_{{{{{{\rm{R}}}}}}}}{{{{{{\rm{\eta }}}}}}+{{{{{{\rm{\mu }}}}}}}_{{{{{{\rm{R}}}}}}}}+{{{{{{\rm{\delta }}}}}}}_{{{{{{\rm{E}}}}}}}}$$

$${{{{{{\rm{E}}}}}}}_{{{{{{\rm{S}}}}}}}^{*}=\frac{{\rho }_{{{{{{\rm{S}}}}}}}+{{{{{\rm{\eta }}}}}}{{{{{{\rm{E}}}}}}}_{{{{{{\rm{E}}}}}}}^{*}}{{{{{{\rm{\eta }}}}}}+{{{{{{\rm{\mu }}}}}}}_{{{{{{\rm{S}}}}}}}}$$

9 $${{{{{{\rm{E}}}}}}}_{{{{{{\rm{R}}}}}}}^{*}=\frac{{\rho }_{{{{{{\rm{R}}}}}}}+{{{{{\rm{\eta }}}}}}{{{{{{\rm{E}}}}}}}_{{{{{{\rm{E}}}}}}}^{*}}{{{{{{\rm{\eta }}}}}}+{{{{{{\rm{\mu }}}}}}}_{{{{{{\rm{R}}}}}}}},$$

showing that the intra- and extracellular concentrations depend on the net balance between facilitation factor sources (locally through production by cancer cells ($\rho$) and net diffusion into the medium (${{{{{\rm{\eta }}}}}}$) and externally (${{{{{{\rm{\sigma }}}}}}}_{{{{{{\rm{E}}}}}}}$) through the renewal of medium in vitro or via endocrine supply in vivo) and sinks (intracellular (${{{{{\rm{\mu }}}}}}$) and extracellular (${{{{{{\rm{\delta }}}}}}}_{{{{{{\rm{E}}}}}}}$) decay/sequestration). We assume that intracellular dynamics operate on a faster time scale and thus place the values for ${{{{{{\rm{E}}}}}}}_{{{{{{\rm{S}}}}}}}^{*}$ and ${{{{{{\rm{E}}}}}}}_{{{{{{\rm{R}}}}}}}^{*}$ in quasi-steady state in terms of the more slowly changing external concentration ${{{{{{\rm{E}}}}}}}_{{{{{{\rm{E}}}}}}}^{*}$ and cell numbers S and R^67^. Inhibitors of the production of facilitation factors will reduce the intra and extracellular inputs to ${{{{{\rm{E}}}}}}$ (${{{{{\rm{\sigma }}}}}}$ and $\rho$). In contrast, drugs targeting facilitation factor receptors will reduce the binding of intracellular factors, reducing ${{{{{\rm{\mu }}}}}}$ by some factor.

#### Coupling facilitation factor dynamics with cancer coculture spheroid growth

The cellular level description of facilitation was next scaled to describe the populations of resistant and sensitive cells competing and facilitating in coculture spheroids over time. The resistant and sensitive cells are described as transitioning between proliferative (${{{{{{\rm{P}}}}}}}_{{{{{{\rm{R}}}}}}}$,$\,{{{{{{\rm{P}}}}}}}_{{{{{{\rm{S}}}}}}}$), quiescent (${{{{{{\rm{Z}}}}}}}_{{{{{{\rm{R}}}}}}}$,$\,{{{{{{\rm{Z}}}}}}}_{{{{{{\rm{S}}}}}}}$), and senescent (${{{{{{\rm{X}}}}}}}_{{{{{{\rm{R}}}}}}}$,$\,{{{{{{\rm{X}}}}}}}_{{{{{{\rm{S}}}}}}}$) states.

Resource competition between all cells is described by α$\left({{{{{\rm{P}}}}}},\, {{{{{\rm{Z}}}}}},\, {{{{{\rm{X}}}}}}\right)=1-\mathop{\sum}\limits_{i\in \{S,R\}}\frac{{{{{{{\rm{P}}}}}}}_{{{{{{\rm{i}}}}}}}+\,{{{{{{\rm{Z}}}}}}}_{{{{{{\rm{i}}}}}}}+\,{{{{{{\rm{X}}}}}}}_{{{{{{\rm{i}}}}}}}\,}{\,{{{{{{\rm{K}}}}}}}_{{{{{{\rm{i}}}}}}}\,}$, where competitive ability of each cell type (${{{{{\rm{i}}}}}}\in \{S,R\}$) is determined by the carrying capacity parameter K_i_.

Proliferative cells enter the G1/S phase cell cycle checkpoint at a baseline rate (${{{{{\rm{r}}}}}}$), which is reduced by resource competition and increased by facilitation factor availability (E). This beneficial facilitation factor effect saturates at high concentrations when uptake and binding become rate limited (${{{{{\rm{c}}}}}}$). The proliferation rate of resistant and sensitive cells will be related to the binding of intracellular available facilitation factors $({{{{{{\rm{\mu }}}}}}}_{{{{{{\rm{i}}}}}}}{{{{{{\rm{E}}}}}}}_{{{{{{\rm{i}}}}}}})$. The G1/S phase entry of cells of type ${{{{{\rm{i}}}}}}$ based on competition and the internal concentration of facilitation factors therefore follows:

10 $$\,{{{{{{\rm{G}}}}}}}_{{{{{{\rm{i}}}}}}}={{{{{{\rm{r}}}}}}}_{{{{{{\rm{i}}}}}}}(1+\frac{{{{{{{\rm{\mu }}}}}}}_{{{{{{\rm{i}}}}}}}{{{{{{\rm{E}}}}}}}_{{{{{{\rm{i}}}}}}}}{1+{{{{{\rm{c}}}}}}{{{{{{\rm{\mu }}}}}}}_{{{{{{\rm{i}}}}}}}{{{{{{\rm{E}}}}}}}_{{{{{{\rm{i}}}}}}}}\,){{{{{\rm{\alpha }}}}}}\left({{{{{\rm{P}}}}}},\, {{{{{\rm{Z}}}}}},\, {{{{{\rm{X}}}}}}\right).$$

After entering the cell cycle checkpoint, cells undertake the decision to divide or enter a quiescent state, based on the balance of key regulatory cell cycle promoters and inhibitors. In addition to a baseline quiescence rate (${{{{{{\rm{\lambda }}}}}}}_{{{{{{\rm{i}}}}}}}$), cells are promoted to enter the quiescent state by the cell cycle inhibitor ribociclib (${{{{{\rm{x}}}}}}$), which inactivates the key regulators of the G1/S phase cell cycle checkpoint (CDK4/6), blocking cell cycle progression. We describe the effect of ribociclib in inhibiting cell cycle progression and driving cells into a quiescent state as increasing with drug concentration following:

11 $${{{{{{\rm{q}}}}}}}_{{{{{{\rm{i}}}}}}}\left({{{{{\rm{x}}}}}}\right)=\frac{{{{{{\rm{x}}}}}}}{{{{{{{\rm{k}}}}}}}_{{{{{{\rm{i}}}}}}}+{{{{{\rm{x}}}}}}}\,.$$

No additional quiescence is induced in the absence of treatment (${{{{{{\rm{q}}}}}}}_{{{{{{\rm{i}}}}}}}\left({{{{{\rm{x}}}}}}\right)=0$), complete cell cycle arrest is achieved at very high levels of ribociclib (${{{{{{\rm{q}}}}}}}_{{{{{{\rm{i}}}}}}}\left({{{{{\rm{x}}}}}}\right)=1$), and half-maximal cell cycle arrest is achieved at a dose$\,{{{{{{\rm{k}}}}}}}_{{{{{{\rm{i}}}}}}}$ for cell type ${{{{{\rm{i}}}}}}$. Differences in this parameter between resistant and sensitive cells describe innate resistance to the drug that emerged through selection. When cells enter the G1/S phase checkpoint (at rate ${{{{{{\rm{G}}}}}}}_{{{{{{\rm{i}}}}}}}$) they undertake a binary decision to either i) quiesce at a rate$\,{{{{{{\rm{G}}}}}}}_{{{{{{\rm{i}}}}}}}{{{{{{\rm{q}}}}}}}_{{{{{{\rm{i}}}}}}}\left({{{{{\rm{x}}}}}}\right),$ which increases with the dose of ribociclib, or ii) divide if they do not quiesce (${{{{{{\rm{at}}}}}}\; {{{{{\rm{rate}}}}}}\; {{{{{\rm{G}}}}}}}_{{{{{{\rm{i}}}}}}}\left(1-{{{{{{\rm{q}}}}}}}_{{{{{{\rm{i}}}}}}}\left({{{{{\rm{x}}}}}}\right)\right)$). Following quiescence, we describe the transition of cells into a senescent state at rate ${{{{{{\rm{\varphi }}}}}}}_{{{{{{\rm{i}}}}}}}$ before cell death occurs at rate ${{{{{{\rm{\delta }}}}}}}_{{{{{{\rm{i}}}}}}}$.

Combining these models of competition, facilitation, cell cycle progression, arrest, and cell death yields the following spheroid population model, describing the abundance of proliferative, quiescent, and senescent resistant and sensitive cells in coculture spheroids over time. The dynamics of the extracellular facilitation factor concentration in the spheroid population model is governed by the balance between net secretion by proliferative and quiescent cells (resistant = $\,{{{{{{\rm{\gamma }}}}}}}_{{{{{{\rm{R}}}}}}}$ and sensitive = $\,{{{{{{\rm{\gamma }}}}}}}_{{{{{{\rm{S}}}}}}}$) and its decay (${{{{{{\rm{\delta }}}}}}}_{{{{{{\rm{E}}}}}}}$), giving:

$$\frac{{{{{{\rm{d}}}}}}{{{{{{\rm{P}}}}}}}_{{{{{{\rm{i}}}}}}}}{{{{{{\rm{dt}}}}}}}=({{{{{{\rm{G}}}}}}}_{{{{{{\rm{i}}}}}}}\left(1-{{{{{{\rm{q}}}}}}}_{{{{{{\rm{i}}}}}}}\left({{{{{\rm{x}}}}}}\right)\right)-\,{{{{{{{\rm{G}}}}}}}_{{{{{{\rm{i}}}}}}}{{{{{{\rm{q}}}}}}}_{{{{{{\rm{i}}}}}}}\left({{{{{\rm{x}}}}}}\right)-{{{{{\rm{\lambda }}}}}}}_{{{{{{\rm{i}}}}}}}){{{{{{\rm{P}}}}}}}_{{{{{{\rm{i}}}}}}}$$

$$\frac{{{{{{\rm{d}}}}}}{{{{{{\rm{Z}}}}}}}_{{{{{{\rm{i}}}}}}}}{{{{{{\rm{dt}}}}}}}=\,({{{{{{{\rm{G}}}}}}}_{{{{{{\rm{i}}}}}}}{{{{{{\rm{q}}}}}}}_{{{{{{\rm{i}}}}}}}\left({{{{{\rm{x}}}}}}\right)+{{{{{\rm{\lambda }}}}}}}_{{{{{{\rm{i}}}}}}}){{{{{{\rm{P}}}}}}}_{{{{{{\rm{i}}}}}}}-\,{{{{{{\rm{\varphi }}}}}}}_{{{{{{\rm{i}}}}}}}{{{{{{\rm{Z}}}}}}}_{{{{{{\rm{i}}}}}}}$$

$$\frac{{{{{{\rm{d}}}}}}{{{{{{\rm{X}}}}}}}_{{{{{{\rm{i}}}}}}}}{{{{{{\rm{dt}}}}}}}=\,{{{{{{\rm{\varphi }}}}}}}_{{{{{{\rm{i}}}}}}}{{{{{{\rm{Z}}}}}}}_{{{{{{\rm{i}}}}}}}-\,{{{{{{\rm{\delta }}}}}}}_{{{{{{\rm{i}}}}}}}{{{{{{\rm{X}}}}}}}_{{{{{{\rm{i}}}}}}}$$

12 $$\frac{{{{{{\rm{d}}}}}}{{{{{{\rm{E}}}}}}}_{{{{{{\rm{E}}}}}}}}{{{{{{\rm{dt}}}}}}}=\,\left(\sum {{{{{{\rm{\gamma }}}}}}}_{{{{{{\rm{i}}}}}}}\left({{{{{{\rm{P}}}}}}}_{{{{{{\rm{i}}}}}}}+\,{{{{{{\rm{Z}}}}}}}_{{{{{{\rm{i}}}}}}}\right)\right)-\,{{{{{{\rm{\delta }}}}}}}_{{{{{{\rm{E}}}}}}}{{{{{{\rm{E}}}}}}}_{{{{{{\rm{E}}}}}}}.$$

#### Resistant and sensitive cell coculture spheroid growth and facilitation across drug doses

Resistant and sensitive cells were fluorescently labeled as previously described in “Methods” (“Lentiviral labeling of sensitive and resistant cells”) and replicate populations (*n* = 3) were plated either in monoculture or in 50:50 initial coculture. Cancer spheroids were grown in 3D culture for 21 days with imaging performed at 3- to 4-day intervals and the abundance of each cell type was enumerated as described previously in “Methods” (“Mono- and coculture spheroid experiments”). To explore the consequence of cell–cell interactions of drug response, the replicated mono- and coculture time course experiments were conducted under eight ribociclib concentrations within the EC20–50 range (0, 100, 200, 400, 600, 800, 1000, 2000 nM). Spheroid analysis and cell counts were performed as described previously in “Methods” (“Mono- and coculture spheroid experiments”).

#### Measuring the strength of competitive and facilitative interactions between subclones and vital cellular rates

We measured the strength of competitive and facilitative interactions between subclones, the drug impacts on cell cycle arrest, and the vital cellular rates of proliferation, quiescence, and senescence, by fitting the spheroid population model to the experimental data described above. Given the initial spheroid size and composition and the ribociclib concentration, the spheroid population model projects the abundance of sensitive and resistant cells throughout the experiment based on a given combination of biological rates. A lognormal distribution measurement model was used to evaluate the likelihood of each spheroid abundance observation and weakly informative Bayesian priors were used in all cases (Supplementary Information; Supplementary Table 2). The biological rates of each process in the spheroid population model were inferred with uncertainty, using Bayesian inference and a Hamiltonian Monte Carlo algorithm in STAN^68^ (“Methods” and Bayesian inference report detailed in Supplementary Information; Supplementary Fig. 12–16). Biological rates were identified that yield the most accurate model predictions of the observed spheroid growth trajectories and composition over time and across all drug doses (Supplementary Fig. 16). The HMC algorithm efficiently samples the posterior distribution, describing the likely biological rates given the model, using derivatives of the probability density function. As a result, the inference approach jointly and efficiently: (1) identifies the most probable range of subclone interaction strengths (competition and facilitation), (2) predicts the cellular composition and estradiol concentration throughout the experiments and (3) provides a probabilistic measurement of the likelihood of the hypothesis encoded by the model given the available data.

To quantify the relative contribution of facilitation versus competition to sensitive cell proliferation, we decomposed the output of their G1/S phase entry function (${{{{{{\rm{G}}}}}}}_{{{{{{\rm{Sensitive}}}}}}}$) into contributions from competition (α(P, Z, X)) and facilitation ($\frac{{{{{{{\rm{\mu }}}}}}}_{{{{{{\rm{i}}}}}}}{{{{{{\rm{E}}}}}}}_{{{{{{\rm{i}}}}}}}}{1+{{{{{\rm{c}}}}}}{{{{{{\rm{\mu }}}}}}}_{{{{{{\rm{i}}}}}}}{{{{{{\rm{E}}}}}}}_{{{{{{\rm{i}}}}}}}}$) processes. We calculated these two components across time and under the observed range of ribociclib treatment doses, using the posterior means of parameter estimates and the estimated cell abundances. The effect of facilitation relative to competition on sensitive cell proliferation was then measured using the ratio of the facilitation-and competition components.

#### Model predictions of final spheroid size when modulating the strength of facilitation

To explore the consequences of blocking facilitation, we analyzed the impact on resistant and sensitive cell abundances of reducing the rate of receptor binding with facilitation factors (e.g., estradiol). The most likely values of the receptor binding parameters of each cell type (${{{{{{\rm{\mu }}}}}}}_{{{{{{\rm{S}}}}}}}$ and ${{{{{{\rm{\mu }}}}}}}_{{{{{{\rm{R}}}}}}}$) were reduced by a factor ${{{{{\rm{\zeta }}}}}}$ (between 0 and 80% reduction). Using the model and holding all other parameters constant at their inferred posterior means, the predicted abundance of sensitive and resistant cells after 21 days of coculture was forecasted, assuming continuous facilitation blocking throughout this period.

#### Statistics and reproducibility

No data were excluded from the analyses. Experiments were randomized, controlled, and replicated in triplicate for each treatment. The observed consistency of spheroid growth trajectories across replicate experiments was used to determine the sufficiency of sample size. Investigators were not blinded to allocation during experiments, but outcomes were qualitatively assessed using automated imaging protocols and software. Statistical tests are all two-sided and multiple comparison p-value corrections were applied using an FDR correction.

### Reporting summary

Further information on research design is available in the Nature Portfolio Reporting Summary linked to this article.

## Supplementary information

Supplementary Information

Reporting Summary

Source File

## Data Availability

For bulk sequencing analysis, reads were aligned to the human reference genome 19 (hg19). For the single-cell sequencing analysis, sequence reads were processed using a reference genome (GRChg37). Raw RNA-seq data are available under accession codes GSE143944 (CAMA-1 bulk RNA-Seq—[https://www.ncbi.nlm.nih.gov/geo/query/acc.cgi?acc=GSE143944]) and GSE193278 (CAMA-1 scRNA-Seq—[https://www.ncbi.nlm.nih.gov/geo/query/acc.cgi?acc=GSE193278]). All original experimental datasets produced and involved in the results and conclusions are made available at https://github.com/U54Bioinformatics/FacilitationRibociclibBreast. Source data are provided with this paper.

## Code availability

The custom code used in analyses, mathematical modeling and to produce Figs. 1–5 is available on GitHub at: https://github.com/U54Bioinformatics/FacilitationRibociclibBreast.

## References

[1] Rozeboom B, Dey N, De P (2019). ER+ metastatic breast cancer: past, present, and a prescription for an apoptosis-targeted future. Am. J. Cancer Res..

[2] Kantarjian, H. M. & Wolff, R. A. *The MD Anderson Manual of Medical Oncology* (McGraw Hill Professional, 2011).

[3] Ries, L. et al. *SEER Cancer Statistics Review, 1975–2005* (National Cancer Institute, 2008).

[4] Yu Q (2006). Requirement for CDK4 kinase function in breast cancer. Cancer Cell.

[5] Yu Q, Geng Y, Sicinski P (2001). Specific protection against breast cancers by cyclin D1 ablation. Nature.

[6] Landis MW, Pawlyk BS, Li T, Sicinski P, Hinds PW (2006). Cyclin D1-dependent kinase activity in murine development and mammary tumorigenesis. Cancer Cell.

[7] Eli Lilly and Company. *Endocrine Therapy with or without Abemaciclib (LY2835219) following Surgery in Participants with Breast Cancer (monarchE).* https://www.clinicaltrials.gov/ct2/show/NCT03155997 (2017).

[8] Novartis Pharmaceuticals. *A Trial to Evaluate Efficacy and Safety of Ribociclib with Endocrine Therapy as Adjuvant Treatment in Patients with HR+/HER2- Early Breast Cancer (NATALEE).* https://clinicaltrials.gov/ct2/show/NCT03701334 (2018).

[9] Bertagnolli, M. M. E., DeMichele, A. & Gnant M. *PALbociclib CoLlaborative Adjuvant Study (PALLAS).* https://clinicaltrials.gov/ct2/show/NCT02513394 (2015).

[10] Pfizer Inc*. PENELOPE-B* Trial of *IBRANCE® (Palbociclib) in Early Breast Cancer Did Not Meet Primary Endpoint.* https://www.pfizer.com/news/press-release/press-release-detail/penelope-b-trial-ibrancer-palbociclib-early-breast-cancer (2020).

[11] Johnston S (2019). Randomized phase II study evaluating palbociclib in addition to letrozole as neoadjuvant therapy in estrogen receptor-positive early breast cancer: PALLET trial. J. Clin. Oncol..

[12] Johnston SRD (2020). Abemaciclib combined with endocrine therapy for the adjuvant treatment of HR+, HER2-, node-positive, high-risk, early breast cancer (monarchE). J. Clin. Oncol..

[13] Griffiths JI (2021). Serial single-cell genomics reveals convergent subclonal evolution of resistance as early-stage breast cancer patients progress on endocrine plus CDK4/6 therapy. Nat. Cancer.

[14] Finn RS (2016). Palbociclib and letrozole in advanced breast cancer. N. Engl. J. Med..

[15] Fan W, Chang J, Fu P (2015). Endocrine therapy resistance in breast cancer: current status, possible mechanisms and overcoming strategies. Future Med. Chem..

[16] McGranahan N, Swanton C (2017). Clonal heterogeneity and tumor evolution: past, present, and the future. Cell.

[17] Brady SW (2017). Combating subclonal evolution of resistant cancer phenotypes. Nat. Commun..

[18] Delou JMA, Souza ASO, Souza LCM, Borges HL (2019). Highlights in resistance mechanism pathways for combination therapy. Cells.

[19] Fedele C, Tothill RW, McArthur GA (2014). Navigating the challenge of tumor heterogeneity in cancer therapy. Cancer Discov..

[20] Swanton C (2012). Intratumor heterogeneity: evolution through space and time. Cancer Res..

[21] Franco OE (2016). Altered TGF-alpha/beta signaling drives cooperation between breast cancer cell populations. FASEB J..

[22] Gatenby RA, Brown JS (2020). The evolution and ecology of resistance in cancer therapy. Cold Spring Harb. Perspect. Med..

[23] Heppner GH (1993). Cancer cell societies and tumor progression. Stem Cells.

[24] Pienta KJ, McGregor N, Axelrod R, Axelrod DE (2008). Ecological therapy for cancer: defining tumors using an ecosystem paradigm suggests new opportunities for novel cancer treatments. Transl. Oncol..

[25] Crespi B, Summers K (2005). Evolutionary biology of cancer. Trends Ecol. Evol..

[26] Sieber OM, Tomlinson SR, Tomlinson IP (2005). Tissue, cell and stage specificity of (epi)mutations in cancers. Nat. Rev. Cancer.

[27] Gatenby RA, Vincent TL (2003). Application of quantitative models from population biology and evolutionary game theory to tumor therapeutic strategies. Mol. Cancer Ther..

[28] Oates K, Wilson M (2002). Nominal kinship cues facilitate altruism. Proc. Biol. Sci..

[29] Wilson DS, Pollock GB, Dugatkin LA (1992). Can altruism evolve in purely viscous populations?. Evol. Ecol..

[30] Bruno JF, Stachowicz JJ, Bertness MD (2003). Inclusion of facilitation into ecological theory. Trends Ecol. Evol..

[31] Caldwell MM, Dawson TE, Richards JH (1998). Hydraulic lift: consequences of water efflux from the roots of plants. Oecologia.

[32] Grolmusz VK (2020). Exploiting collateral sensitivity controls growth of mixed culture of sensitive and resistant cells and decreases selection for resistant cells in a cell line model. Cancer Cell Int..

[33] Bronzert DA, Greene GL, Lippman ME (1985). Selection and characterization of a breast cancer cell line resistant to the antiestrogen LY 117018. Endocrinology.

[34] Mullick A, Chambon P (1990). Characterization of the estrogen receptor in two antiestrogen-resistant cell lines, LY2 and T47D. Cancer Res..

[35] Adler, F. R. *Modeling the Dynamics of Life: Calculus and Probability for Life Scientists* (Nelson Education, 2012).

[36] Xu J, Chen Y, Olopade OI (2010). MYC and breast cancer. Genes Cancer.

[37] Hao Y, Baker D, Ten Dijke P (2019). TGF-beta-mediated epithelial-mesenchymal transition and cancer metastasis. Int. J. Mol. Sci..

[38] Shajahan-Haq AN (2014). MYC regulates the unfolded protein response and glucose and glutamine uptake in endocrine resistant breast cancer. Mol. Cancer.

[39] Yu L (2019). Estrogen-independent Myc overexpression confers endocrine therapy resistance on breast cancer cells expressing ERαY537S and ERαD538G mutations. Cancer Lett..

[40] McNeil CM (2006). c-Myc overexpression and endocrine resistance in breast cancer. J. Steroid Biochem. Mol. Biol..

[41] Osborne CK, Schiff R (2011). Mechanisms of endocrine resistance in breast cancer. Annu. Rev. Med..

[42] Jeong Y (2019). EGFR is a therapeutic target in hormone receptor-positive breast cancer. Cell Physiol. Biochem..

[43] Neven P, Sonke GS, Jerusalem G (2021). Ribociclib plus fulvestrant in the treatment of breast cancer. Expert Rev. Anticancer Ther..

[44] Im SA, Lu YS, Tripathy D (2019). Ribociclib and endocrine therapy in breast cancer. Reply. N. Engl. J. Med..

[45] Tripathy D (2018). Ribociclib plus endocrine therapy for premenopausal women with hormone-receptor-positive, advanced breast cancer (MONALEESA-7): a randomised phase 3 trial. Lancet Oncol..

[46] Kalas W (2005). Oncogenes and angiogenesis: down-regulation of thrombospondin-1 in normal fibroblasts exposed to factors from cancer cells harboring mutant ras. Cancer Res..

[47] Hanahan D, Folkman J (1996). Patterns and emerging mechanisms of the angiogenic switch during tumorigenesis. Cell.

[48] Archetti M, Pienta KJ (2019). Cooperation among cancer cells: applying game theory to cancer. Nat. Rev. Cancer.

[49] Cleary AS, Leonard TL, Gestl SA, Gunther EJ (2014). Tumour cell heterogeneity maintained by cooperating subclones in Wnt-driven mammary cancers. Nature.

[50] Naffar-Abu Amara S (2020). Transient commensal clonal interactions can drive tumor metastasis. Nat. Commun..

[51] Archetti M, Ferraro DA, Christofori G (2015). Heterogeneity for IGF-II production maintained by public goods dynamics in neuroendocrine pancreatic cancer. Proc. Natl Acad. Sci. USA.

[52] Kefi S, van Baalen M, Rietkerk M, Loreau M (2008). Evolution of local facilitation in arid ecosystems. Am. Nat..

[53] Bertness MD, Callaway R (1994). Positive interactions in communities. Trends Ecol. Evol..

[54] Simigdala N (2016). Cholesterol biosynthesis pathway as a novel mechanism of resistance to estrogen deprivation in estrogen receptor-positive breast cancer. Breast Cancer Res..

[55] Harada N (1997). Aberrant expression of aromatase in breast cancer tissues. J. Steroid Biochem. Mol. Biol..

[56] Forbes NS, Meadows AL, Clark DS, Blanch HW (2006). Estradiol stimulates the biosynthetic pathways of breast cancer cells: detection by metabolic flux analysis. Metab. Eng..

[57] Platet N, Cathiard AM, Gleizes M, Garcia M (2004). Estrogens and their receptors in breast cancer progression: a dual role in cancer proliferation and invasion. Crit. Rev. Oncol. Hematol..

[58] AlFakeeh A, Brezden-Masley C (2018). Overcoming endocrine resistance in hormone receptor-positive breast cancer. Curr. Oncol..

[59] Gonzalez-Angulo AM, Morales-Vasquez F, Hortobagyi GN (2007). Overview of resistance to systemic therapy in patients with breast cancer. Adv. Exp. Med. Biol..

[60] Pepper JW (2012). Drugs that target pathogen public goods are robust against evolved drug resistance. Evol. Appl..

[61] Hanzelmann S, Castelo R, Guinney J (2013). GSVA: gene set variation analysis for microarray and RNA-seq data. BMC Bioinformatics.

[62] Stuart T (2019). Comprehensive integration of single-cell data. Cell.

[63] Wolock SL, Lopez R, Klein AM (2019). Scrublet: computational identification of cell doublets in single-cell transcriptomic data. Cell Syst..

[64] Finak G (2015). MAST: a flexible statistical framework for assessing transcriptional changes and characterizing heterogeneity in single-cell RNA sequencing data. Genome Biol..

[65] Liberzon A (2015). The Molecular Signatures Database (MSigDB) hallmark gene set collection. Cell Syst..

[66] Baranyi J, Roberts TA (1994). A dynamic approach to predicting bacterial growth in food. Int. J. Food Microbiol..

[67] Dockery JD, Keener JP (2001). A mathematical model for quorum sensing in *Pseudomonas aeruginosa*. Bull. Math. Biol..

[68] Carpenter B (2017). Stan: a probabilistic programming language. J. Stat. Softw..

