# Supplementary material for: Cell facilitation promotes growth and survival under drug pressure in breast cancer
Source: Nat Commun. 2023 Jun 29;14:3851. doi: 10.1038/s41467-023-39242-6 (PMC10310817; doi:10.1038/s41467-023-39242-6)
Supplement: Supplementary file 1 — Supplementary Information [file 41467_2023_39242_MOESM1_ESM.pdf]

Supplemental Information

Methods

Cell lines and reagents

The previously authenticated (Laragen; February 1, 2023) estrogen-receptor-positive (ER+) LY2 and MCF7 breast cancer cell lines (ATCC Cat # HTB-22) were maintained in DMEM+ 10% FBS+ 1% antibiotic–antimycotic solution. Ribociclib-resistant LY2 and MCF7 cell lines were created. Briefly, cells were cultured and continuously treated with ribociclib (Selleck Chemicals, Cat. No: S7440) at 1 µM for 1 month for LY2 cells and 7 weeks for MCF7 cells. Following the initial 1 µM ribociclib treatment, LY2 cells were then treated with 2 uM for 4 months while MCF7 cells were treated with 1.5 µM ribociclib for 3 months to develop resistance. Maintenance of ribociclib-resistant LY-2 cells continued in complete culture medium + 2 uM ribociclib. Maintenance of ribociclib-resistant MCF7 cells continued in complete culture medium + 1.5 µM ribociclib. Resistance against ribociclib was confirmed by the alteration of the dose–response curve measured using CellTiterGlo Chemoluminescent Kit (Promega Corporation, Cat. No.: G7573).

Lentiviral labelling of sensitive and resistant cells

Using lentiviruses incorporating distinct fluorescent proteins, we labeled parental sensitive cells (venus; LeGO-V2), ribociclib resistant LY2 cells (cerulean; LeGO-Cer2), and ribociclib resistant MCF7 cells (mCherry; LeGO-C2). LeGO-V2, LeGO-Cer2, and LeGO-C2 vectors were provided by Boris Fehse (Addgene plasmids #27340, #27338, and #27339). Lentiviruses with fluorescent proteins were created using Lipofectamine 3000 reagent (Thermo Fisher Scientific) following manufacturer's protocol. LY2 and MCF7 sensitive and resistant cell lines were transduced with lentivirus using reverse transduction. Briefly, 1mL of polybrene-containing cell suspension of 200,000 cells were plated in a well of a 6-well plate. Previously, 0.5 mL of viral aliquot had been dispensed in plate. Following 48 hours of incubation at 37 ºC with 5% CO_2, cells were washed and given fresh regular culture medium. To select for fluorescence-activated cells, fluorescently labeled cells were sorted after further subculture of transduced cells attain homogenously labeled cell populations.

Mono- and coculture 3D spheroid experiments

The 18-21 day experiments were initiated with fluorescently labeled sensitive and resistant cell lines in different compositions. For LY2 spheroid experiments, 5000 cells were plated in different proportions (100% LY2 sensitive, 80% LY2 sensitive —20% LY2 resistant, 100% LY2 resistant) in 96-well round-bottom ultra-low attachment spheroid microplate (Corning, Cat. No.: 4520) while 5000 cells were plated in different proportions (100% MCF7 sensitive, 90% sensitive – 10% resistant, 100% MCF7 resistant) for MCF7 spheroid experiments. After 24 hours, spheroids were washed and fresh medium including treatment drugs was applied. Spheroids were treated for a total of 18-21 days with imaging and media change performed at every 4th and 7th day of the week. All 3D experiments were performed in DMEM complete medium except for charcoal stripped FBS medium experiments. Spheroids were treated with ribociclib (Selleck Chemicals, Cat. No: S7440), tamoxifen (Selleck Chemicals, Cat. No: S7827), raloxifene (Selleck Chemicals, Cat. No: S5781), exemestane (Selleck Chemicals, Cat. No: S1196), and letrozole (Selleck Chemicals, Cat. No: S1235) at specified doses described in

Supplementary Figure 9. Imaging was performed using Cytation 5 imager (Biotek Instruments) recording signal intensity from brightfield, YFP (for Venus fluorescence) CFP 450/440 (for Cerulean fluorescence) and Texas Red (for mCherry fluorescence) channels. Raw data processing and image analysis were performed using Gen5 3.05 and 3.10 software (Biotek Instruments). Briefly, the stitching of 2 × 2 montage images and Z-projection of 6 layers using focus stacking was performed on raw images followed by spheroid area analysis. To quantify growth under these conditions, we measured fluorescence intensity and growth of spheroid area over the total time of the experiment. For cell count calculations, a standard curve was created by measuring spheroid and fluorescent intensity 24 hours after plating at different cell numbers. To predict cell numbers of other samples, a polynomial equation was fitted to the standard curve data using GraphPad Prism 7.02 software (second order polynomial – quadratic – curve fit used). Whole spheroid area and fluorescence intensity measurements of each population were integrated into the fitted equation, and cell counts for each population were produced from fluorescence intensities relative to spheroid size. All coculture experiments were performed in triplicates.

Western blot analysis

Lysates of LY2 and MCF7 cells were separated by SDS-polyacrylamide gelelectrophoresis and proteins were transferred electrophoretically to a polyvinylidene difluoride membrane using Invitrogen iBlot 2 device and Invitrogen iBlot Transfer Stacks. Membranes were blocked with Tris-buffered saline with 0.05% tween 20 (TTBS) and 5% BSA for 1 hour at room temperature. After washing with TTBS, membranes were then probed with anti-aromatase (Invitrogen, MA5-32628, 1:7000 dilution, overnight 4°C), anti-HSD17β1 polyclonal antibody (Abnova, H00003292-M03A, 1:1000 dilution, overnight 4°C; R&D systems, MAB7178, 1:2000 dilution, overnight 4°C), anti-HSD17β8 polyclonal antibody (Proteintech, 16752-1-AP, 1:1000 dilution, overnight 4°C), anti-ER (Cell Signaling, 8644S, 1:3000 dilution, overnight 4°C), anti-phospho-ER (Cell Signaling, 2511S, 1:500 dilution, overnight 4°C) and anti-β-actin monoclonal antibody (Santa Cruz Biotechnology, sc-47778, 1:500 dilution, 1 hour room temperature) and detected using SuperSignal West Pico PLUS Chemiluminescent Substrate (Thermo Scientific) with anti-rabbit (GE Healthcare NA9341ML) or anti-mouse (GE Healthcare NXA9311ML) peroxidase-linked secondary antibody (1:6000 dilution). The molecular weight was determined using a prestained protein marker (BioRad). Western blots were performed in triplicates.

Multiplex cytokine analysis

Media samples taken at day 21 from 3D spheroid experiments, treated with or without ribociclib, (experimental setup previously described in results and methods - ***Mono- and coculture 3D spheroid experiments***) and plated in different compositions (100% sensitive, 50% sensitive – 50% resistant, and 100% resistant) were spun down at 300g and frozen at -80 ºC. Samples were then prepared by the Analytical Pharmacology Core of City of Hope National Medical Center for multiplex cytokine analysis. Samples were analyzed for 5 cytokines (EGF, FGF2, FGF21, FGF23, and TGFα) using the "ProcartaPlex Multiplex Immunoassay Kit" (Invitrogen, Camarillo, CA) per manufacturer's protocol. In addition, TGFβ-1, -2, and -3 were measured using the "Magnetic Luminex Performance Assay Kit" (R&D Systems, Minneapolis MN) also according to manufacturer's instructions. For analysis of TGFβ, the latent proteins required activation to their immunoreactive state prior to detection. Activation was accomplished by adding 20 µl of 1N HCl to 100 µl of sample followed by incubation for 10 minutes at room temperature. Samples were then neutralized by the addition of 20 µl of 1.2N

NaOH/0.5M HEPES prior to dilution and loading on to the plate. Briefly, multiplex bead solutions were vortexed for 30 s and 50 µl was added to each well and then washed twice with wash buffer. Next, 50 µl of sample (cell culture supernatant) was loaded in duplicate into Greiner flat-bottom 96 well microplates. Cytokines standards were reconstituted with unconditioned cell culture medium and serial dilutions were prepared. Plates were then incubated on a plate shaker at 500 rpm in the dark at room temperature for 2 hours. The plate was then applied to a magnetic device designed to accommodate a microplate and all wells were washed two times with 200 µl of wash buffer. Biotinylated detection antibody mix (25 µl) was added to each well and the plate was incubated on the plate shaker for another hour. After washing two times with 200 µl of wash buffer, streptavidin-phycoerythrin (50 µl) was added to each well followed by incubation on a plate shaker for 30 minutes. After two more washes, the contents of each well were resuspended in 120 µl reading buffer and shaken for 5 min. Finally, the plate was transferred to the Flexmap 3D Luminex system (Luminex corp.) for analysis, cytokine concentrations were calculated using Bio-Plex Manager 6.2 software with a five parameter curve-fitting algorithm applied for standard curve calculations for duplicate samples.

Ribociclib concentration measurements with high performance liquid chromatography - mass spectrometry (HPLC/MS)

Spheroid experiments were initiated as earlier described (***Mono- and coculture spheroid experiments*)** with some modifications. CAMA-1 sensitive and resistant cells were plated at different cell numbers (2,000; 10,000; 40,000) and in different compositions (100% sensitive, 50% sensitive – 50% resistant, and 100% resistant). After 24 hours, ribociclib treatment (400nM) was applied for 4 days. Following the 4-day treatment, media from cell samples were spun down at 300g and frozen at -80 ºC. Samples were then prepared by the Analytical Pharmacology Core of City of Hope National Medical Center for HPLC/MS. Media without cells (+/- drug) were also subjected to HPLC/MS measurements.

Acetonitrile (ACN) and methanol were of HPLC-grade and purchased from Fisher Scientific (Fair Lawn, NJ, USA). Ammonium acetate was purchased from Mallinckrodt (Kentucky, USA). Ribociclib and abemaciclib (internal standard) were provided by Selleck Chemicals. Deionized water was prepared using the Millipore Milli-Q system (Milford, MA, USA). Abemaciclib measurements were optimized to provide negative control.

Samples were prepared for analysis by mixing 30 µl of media with 10 µl 50% methanol in water in a 0.5 ml low retention micro-centrifuge tube. To this tube, 10 µl of 6 µM abemaciclib in 50% methanol and 180 µl of ice cold methanol were added. The tube was then vortex mixed for 3 minutes and centrifuged for 5 minutes at highest speed and 4°C. Following centrifugation, 20 µl of the supernatant was mixed with 180 µl of 50% methanol in 50% 10mM ammonium acetate. The final solution was then transferred to an autosampler vial and 2 µl was injected on column.

LC-MS/MS analysis was performed using a Waters Acquity UPLC system (Milford, MA, USA) interfaced with a Waters Quattro Premier XE mass spectrometer. HPLC separation was achieved using a Gemini NX C18, 100 x 2.1mm column (Phenomenex, Torrance, CA, USA) proceeded by a Phenomenex Gemini NX C18 guard column (Torrance, CA, USA). The column temperature was maintained at 40°C. Isocratic elution was performed using a mobile phase of 15% 6 mM ammonium acetate in ACN at a flow rate of 0.3 ml/min. Under optimized

chromatographic conditions, the retention times were 1.2 minutes and 1.89 minutes for ribociclib and abemaciclib, respectively. The total run time was 4 minutes.

The electrospray ionization source of the mass spectrometer was operated in positive ion mode with a cone gas flow of 25 L/hr and a desolvation gas flow of 750 L/hr. Capillary voltages were 0.4 kV for both ribociclib and abemaciclib, and the cone voltages were optimized at 45 V for ribociclib and 28 V for abemaciclib, respectively. The collision voltages were set to 32 V for ribociclib and 28 V for abemaciclib. The source temperature was 125°C and the desolvation temperature was 470°C. Multiple reaction monitoring (MRM) was used for quantitation and the optimal precursor → product ion combinations were determined to be 435.34 → 321.9 m/z and 507.32 → 392.92 m/z for ribociclib and abemaciclib, respectively. MassLynx version 4.1 software was used for data acquisition and processing.

Total exosome isolation experiment

Spheroid experiments were initiated as earlier described (***Mono- and coculture spheroid experiments***) with some modifications. CAMA-1 sensitive and resistant cells were plated at 2,000 cells per well and in different compositions (100% sensitive and 100% resistant). These spheroids were treated with or without 400nM ribociclib and designated as donor wells. On every 3rd and 6th day of the week media was changed on the donor wells and exosomes were isolated from the old media using Total Exosome Isolation Reagent (from cell culture media, Thermo Fisher, Cat. No.: 4478359) according to the manufacturer's protocol. Briefly, cell culture media was centrifuged at 2000Xg for 30 minutes to remove cells and debris, and 200 uL supernatant was mixed with 100 uL Total Exosome Isolation Reagent and incubated on 4C overnight. Following this incubation, samples were centrifuged at 10,000Xg for 60 minutes at 4C and exosomes were resuspended in PBS. Exosomes (either from untreated or ribociclib treated, 100% sensitive or 100% resistant donor wells) were isolated from this procedure and added to the media of other 100% sensitive spheroids, designated as acceptor wells, during media change on every 4th and 7th day of the week. During this media change, 75% of media was untreated or 400nM ribociclib treated complete media with the remaining 25% contribution of isolated exosomes originating from donor wells. Imaging of each well was performed on every 4th and 7th day of the week as earlier described in methods using the Cytation 5 imaging system. Analysis was completed as previously described in methods.

Differentially expressed genes between mono-culture and sensitive mono-culture treated with ribociclib

To identify genes that are upregulated in resistant cells in monoculture, we used the ‘FindMarkers’ function from the Seurat package (version 4.3.0) with sensitive cells in mono-culture as baseline. The Wilcoxon Rank Sum method was used to identify statistically significant genes. Genes were only considered for analysis if expression was detected in a minimum of 25% of cells. To be considered statistically significant, an average log-fold change of 0.5 and adjusted p-value of 0.05 were used as thresholds. Positive fold-change values represented genes enriched in resistant cells while negative fold-change values represented genes enriched in sensitive cells.

Description of FACT mechanistic model comparisons

Using formal model comparison, we compared the accuracy of our facilitation model's predictions of spheroid growth against predictions of alternative models describing direct competition for resources (Competition alone model) or phenotypic plasticity in which cells transition from a naive to a resistant state either in response to drug induction (Plasticity DI) or via random switching (Plasticity RS). Here we outline the set of differential equation models used to describe alternative hypotheses of mechanisms that govern the growth dynamics of monocultures and cocultures of resistant and sensitive cells (CAMA-1) grown under different doses of the cell cycle inhibitor ribociclib. Bayesian inference was used to fit each model to the growth trajectories of mono- and co-cultures of sensitive and resistant cells across 8 doses of ribociclib. We then present the model comparison results showing which hypotheses about the mechanisms of cell-cell interactions were supported by the data.

Defining alternative models describing hypothesized mechanisms governing spheroid growth dynamics

Competition alone

Populations of sensitive (S) and resistant (R) cells compete for resources to proliferate. The abundance of cell type i ($i \in \{S, R\}$) within a spheroid (N_i) depends on the balance of cell proliferation and death. Sensitive and resistant cells each divide at a baseline rate (r_i) that is reduced by cell cycle inhibition (ribociclib; x). The susceptibility of each cell type to cell cycle inhibition depends on its β_i parameter. Proliferation is also reduced through competition, with each cell type having a competitive effect equal to $1/K_j$. Finally cell death occurs at rate δ_i. This yields the following competition model:

$$\frac{dN_i}{dt} = \frac{r_i}{(1 + \beta_i x)}\left(1 - \sum_{1}^{n} \frac{N_j}{K_j}\right) N_i - \delta_i N_{i_X}$$

Plasticity (DI): Drug induced phenotype switching

Treatment may stimulate modified gene expression, inducing cells to transition to a more resistant state, perhaps through epigenetic reprogramming. We describe both the innately resistant and sensitive cells as transitioning from a naive state (N_i) to an induced resistant state ($\widetilde{N}_i$). This transition rate (λ) is proportional to drug concentration. As in the competition alone model (above), proliferation depends on the ribociclib concentration and the abundance and competitiveness of naïve and induced cells of each lineage (resistant vs sensitive). Baseline proliferation and ribociclib susceptibility are allowed to differ between innately resistant and sensitive cells (r_i and β_i, with $i \in \{S, R\}$ and for each cell type when in a state of induced resistance ($\tilde{r}_i$ and $\tilde{\beta}_i$, with $i \in \{S, R\}$). Cell death occurs at differing rates in induced and un-induced cells. This yields the following drug induced phenotype switching model:

$$\frac{dN_i}{dt} = \frac{r_i}{(1 + \beta_i x)}\left(1 - \sum_{j=1}^{n} \left(\frac{N_j}{K_j} + \frac{\widetilde{N}_j}{\widetilde{K}_j}\right)\right) N_i - \delta_i N_i - \lambda x N_i$$

$$\frac{d\widetilde{N}_i}{dt} = \frac{\tilde{r}_i}{(1 + \tilde{\beta}_i x)}\left(1 - \sum_{j=1}^{n} \left(\frac{N_j}{K_j} + \frac{\widetilde{N}_j}{\widetilde{K}_j}\right)\right) \widetilde{N}_i - \tilde{\delta}_i \widetilde{N}_i + \lambda x N_i$$

Plasticity (RS): Random phenotype switching

Resistance related changes in gene expression might occur independent of treatment, for example depending on the cell cycle state that the cell happened to be in at the onset of treatment. We describe the resistant and sensitive lineages as transitioning from a naive state

(N_i) to an induced resistant state ($\widetilde{N}_i$) at a rate that is independent of treatment (λ). Cell proliferation and death is the same as in the drug induced phenotype switching model. This yields the following random phenotype switching model:

$$\frac{dN_i}{dt} = \frac{r_i}{(1+\ \beta_i x)}\left(1 - \sum_{j=1}^{n}(\frac{N_j}{K_j} + \frac{\widetilde{N}_j}{\widetilde{K}_j})\right)N_i -\ \delta_i N_i - \lambda N_i$$

$$\frac{d\widetilde{N}_i}{dt} = \frac{\tilde{r}_i}{(1+\ \tilde{\beta}_i x)}\left(1 - \sum_{j=1}^{n}(\frac{N_j}{K_j} + \frac{\widetilde{N}_j}{\widetilde{K}_j})\right)\widetilde{N}_i -\ \tilde{\delta}_i \widetilde{N}_i + \lambda N_i$$

Facilitation Symmetric (1D): Allee effect model of facilitation

We first describe facilitation between resistant and sensitive cells in which cells of both lineages contribute equally to the facilitation effect on a per cell basis (symmetric facilitation effects). We describe the production of a facilitation factor that is produced at rate ϵ in both cell types. The beneficial effect of the facilitation factor saturates at high concentrations, with the asymptotic benefit at high densities equaling $1/\ c$. This facilitation effect increases proliferation. The functional form of competition, the drug impact and cell death are the same as in the competition model. This yields the following Allee effect model of symmetric facilitation:

$$\frac{dN_i}{dt} = \frac{r_i}{(1+\ \beta_i x)}\left(1 - \sum_{j=1}^{n}\frac{N_j}{K_j}\right)\left(1 + \frac{\epsilon \sum_{j=1}^{n} N_j}{1 + c\ \sum_{j=1}^{n} N_j}\right)N_i -\ \delta_i N_{i_X}$$

Facilitation (1D): Asymmetric contribution to facilitation

The production and decay of a facilitation factor (E_E) can be modelled explicitly to account for the differences in production by sensitive and resistant cells. Cells of each cell type i produce the facilitation factor at rate γ_j and it decays at rate δ_E. The concentration of the facilitation factor determines the proliferation promotion benefit. As with the symmetric facilitation model, this benefit saturates at high concentrations, with an asymptotic benefit of $1/\ c$. The functional form of competition, the drug impact and the cell death are again the same as in the basic competition model. With E_E in quasi steady state and symmetric contributions of cell types to facilitation, this reduces to the Allee effect model of facilitation.

$$\frac{dN_i}{dt} = \frac{r_i}{(1+\ \beta_i x)}\left(1 - \sum_{j=1}^{n}\frac{N_j}{K_j}\right)\left(1 + \frac{E_E}{1 + c\ E_E}\right)N_i -\ \delta_i N_{i_X}$$

$$\frac{dE_E}{dt} = \sum_{j=1}^{n}\gamma_j N_j -\ \delta_E E_E$$

Facilitation (2D): Asymmetric contribution to facilitation & cell quiescence

To describe the mechanism of action of ribociclib treatment more mechanistically, we use the stage-structured modeling approach to create a minimal population level model to predict the effects of cell cycle inhibition treatment (1).

With this approach, cells transition from a proliferative (P), to a quiescent (Z) state. Proliferative cells enter the G1/S phase cell cycle checkpoint at a baseline rate (r), which is reduced by resource competition ($\alpha(P,Z)$) and increased by estradiol availability (E_E). Resource competition between cells is described by $\alpha(P,Z) = 1 - \sum_{j=1}^{n} \frac{P_j + Z_j}{K_j}$, where competitive ability of each cell type (j) is determined by the carrying capacity parameter K_j. As in the 1D facilitation model, the extracellular concentration of the facilitation factor (E_E) depends on its differing net production by sensitive and resistant cells (γ_j) and its decay (δ_E). Additionally, we describe the intracellular concentration of facilitation factors, which depends on the balance of production by the cell and the influx into the cell against diffusion out of the cell and binding. This leads to the intracellular steady state concentration of cell type i of: $E_i = \frac{\rho_i + \eta E_E}{\eta + \mu_i}$.
Cell entry into the G1/S phase checkpoint increases with the intracellular level of estradiol (E_i) and increased receptor binding (μ_i), saturating at high concentrations when uptake and binding becomes rate limited (c). We describe this as: $G_i = r_i(1 + \frac{\mu_i E_i}{1 + c\mu_i E_i})\alpha(P,Z)$. Cells at the G1/S phase checkpoint undertake a decision to divide or enter a quiescent state, based on the balance of key regulatory cell cycle promoters and inhibitors. Cell cycle inhibition using ribociclib (x) inactivates key promoters of the G1/S checkpoint transition, blocking cell cycle progression and increasing the probability of quiescence above the baseline (λ_i) according to $q_i(x) = \frac{x}{k_i + x}$. Here the half-saturation constant (k_i) can differ between resistant and sensitive cells to quantify drug susceptibility. Cells enter the G1/S phase checkpoint (at per cell rate $\mathrm{G_i}$) and undertake a binary decision to either i) quiesce at a rate $\mathrm{G_i q_i(x)}$ (which increases with ribociclib dose) or ii) divide if they do not quiesce at rate $\mathrm{G_i}(1 - \mathrm{q_i(x)})$. Following quiescence, cell death occurs at rate φ_i.

Together these components capturing competition, facilitation, cell cycle progression and arrest yield the population model:

$$G_i = r_i\left(1 - \sum_{j=1}^{n} \frac{P_j + Z_j}{K_j}\right)\left(1 + \frac{\mu_i E_i}{1 + c_i \mu_i E_i}\right)$$

$$q_i(x) = \frac{x}{k_i + x}$$

$$\frac{dP_i}{dt} = (\ G_i(1 - q_i(x)) - \ G_i q_i(x) - \ \lambda_i)P_i$$

$$\frac{dZ_i}{dt} = \ (G_i q_i(x) + \ \lambda_i)P_i - \ \varphi_i Z_i$$

$$\frac{dE_E}{dt} = \ \left(\sum \gamma_i (P_i + \ Z_i)\right) - \ \delta_E E_E.$$

Facilitation (3D): Asymmetric contribution to facilitation & cell quiescence and senescence

Finally, we extend the stage structured model to describe the transition from proliferative (P), to quiescent (Z) and senescent (X) cell states.

Proliferative cells enter the G1/S phase cell cycle checkpoint at a baseline rate (r), which is reduced by resource competition ($\alpha(P,Z,X)$) and increased by estradiol availability (E_E). Resource competition between all three cell states extends to: $\alpha(P,Z,X) = 1 - \sum_{j=1}^{n} \frac{P_j + Z_j + X_j}{K_j}$, where competitive ability of each cell type (j) is determined by the carrying capacity parameter K_j. As above, cell entry into the G1/S phase checkpoint increases with the intracellular level of estradiol (E_i) and increased binding (μ_i), saturating at high concentrations when uptake and binding becomes rate limited (c), which we describe as: $G_i = r_i(1 + \frac{\mu_i E_i}{1+c\mu_i E_i})\alpha(P,Z,X)$. Cells in the G1/S phase checkpoint either divide or enter a quiescent state, following the same mechanism as described in the 2D facilitation model. However, following quiescence, cells transition into the final senescent state at rate φ_i before cell death occurs at rate δ_i. Although structurally and mechanistically similar, this model produces additional delays in the drug effects on population abundances. This full model and its derivation is described in more detail in the methods section but follows:

$$G_i = r_i\left(1 - \sum_{j=1}^{n} \frac{P_j + Z_j + X_j}{K_j}\right)\left(1 + \frac{\mu_i E_i}{1 + c_i \mu_i E_i}\right)$$

$$q_i(x) = \frac{x}{k_i + x}$$

$$\frac{dP_i}{dt} = \left(G_i\big(1 - q_i(x)\big) - G_i q_i(x) - \lambda_i\right)P_i$$

$$\frac{dZ_i}{dt} = (G_i q_i(x) + \lambda_i)P_i - \varphi_i Z_i$$

$$\frac{dX_i}{dt} = \varphi_i Z_i - \delta_i X_i$$

$$\frac{dE_E}{dt} = \left(\sum \gamma_i(P_i + Z_i)\right) - \delta_E E_E.$$

Bayesian inference report: Fitting the Estradiol mediated facilitation model to spheroid growth trajectories

Here we provide details of the inference workflow used to parameterize the Estradiol mediated facilitation model (reported in the main text; Facilitation (3D), as well as the comparison models defined above). Such models are necessarily nonlinear, to incorporate density and dose dependent interaction between cells, dynamical to describe growth trajectories over time and must account for the unmeasured state of the facilitation factor (a latent variable of the model). Posterior parameter distributions of the model (detailed in the methods) were estimated using Markov Chain Monte Carlo (MCMC) simulations. We used the STAN statistical programming language, to conduct efficient Bayesian inference though its implementation of gradient-based MCMC algorithms (Hamiltonian Monte Carlo). Stan was accessed via the “rstan” package (version 2.19.2) in R (2-3).

Priors
Under a given set of parameters, the probability density of observations was evaluated against prior beliefs and the log normally distributed likelihood model. The model parameters were all constrained to be non-negative and were given weak prior with wide distribution to reflect uncertainty. The parameters' description, prior and its rational are presented in ***Supplementary Table 1***. Crucially, for each parameter that was allowed to vary between sensitive and resistant cells (indicated by parameter subscripts) the same prior distribution was applied for each cell type's parameter. This avoided biasing posterior inferences and meant that all differences in between sensitive and resistant cells were informed by the spheroid trajectory data that contribute to the model likelihood. Finally, estimation was performed on log transformed parameter values and a Bayesian shrinkage term (parameter~Normal(mean=prior expectation, sd= 2)) to restrain parameter values within biologically sensible ranges.

MCMC inference
For each model, we ran three independent MCMC chains, each with 4000 warm-up iterations followed by an inference phase of 1000 sampling iterations. This resulted in 3000 posterior samples for each parameter. Diagnostic checks were undertaken to ensure that inferences were reliable. We verified MCMC convergence by ensuring that the independent chains had convergent posterior probability and residual error early in the warmup phase (***Supplementary Figure 12***). To assess the performance of the MCMC inference algorithm, we examined the stability of posterior probability between the warmup and inference phase and verified a sufficient metropolis acceptance rate was obtained (***Supplementary Figure 13***). To verify that MCMC inference iterations represented independent samples of the posterior distribution of parameter space, we assessed the autocorrelation of MCMC states across different lags (***Supplementary Figure 14***). We identified a low correlation of MCMC states with previous iterations (autocorrelation > 0.5 by lag 5). The scale reduction factor of all parameters was assessed to be approximately 1. Finally, density plots were used to ensure parameter convergence of MCMC chains during the inference phase (***Supplementary Figure***). Posterior parameter estimates of each chain were overlapping and considerably narrower than the prior distributions, indicating that parameters were well informed by the data and that posteriors did not simply reflect the prior knowledge. In conclusion, the MCMC chains converged rapidly, were well mixed, stationary and not auto-correlated.

Assessment of posterior parameter estimates
For all biological processes in the model that varied between resistant and sensitive cells, we compared the parameter estimates and their uncertainty (***Supplementary Figure 16***). Our results show that as expected, sensitive cells had higher division rates (r_R) and carrying capacity ($K = 1/\alpha$) (in the absence of treatment). However, sensitive cells also quiesce (λ), senesce (φ) and die (δ) more quickly in the absence of treatment. As expected, ribociclib treatment had a greater effect on sensitive cells, with their half maximum drug dose being lower (k). Resistant cells produced the facilitation factor at a faster rate than sensitive cells (γ). Each biological parameter that varied between cell types (panel) had equal prior distributions for the belief of resistant and sensitive cell rates.

Results of models comparison showing which hypotheses were supported by the data

Formal probabilistic model comparison of the non-nested alternative hypotheses was performed using Watanabe–Akaike information criterion (WAIC). This sums the average fit (log likelihood) of posterior samples over all data points and penalizes the complexity of the

model hypothesis based on the pointwise variance in the fit across posterior uncertainty (4). The resulting WAIC score measures the goodness of fit of the model to the data whilst penalizing for model complexity to avoid overfitting. Models incorporating facilitation greatly outperformed (lower penalized prediction error) models of competition alone or phenotypic plasticity. Facilitation models assuming equivalent facilitation by resistant and sensitive cells (Facilitation symmetric 1-State) were greatly outperformed by facilitation models in which resistant cells contribute disproportionately to the production of estradiol (Facilitation 1/2/3-State). The addition of quiescent and senescent cell states further improved model accuracy compared to simpler models in which ribociclib simply reduces cell division rates (Facilitation 2/3-State versus Facilitation 1-State). These additional states allow the prediction of the delayed impacts of therapy at higher doses (reducing penalized prediction error) and also the estimation of the rate of division and quiescence with and without therapy. Describing cells as transitioning through proliferative, quiescent and senescent states during the cells life (Facilitation 3-State; model schematic in **Fig. S10**) yielded a superior description of the data after penalizing for model complexity. This analysis guarded against model overfitting and showed the explanatory power of the estradiol mediated facilitation hypothesis and the model of this process that we present in the main text.

Gene Set	Theme	Mean Difference	P-value	FDR	Enriched
HALLMARK_MYC_TARGETS_V1	MYC	0.05533864	1.666E-173	1.035E-172	Res.mono
HALLMARK_MYC_TARGETS_V2	MYC	0.021319258	1.1588E-40	2.2852E-40	Res.mono
PID_MYC_ACTIV_PATHWAY	MYC	0.026054634	4.1923E-67	1.0679E-66	Res.mono
REACTOME_SIGNALING_BY_EGFR_IN_CANCER	EGFR	0.011034124	1.3002E-33	2.3829E-33	Res.mono
REACTOME_SIGNALING_BY_CONSTITUTIVELY_ACTIVE_EGFR	EGFR	0.025082555	6.9381E-34	1.2755E-33	Res.mono
REACTOME_TGF_BETA_RECEPTOR_SIGNALING_ACTIVATES_SMADS	TGFB	0.024843515	2.1383E-44	4.3656E-44	Res.mono
JAZAG_TGFB1_SIGNALING_UP	TGFB	0.019763556	2.5362E-76	6.9525E-76	Res.mono
REACTOME_TGF_BETA_RECEPTOR_SIGNALING_IN_EMT_EPITHELIAL_TO_MESENCHYMAL_TRANSITION	TGFB	0.01132874	2.3604E-09	3.0616E-09	Res.mono
HOLLERN_EMT_BREAST_TUMOR_UP	EMT	0.012935462	2.5337E-28	4.3099E-28	Res.mono

***Supp. Table 1*. Enriched biological signatures identified in resistant cells in monoculture.** Single-cell RNA-sequencing data using ssGSEA scores identified several signaling pathways enriched in cells resistant to ribociclib relative to sensitive cells. This analysis revealed three major themes implicated in growth factor signaling, receptor tyrosine kinase signaling, and drug resistance: MYC, EGFR, and TGFβ. Statistically significant gene sets were identified using a non-parametric (two-sided) Wilcox rank-sum test.

Parameter description	Parameter	Prior	Rationale
ln(Sensitive cell baseline division rate)	$\ln(r_S)$	Normal(1e-5, 1)	Experiments showed cell division rate is on order of a day. Weak prior with wide distribution to reflect uncertainty
ln(Resistant cell baseline division rate)	$\ln(r_R)$	Normal(1e-5, 1)	Experiments showed cell division rate is on order of a day. Weak prior with wide distribution to reflect uncertainty
Sensitive cell carrying capacity	$\ln(K_S)$	Normal(1.6e5, 5e4)	This weak prior reflects the max abundance achieved in untreated conditions. Weak prior with wide distribution to reflect uncertainty
Resistant cell carrying capacity	$\ln(K_R)$	Normal(1.6e5, 5e4)	This weak prior reflects the max abundance achieved in untreated conditions. Weak prior with wide distribution to reflect uncertainty
Sensitive cell baseline quiescence	λ_S	Normal(0, 0.5)	Quiescence expected to be slower than division. Weak prior with wide distribution to reflect uncertainty
Resistant cell baseline quiescence	λ_R	Normal(0, 0.5)	Quiescence expected to be slower than division. Weak prior with wide distribution to reflect uncertainty
Sensitive senescent cell death rate	δ_S	Normal(0, 0.5)	Death rate expected to be slower than division. Weak prior with wide distribution to reflect uncertainty
Resistant senescent cell death rate	δ_R	Normal(0, 0.5)	Death rate expected to be slower than division. Weak prior with wide distribution to reflect uncertainty
Sensitive cell facilitation factor production rate	γ_S	Normal(0, 0.5)	Per cell production rate of facilitation factor expected to be slow. Weak prior with wide distribution to reflect uncertainty
Resistant cell facilitation factor production rate	γ_R	Normal(0, 0.5)	Per cell production rate of facilitation factor expected to be slow. Weak prior with wide distribution to reflect uncertainty
Facilitation factor degradation rate	δ_E	Normal(5, 5)	Degradation rate of facilitation factor extracellularly expected to be fast. Weak prior with wide distribution to reflect uncertainty
Sensitive cell facilitation factor uptake and binding	μ_{i_S}	Normal(1, 0)	Per cell facilitation factor uptake rate fixed to unity to scale the other dynamical processes associated with facilitation factor dynamics.
Resistant cell facilitation factor uptake and binding	μ_{i_R}	Normal(1, 0)	Per cell facilitation factor uptake rate fixed to unity to scale the other dynamical processes associated with facilitation factor dynamics.
Sensitive quiescent cell senescence rate	φ_S	Normal(0, 0.5)	Quiescent cells expected to quickly senesce. Weak prior with wide distribution to reflect uncertainty
Resistant quiescent cell senescence rate	φ_R	Normal(0, 0.5)	Quiescent cells expected to quickly senesce. Weak prior with wide distribution to reflect uncertainty

ln(Ribociclib dose giving half maximal cell cycle arrest of sensitive cells)	k_S	Normal(5, 2)	Ribobiclib dose response curves showed the effective dose range to control proliferation was range of 100-1000 nM. Weak prior with wide distribution to reflect uncertainty.
ln(Ribociclib dose giving half maximal cell cycle arrest of resistant cells)	k_R	Normal(5, 2)	Ribobiclib dose response curves showed the effective dose range to control proliferation was range of 100-1000 nM. Weak prior with wide distribution to reflect uncertainty.
Sensitive and resistant cell limitation of facilitation factor uptake and binding	c	Normal(0, 1);	A small amount of facilitation factor would be expected to saturate signal uptake. Weak prior with wide distribution to reflect uncertainty.
ln(Normally distributed noise standard deviation)	σ	Normal(-3, 2)	Experimental data was highly reproducible. We expected an accurate model of the system to fit the data with low residual error.

***Supp. Table 2.* List of prior expectations for estradiol mediated facilitation model parameters. Table** includes rational for prior expectations for each parameter allowed to vary between sensitive and resistant cells (indicated by parameter subscripts) (Eq.6; Fig 5). The same prior distribution was applied for each cell type's parameter to avoid biasing posterior inferences.

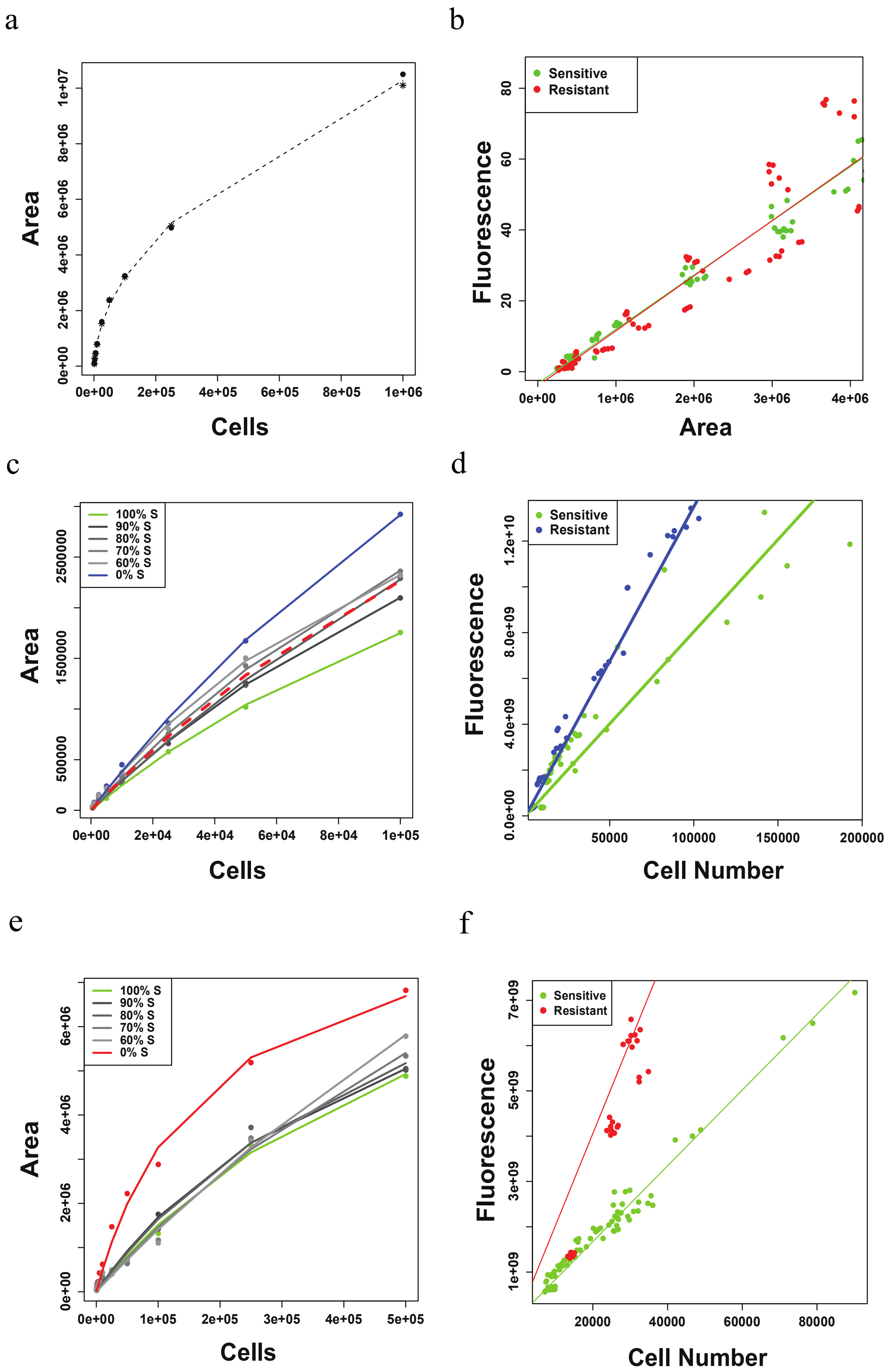
a
Area
Cells
b
Sensitive
Resistant
Fluorescence
Area
c
100% S
90% S
80% S
70% S
60% S
0% S
Area
Cells
d
Sensitive
Resistant
Fluorescence
Cell Number
e
100% S
90% S
80% S
70% S
60% S
0% S
Area
Cells
f
Sensitive
Resistant
Fluorescence
Cell Number

Supp. Figure 1. **Relationship of cell counts with area and fluorescence. a,** For CAMA-1 cells, the relationship of area to cell counts follows a nonlinear curve and is similar for sensitive and resistant cells (results not shown). **b,** Sensitive and resistant CAMA-1 cells also have similar relationships of area to fluorescence as described previously (Grolmusz et al, 2020). **c,** With LY2 cells, sensitive and resistant cells have different relationships of area to cell count, with cocultured cells falling in the middle. **d,** In addition, resistant LY2 cells show a higher per cell fluorescence. **e,** With MCF7 cells, sensitive and resistant cells have different relationships of area to cell count, with cocultured cells matching sensitive cell monocultures. **f,** Resistant MCF7 cells show a higher per cell fluorescence. Source data provided in Source Data file and csv files beginning suppfig1.

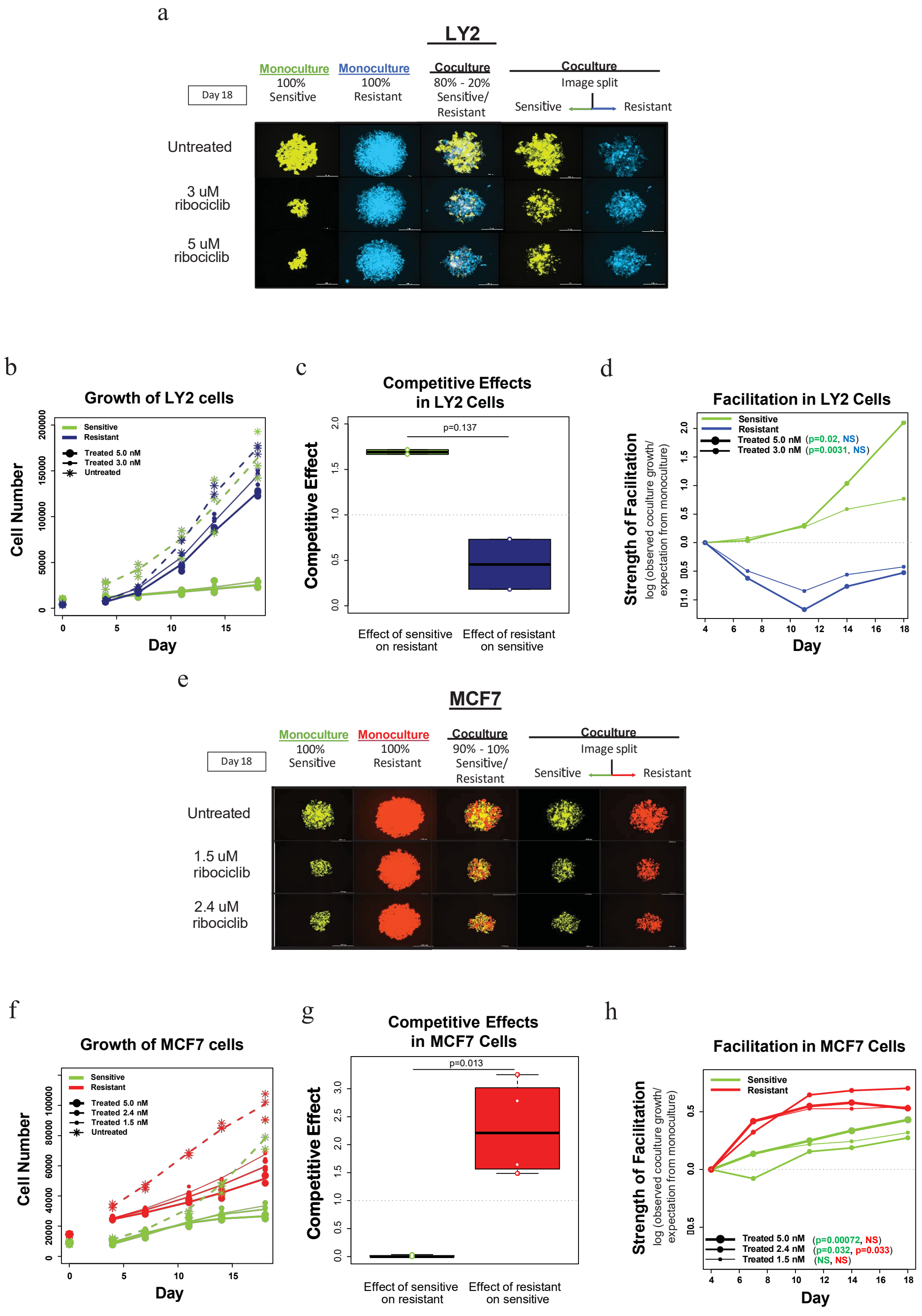
a
LY2
Monoculture
100%
Sensitive
Monoculture
100%
Resistant
Coculture
80% - 20%
Sensitive/
Resistant
Coculture
Image split
Sensitive
Resistant
Day 18
Untreated
3 uM
ribociclib
5 uM
ribociclib
b
Growth of LY2 cells
Sensitive
Resistant
Treated 5.0 nM
Treated 3.0 nM
Untreated
Cell Number
Day
c
Competitive Effects
in LY2 Cells
p=0.137
Competitive Effect
Effect of sensitive
on resistant
Effect of resistant
on sensitive
d
Facilitation in LY2 Cells
Sensitive
Resistant
Treated 5.0 nM (p=0.02, NS)
Treated 3.0 nM (p=0.0031, NS)
Strength of Facilitation
log (observed coculture growth/
expectation from monoculture)
Day
e
MCF7
Monoculture
100%
Sensitive
Monoculture
100%
Resistant
Coculture
90% - 10%
Sensitive/
Resistant
Coculture
Image split
Sensitive
Resistant
Day 18
Untreated
1.5 uM
ribociclib
2.4 uM
ribociclib
f
Growth of MCF7 cells
Sensitive
Resistant
Treated 5.0 nM
Treated 2.4 nM
Treated 1.5 nM
Untreated
Cell Number
Day
g
Competitive Effects
in MCF7 Cells
p=0.013
Competitive Effect
Effect of sensitive
on resistant
Effect of resistant
on sensitive
h
Facilitation in MCF7 Cells
Sensitive
Resistant
Treated 5.0 nM (p=0.00072, NS)
Treated 2.4 nM (p=0.032, p=0.033)
Treated 1.5 nM (NS, NS)
Strength of Facilitation
log (observed coculture growth/
expectation from monoculture)
Day

Supp. Figure 2. **Facilitation in other ER+ cell lines. a,** LY2 spheroids of different cell compositions cultured in untreated, 3uM ribociclib, and 5uM ribociclib treated medium for 18 days. Images taken at Day 18. **b,** Without ribociclib, resistant LY2 cells grew similarly to sensitive cells showing smaller growth reductions under treatment. **c,** Like CAMA-1, competitive effects in LY2 cells were highly asymmetric (sensitive cells reducing resistant cell growth by 50% more than resistant cells, and resistant cells had almost no competitive effect on sensitive cells). Due to the small sample size of n=2, the result only trends toward significance (p=0.137,t=4.472, difference in means=1.235, 95% confidence interval (-2.129,4.599)). **d,** Under treatment, sensitive LY2 cells grew significantly more in coculture than expected (n=3 for each treatment, mean values shown, facilitation measured as slope of the log of observed over expected cell number against day, at 3nM dose, p=0.0031, effect size=0.0612, SE=0.00692; At 5nM dose, p=0.02, effect size=0.1477, SE=0.0441). There was no significant facilitation effect on resistant cells. **e,** MCF7 spheroids of different cell compositions cultured in untreated, 1.5uM ribociclib, and 2.4uM ribociclib treated medium for 18 days. Images taken at Day 18. MCF7 resistant cells retained some response to ribociclib, however, had a greater resistance at an EC50 of 335.3M compared to the parental line's EC50 of 121.1M **f,** Without ribociclib, resistant MCF7 cells grew more quickly than sensitive cells, and showed similar growth reductions under treatment. **g,** Competitive effects in MCF7 cells were highly asymmetric though opposite of CAMA-1 (resistant cells reducing sensitive cell growth by 100% more than sensitive cells, and sensitive cells having almost no competitive effect on resistant cells) (n=4, p=0.013, t=5.29, difference in means=-2.282, 95% confidence interval (-3.654,-0.910)). **h,** Facilitatory increases in cocolutured sensitive and resistant MCF7 cells growth under treatment (tests as in panel d, no significant effects at 1.5nM, sensitive cells at 2.4nM dose, p=0.032, effect size=0.0235, SE=0.0062; resistant cells at 2.4nM dose, p=0.033, effect size=0.0511, SE=0.0136; sensitive cells at 5.0nM dose, p=0.00072, effect size=0.0303, SE=0.0021; resistant cells at 5.0nM dose, no significant effect). There was no significant difference in the strength of facilitation between sensitive and resistant MCF7 cells across ribociclib doses. Source data provided in Source Data file and csv files beginning suppfig2.

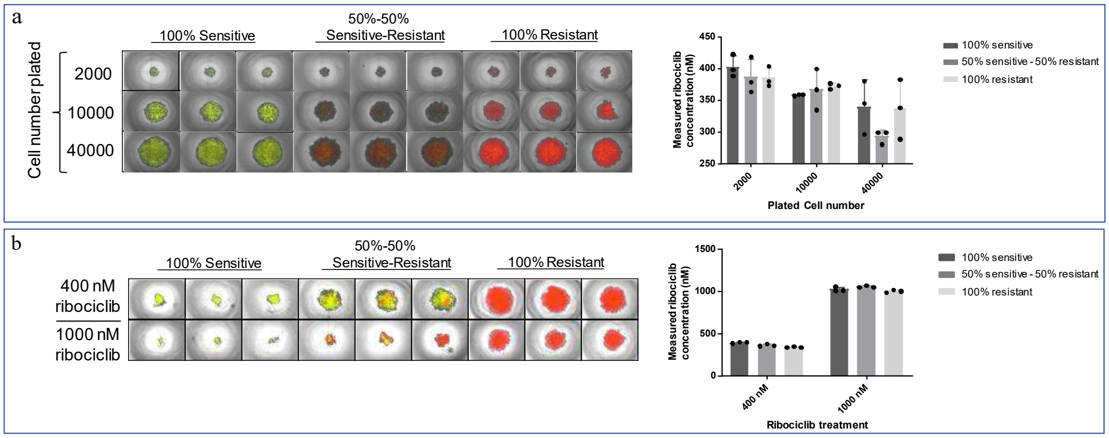

***Supp. Figure 3*. Analysis of ribociclib metabolism by sensitive and resistant spheroids. a,** Cells were plated at the indicated numbers (2000, 10000, 40000) and composition (100% sensitive, 50%-50% sensitive-resistant, and 100% resistant) in 3D spheroid format. Spheroids were subjected to 4 days continuous treatment with 400nM ribociclib to assess metabolism of drug by cells. Experiment was performed in triplicates for each condition; Ribociclib concentration measured by HPLC/MS for each population from media samples collected from experiment. Bar plots show mean and SD of post experiment ribociclib concentrations across populations with differing initial number of cells (colors). Points indicate replicates (n=3). **b,** 3D spheroids of different composition (100% sensitive, 50%-50% sensitive-resistant, and 100% resistant) were treated with continuous 400nM or 1000nM ribociclib to assess metabolism of drug by cells. Experiment was performed in triplicates for each condition; Ribociclib concentration measured by HPLC/MS for each population from media samples collected between days 18-21 of experiment. Bar plots show mean and SD of post experiment ribociclib concentrations across treated or untreated populations of resistant or sensitive cells (color). Points indicate replicates (n=3). Source data provided in Source Data file.

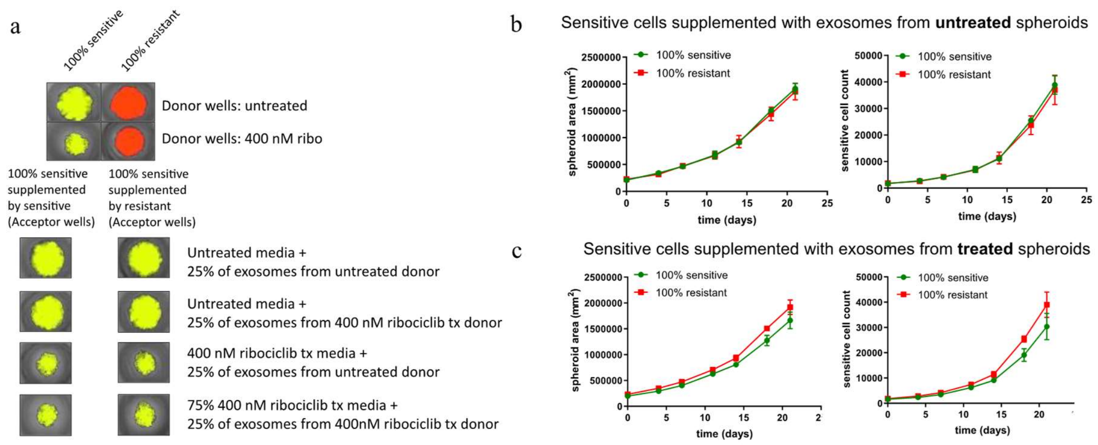

Supp. Figure 4. **Sensitive cells supplemented with isolated exosomes from conditioned media.** Comparing growth of drug treated spheroids supplemented with exosomes derived from media taken from spheroids of either 100% sensitive (fluorescently labeled with green) or 100% resistant (fluorescently labeled with red) cells. **a,** Labeled cells were plated with the indicated composition of sensitive and resistant cells in 3D spheroids under no treatment or 400nM ribociclib treatment to produce conditioned media (donor wells). 25 % of total exosomes isolated from conditioned media of donor wells were added to acceptor wells (100% sensitive spheroids) with or without ribociclib treatment. Sample size is n=3 for each cell line/condition. **b,** Normalized spheroid area and cell count of sensitive cells (from "acceptor wells") supplemented with exosomes from untreated conditioned media generated from either 100% sensitive or 100% resistant cells. Sample size is n=3 for each cell line. Data plotted as mean with SD. **c,** Normalized spheroid area and cell count of sensitive cells (from "acceptor wells") supplemented with exosomes from treated conditioned media generated from either 100% sensitive or 100% resistant cells. Sample size is n=3 for each cell line. Data plotted as mean with SD. Source data provided in Source Data file.

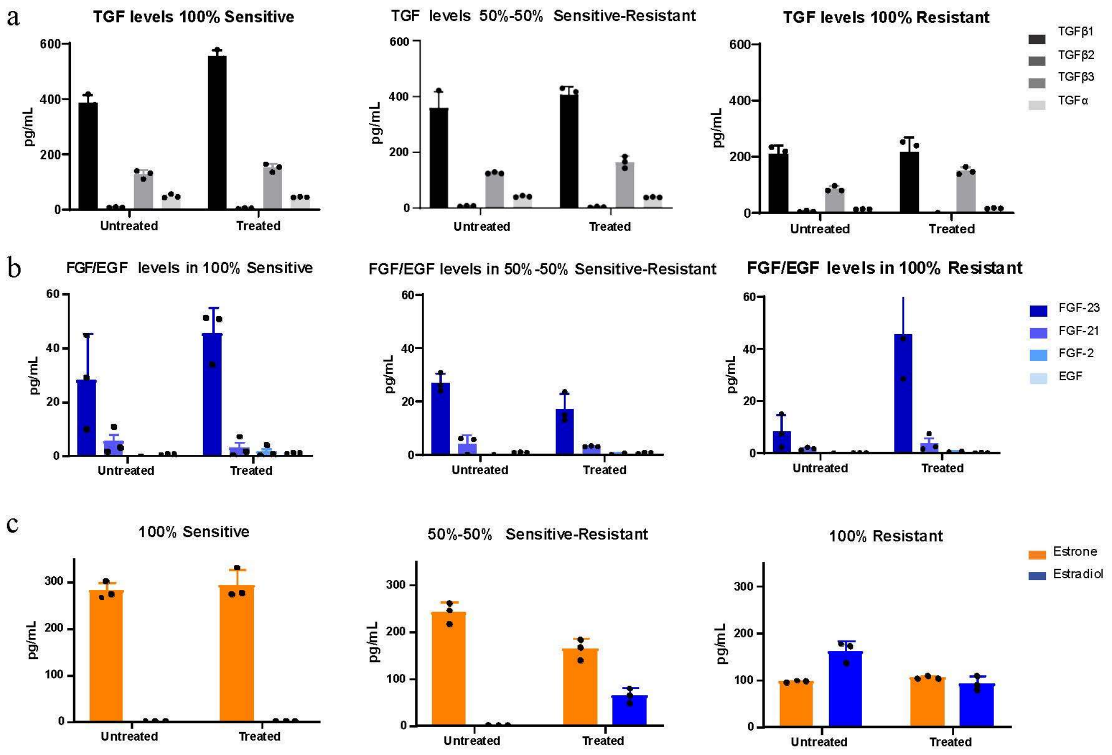

***Supp. Figure 5*. Underlying biological processes influencing facilitation in response to ribociclib treatment.** Media samples taken at day 21 from 3D spheroid with differing compositions (100% sensitive, 50% sensitive-50% resistant, 100% resistant). Barplots show mean with SD of signaling molecules in treated and untreated populations. Points show replicates (n=3). Samples used for **a**, multiplex cytokine analysis for TGF detection under untreated and ribociclib 200nM treated conditions **b**, multiplex cytokine analysis for FGF/EGF detection under untreated and 200nM ribociclib treated conditions **c**, liquid chromatography-tandem mass spectrometry (LC-MS/MS) assay for estradiol detection under untreated or ribociclib 200nM treated conditions. Source data provided in Source Data file.

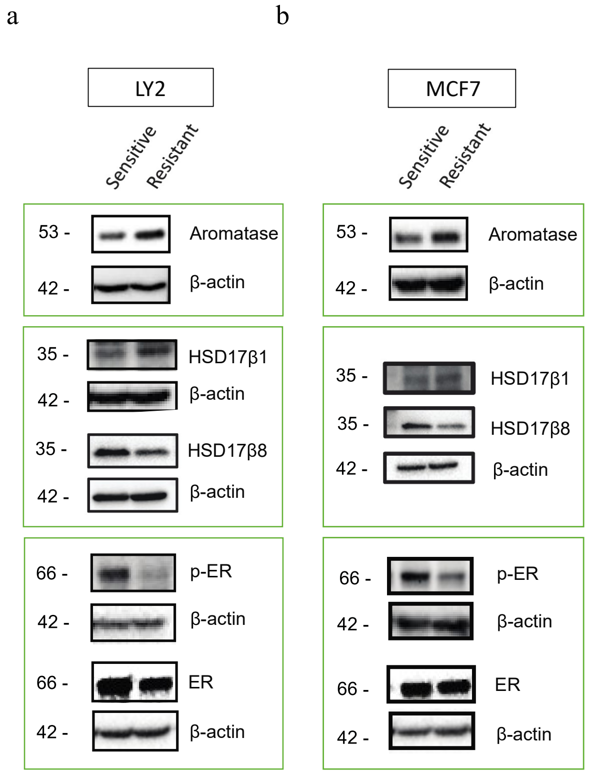

***Supp. Figure 6*. Protein levels of estradiol metabolism conversion enzymes.** Western blot detection of Aromatase, HSD17β1, HSD17β8, phosphorylated-ER, total ER, and β-actin in **a,** LY2 and **b,** MCF7 sensitive and resistant cells. Cells were cultured for 24 hours in complete media. The cell protein was separated by SDS-PAGE and transferred to a membrane that was then blotted with anti-aromatase, anti-HSD17β1, anti-HSD18β8, anti-phosphorylated-ER, anti-ER, or anti-b-actin. Western Blots were performed in triplicates with consistent findings. Uncropped Western blot images provided in Source Data file.

a

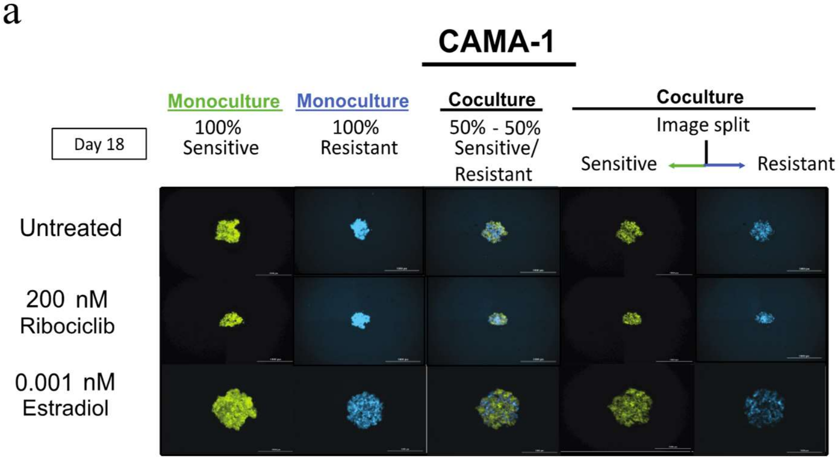

b

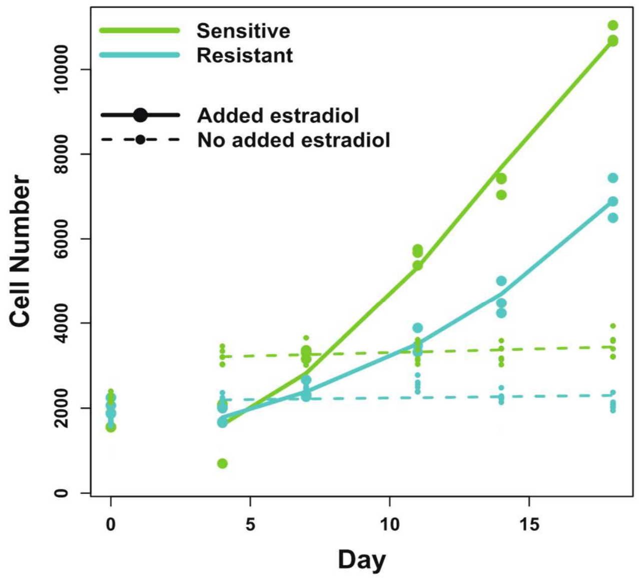

***Supp. Figure 7*. CAMA-1 sensitive and resistant mono and cocultured spheroids in charcoal-stripped FBS (estradiol free) media. a,** CAMA-1 spheroids of different cell compositions (100% sensitive - green, 50% sensitive- 50% resistant, 100% resistant - blue) cultured in untreated, 200 nM ribociclib, and 0.001 nM estradiol treated medium for 18 days. Images taken at Day 18. **b,** Growth curves of CAMA-1 untreated and estradiol treated (0.001 nM) sensitive and resistant cell populations. Source data provided in Source Data file and csv files beginning suppfig7.

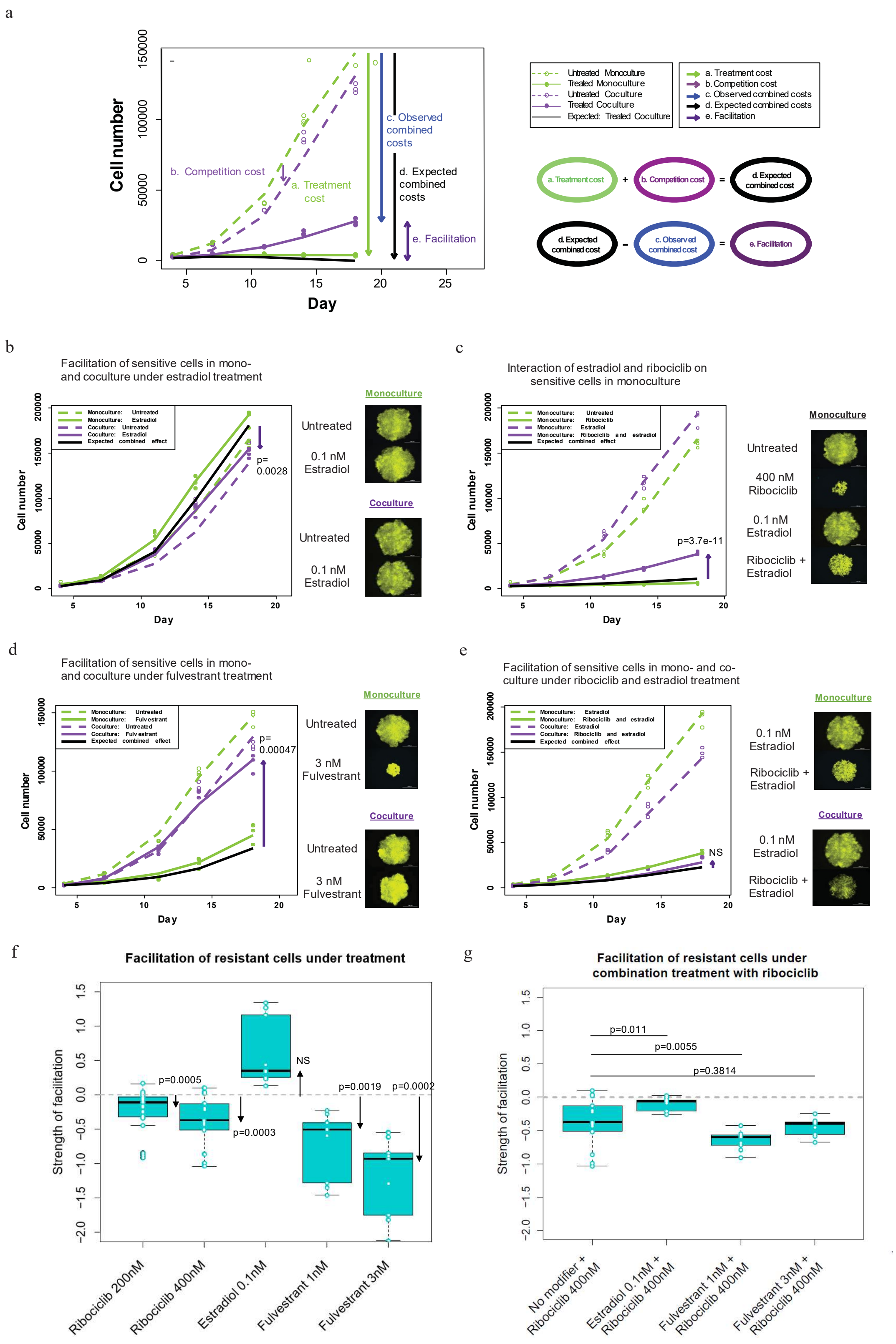
a
Cell number
Day
b. Competition cost
a. Treatment cost
c. Observed combined costs
d. Expected combined costs
e. Facilitation
Untreated Monoculture
Treated Monoculture
Untreated Coculture
Treated Coculture
Expected: Treated Coculture
a. Treatment cost
b. Competition cost
c. Observed combined costs
d. Expected combined costs
e. Facilitation
a. Treatment cost + b. Competition cost = d. Expected combined cost
d. Expected combined cost - c. Observed combined cost = e. Facilitation
b
Facilitation of sensitive cells in mono- and coculture under estradiol treatment
Monoculture: Untreated
Monoculture: Estradiol
Coculture: Untreated
Coculture: Estradiol
Expected combined effect
p= 0.0028
Monoculture
Untreated
0.1 nM Estradiol
Coculture
Untreated
0.1 nM Estradiol
c
Interaction of estradiol and ribociclib on sensitive cells in monoculture
Monoculture: Untreated
Monoculture: Ribociclib
Monoculture: Estradiol
Monoculture: Ribociclib and estradiol
Expected combined effect
p=3.7e-11
Monoculture
Untreated
400 nM Ribociclib
0.1 nM Estradiol
Ribociclib + Estradiol
d
Facilitation of sensitive cells in mono- and coculture under fulvestrant treatment
Monoculture: Untreated
Monoculture: Fulvestrant
Coculture: Untreated
Coculture: Fulvestrant
Expected combined effect
p= 0.00047
Monoculture
Untreated
3 nM Fulvestrant
Coculture
Untreated
3 nM Fulvestrant
e
Facilitation of sensitive cells in mono- and co-culture under ribociclib and estradiol treatment
Monoculture: Estradiol
Monoculture: Ribociclib and estradiol
Coculture: Estradiol
Coculture: Ribociclib and estradiol
Expected combined effect
NS
Monoculture
0.1 nM Estradiol
Ribociclib + Estradiol
Coculture
0.1 nM Estradiol
Ribociclib + Estradiol
f
Facilitation of resistant cells under treatment
Strength of facilitation
p=0.0005
p=0.0003
NS
p=0.0019
p=0.0002
Ribociclib 200nM
Ribociclib 400nM
Estradiol 0.1nM
Fulvestrant 1nM
Fulvestrant 3nM
g
Facilitation of resistant cells under combination treatment with ribociclib
Strength of facilitation
p=0.011
p=0.0055
p=0.3814
No modifier + Ribociclib 400nM
Estradiol 0.1nM + Ribociclib 400nM
Fulvestrant 1nM + Ribociclib 400nM
Fulvestrant 3nM + Ribociclib 400nM

Supp. Figure 8. **Facilitation in sensitive and resistant cells under single agent or combination treatment of ribociclib, estradiol, and fulvestrant. a,** Method for calculating deviation from null model. Green arrow indicates the growth reduction due to treatment, purple arrow the growth reduction due to competition, and blue arrow the observed combined effect. Black curve indicates the expected growth if the two reductions operated independently, with the difference from observed showing facilitation. This notation is used in panels b-e, with the statistical framework as in Figure 1f. **b**, Sensitive cells received no facilitation from resistant cells in the presence of estradiol, growing less than predicted (n=3 for each treatment, the log ratio of observed cells to predicted cells decreased with time in a linear model, slope of the effect of day=-0.0125, SE=0.00341, p=0.0028). **c,** Sensitive cell growth in with ribociclib was reduced by a smaller amount with estradiol (n=3 for each treatment, the log ratio of observed cells to predicted cells increased with time in a linear model, slope of the effect of day=0.0949, SE=0.00473, p=3.7e-11,). **d,** Sensitive cells received strong facilitation from resistant cells under fulvestrant (n=3 for each treatment, the log ratio of observed cells to predicted cells decreased with time in a linear model, slope of the effect of day=0.0849, SE=0.0183, p=0.00047). **e,** Facilitation of sensitive cell growth by resistant cells under ribociclib was cancelled by the addition of estradiol, with no significant difference of observed and expected growth (p=0.128). **f,** Facilitation of resistant cells under monotherapy. When treated with ribociclib or fulvestrant, resistant cells in coculture with sensitive cells were significantly negatively facilitated due to increased competition from sensitive cells (n=9 for each case, Ribociclib 200nM, p=0.0005, t=-3.967, mean=-0.214, 95% C.I. (-0.324,-0.103); Ribociclib 400nM, p=0.0003, t=-4.500, mean=-0.361, 95% C.I. (-0.530,-0.192); Fulvestrant 1nM, p=0.0019, t=-4.522, mean=-0.734, 95% C.I (-1.109,-0.360); Fulvestrant 3nM, p=0.0002, t=-6.329, mean=-1.204, 95% C.I. (-1.643,-0.765)). With 0.1nM estradiol treatment, resistant cells showed significant facilitation (p=0.0059, two-tailed t-test, t=-3.713, mean=0.609, 95% C.I. (0.231,0.988)). Box plots with center line=median, box=25th-75th percentile, and whiskers=5th-95th percentile, outliers=open circles, all statistical comparisons with t-tests. **g**, Facilitation of resistant cells under combination treatment (n=18 without modifier, n=9 for each case with modifier). Two-sided ANOVA compared with ribociclib monotherapy, combination treatment with estradiol significantly increased facilitation (p=0.011,t=1.953,effect size=0.2590 with SE=0.0980). Facilitation effects were negative at both doses of fulvestrant (Fulvestrant 1nM, p=0.0055, t=-2.93, mean= -0.2874, SE=0.098; Fulvestrant 3nM, p=0.3814, t=-0.88, mean=-0.0867). Source data provided in Source Data file and csv files beginning suppfig8.

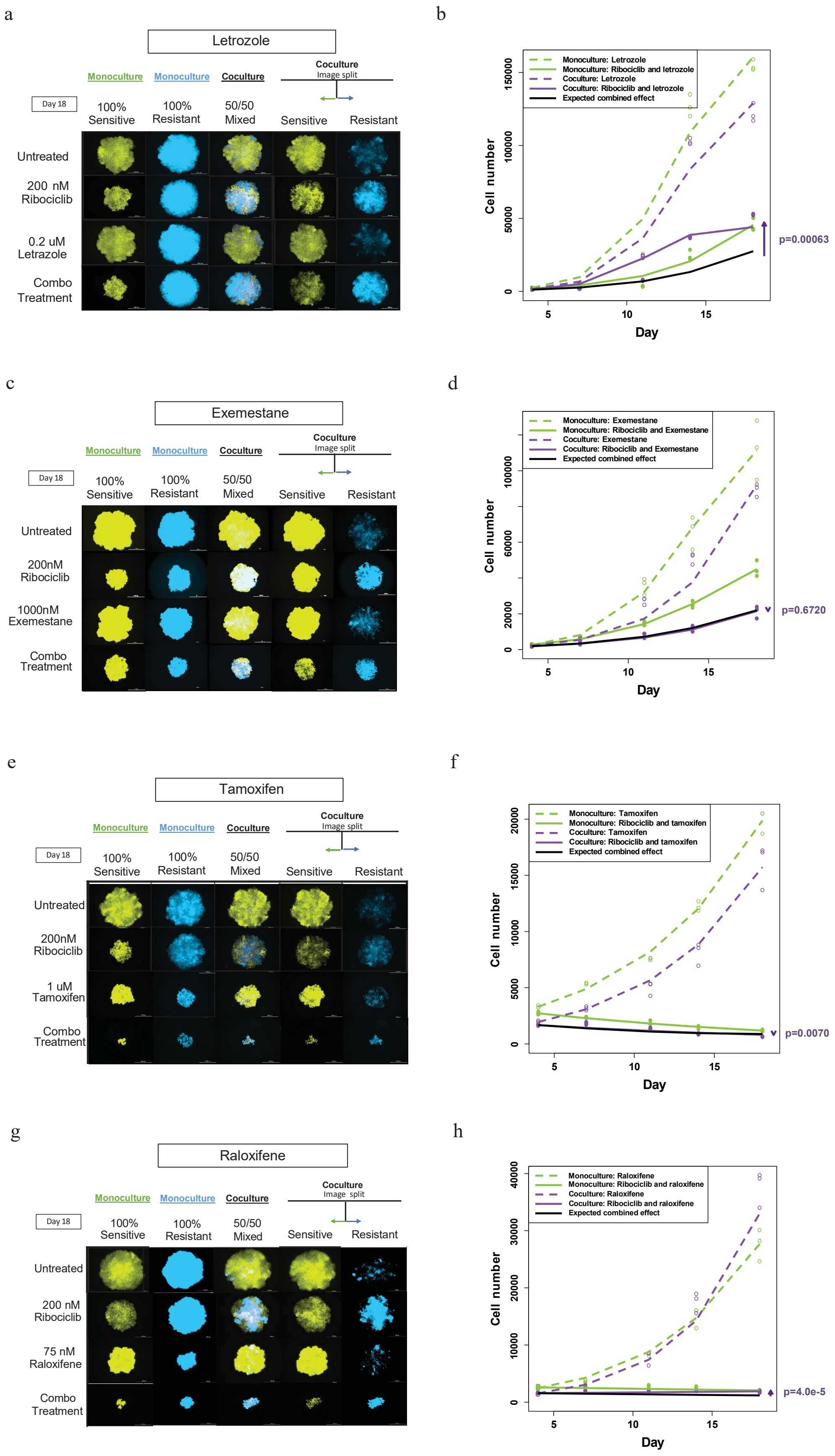
a
Letrozole
Monoculture
Monoculture
Coculture
Coculture
Image split
Day 18
100% Sensitive
100% Resistant
50/50 Mixed
Sensitive
Resistant
Untreated
200 nM Ribociclib
0.2 uM Letrazole
Combo Treatment
b
Monoculture: Letrozole
Monoculture: Ribociclib and letrozole
Coculture: Letrozole
Coculture: Ribociclib and letrozole
Expected combined effect
Cell number
Day
p=0.00063
c
Exemestane
Monoculture
Monoculture
Coculture
Coculture
Image split
Day 18
100% Sensitive
100% Resistant
50/50 Mixed
Sensitive
Resistant
Untreated
200nM Ribociclib
1000nM Exemestane
Combo Treatment
d
Monoculture: Exemestane
Monoculture: Ribociclib and Exemestane
Coculture: Exemestane
Coculture: Ribociclib and Exemestane
Expected combined effect
Cell number
Day
p=0.6720
e
Tamoxifen
Monoculture
Monoculture
Coculture
Coculture
Image split
Day 18
100% Sensitive
100% Resistant
50/50 Mixed
Sensitive
Resistant
Untreated
200nM Ribociclib
1 uM Tamoxifen
Combo Treatment
f
Monoculture: Tamoxifen
Monoculture: Ribociclib and tamoxifen
Coculture: Tamoxifen
Coculture: Ribociclib and tamoxifen
Expected combined effect
Cell number
Day
p=0.0070
g
Raloxifene
Monoculture
Monoculture
Coculture
Coculture
Image split
Day 18
100% Sensitive
100% Resistant
50/50 Mixed
Sensitive
Resistant
Untreated
200 nM Ribociclib
75 nM Raloxifene
Combo Treatment
h
Monoculture: Raloxifene
Monoculture: Ribociclib and raloxifene
Coculture: Raloxifene
Coculture: Ribociclib and raloxifene
Expected combined effect
Cell number
Day
p=4.0e-5

***Supp. Figure 9*. Modifications of facilitation and spheroid growth by other aromatase and endocrine inhibitors.** CAMA-1 spheroids of different cell compositions (100% sensitive - green, 50% sensitive- 50% resistant, 100% resistant - blue) cultured under different endocrine therapy treatments for 18 days; images taken on day 18. **a,** Spheroids cultured in untreated, 200nM ribociclib, 0.2uM letrozole, or combination 200nM ribociclib with 0.2uM letrozole treated medium **b,** Facilitation under letrozole and ribociclib treatments in terms of log growth relative to expected, averaged over all replicates, for CAMA-1 cells, n=3 for each treatment ; Letrozole in combination with ribociclib reduced but did not cancel facilitation (p=0.00063 for interaction of cell type with day using a linear model of the log of observed over expected cell number, effect size=0.0720, SE=0.0161). **c,** Spheroids cultured in untreated, 200nM ribociclib, 1000nM exemestane, or combination 200nM ribociclib with 1000nM exemestane treated medium **d**, Facilitation under exemestane and ribociclib treatments in terms of growth relative to expected averaged over all replicates for CAMA-1 cells, n=3 for each treatment; Exemestane in combination with ribociclib cancelled facilitation (p=0.6720 for interaction of cell type with day using a linear model of the log of observed over expected cell number). **e,** Spheroids cultured in untreated, 200nM ribociclib, 1uM tamoxifen, or combination 200nM ribociclib with 1uM tamoxifen treated medium **f,** Facilitation under tamoxifen and ribociclib treatments in terms of growth relative to expected averaged over all replicates for CAMA-1 cells, n=3 for each treatment; Tamoxifen in combination with ribociclib reversed facilitation (p=0.0070 for interaction of cell type with day using a linear model of the log of observed over expected cell number, effect size=-0.0285, standard error=0.0089). **g,** Spheroids cultured in untreated, 200nM ribociclib, 75nM raloxifene, or combination 200nM ribociclib with 75nM raloxifene treated medium **h,** Facilitation under raloxifene and ribociclib treatments in terms of growth relative to expected averaged over all replicates for CAMA-1cells, n=3 for each treatment; Raloxifene in combination with ribociclib reduced but did not cancel facilitation (p=4.0e-5 for interaction of cell type with day using a linear model of the log of observed over expected cell number, effect size=0.0306, standard error=0.0050). Source data provided in Source Data file and csv files beginning suppfig9.

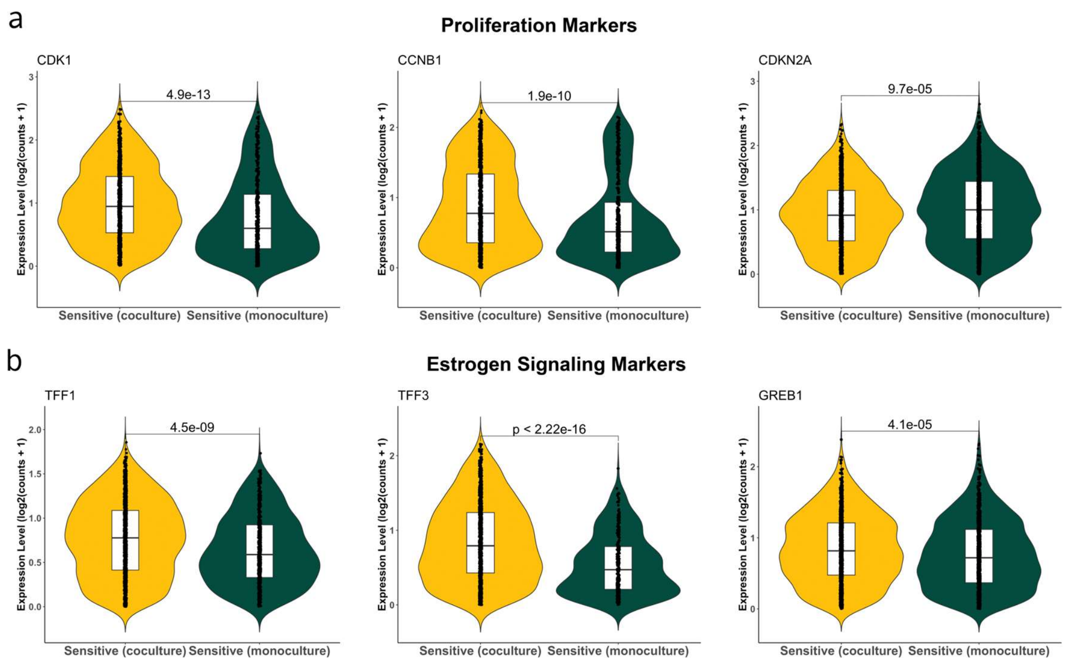

***Supp. Figure 10*. Single cell RNAseq reveals differences in estrogen and proliferation signaling markers between sensitive and resistant cells.** Violin plots showing normalized gene expression levels of **a,** proliferation markers CDK1, CCNB1, and CDKN2A and **b,** estrogen signaling makers, TFF1, TFF3, and GREB1. Expression was measured in ribociclib sensitive coculture (yellow) and sensitive monoculture (dark green) cell populations using scRNA-seq. Sensitive (coculture) N = 2059, Sensitive (monoculture) N = 2258. Cells with zero expression were removed before plotting and statistical analyses. Lower and upper hinges in the boxplots indicate 25th and 75th percentiles while middle indicates 50th percentile (median). The whiskers extend over 1.5× interquartile range. Boxplots contrast expression between mono and cocultured sensitive cells and p-values indicate significant expression differences between groups identified by a non-parametric (two-sided) Wilcox rank-sum test. Source data provided in Source Data file.

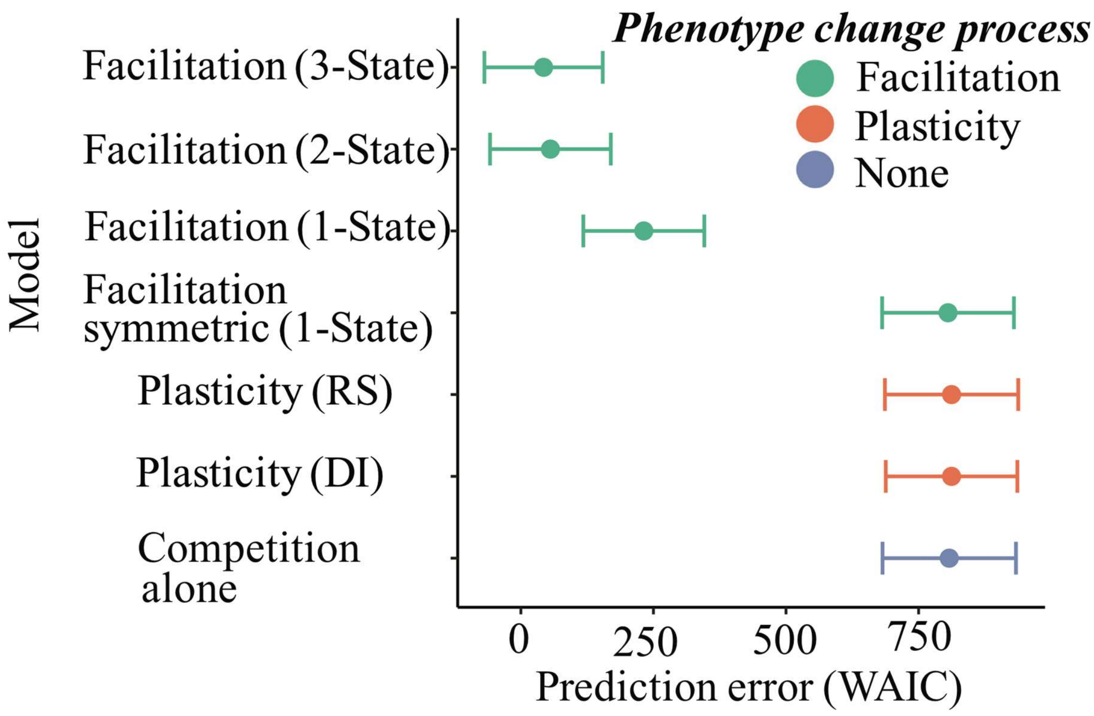

Supp. Figure 11. **Model comparison between alternative model hypotheses describing the mechanisms of cell-cell interactions impacting spheroid growth dynamics.** Comparison of the ability of facilitation models (green) to describe the resistant and sensitive CAMA1 spheroid growth data across drug doses, relative to models of phenotypic plasticity (red) or competition alone (blue). Low prediction error indicates that the model more accurately explains the spheroid growth data (n=504 spheroid measurements across 7 days, 8 doses and 2 cell types), whilst avoiding overfitting the data by penalizing the model likelihood based on model complexity (using WAIC: widely applicable information criterion). Error bars indicate the confidence interval around the models estimated goodness of fit.

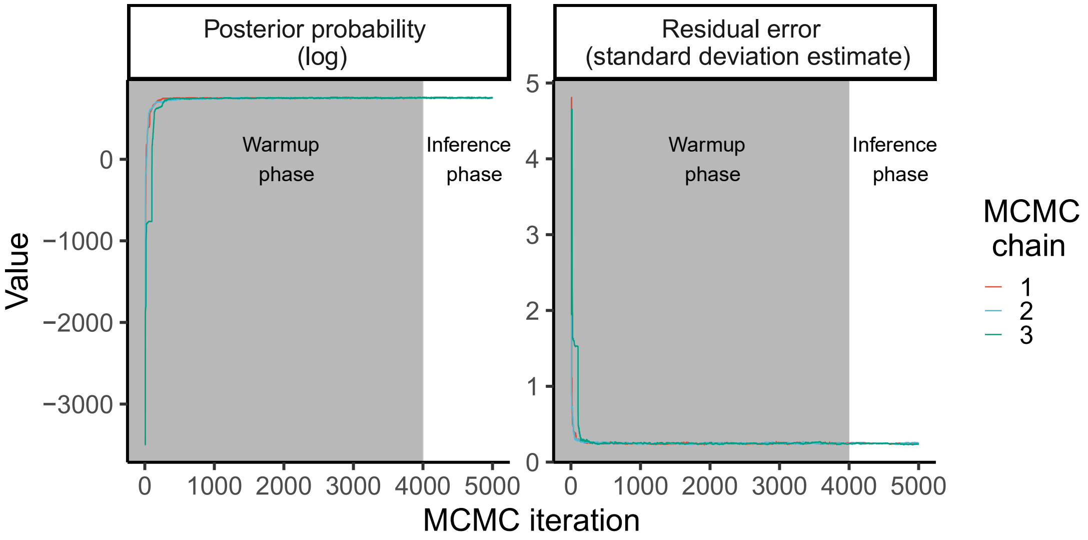

***Supplementary Figure 12*. Bayesian inference converged.** Estradiol mediated facilitation model inference performance during three independent MCMC chains. Bayesian inference converged early in the warmup period (shaded region; n=4000 Hamiltonian Monte Carlo iterations) of inference and the models posterior probability (left) and residual error (right) was stable throughout the inference phase (unshaded region; n=1000 Hamiltonian Monte Carlo iterations). MCMC samples from the warmup phase were discarded and the iterations from the inference phase were used to determine posterior parameter distributions.

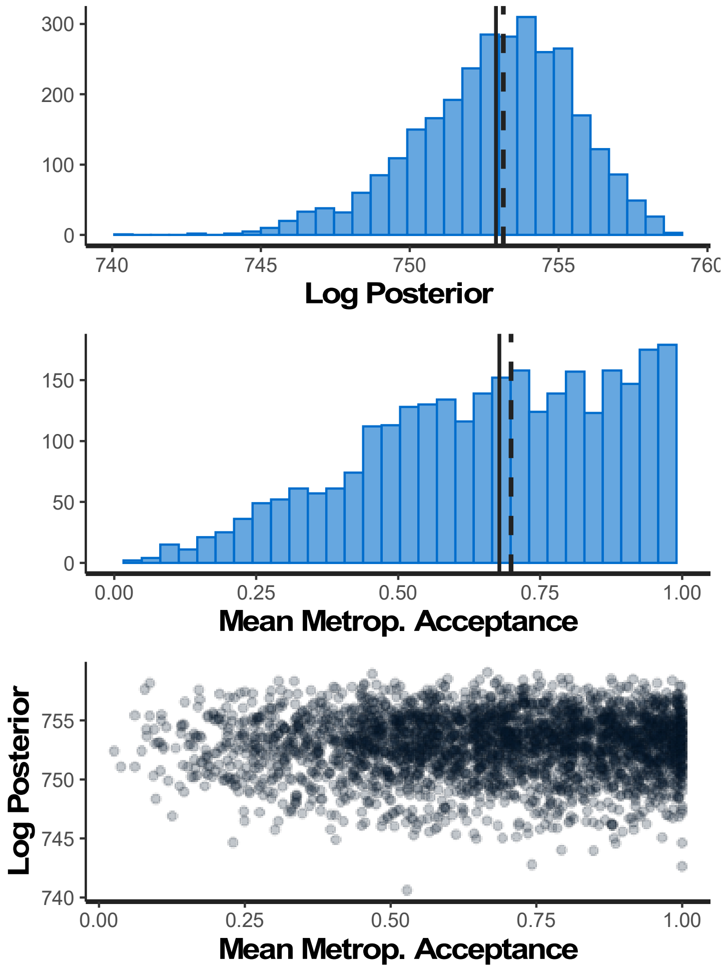

Supplementary Figure 13. **Diagnostic plots of MCMC inference.** Diagnostics show stability of fitted Estradiol mediated facilitation posterior probability throughout the MCMC chains (top row; solid line=mean log posterior probability across warmup and inference phase, dashed line= mean log posterior probability in the inference phase), achievement of a metropolis acceptance rate close to the desired rate of 0.7 (middle row; solid line= target acceptance rate,) and a lack of correlation between these metric (bottom row)

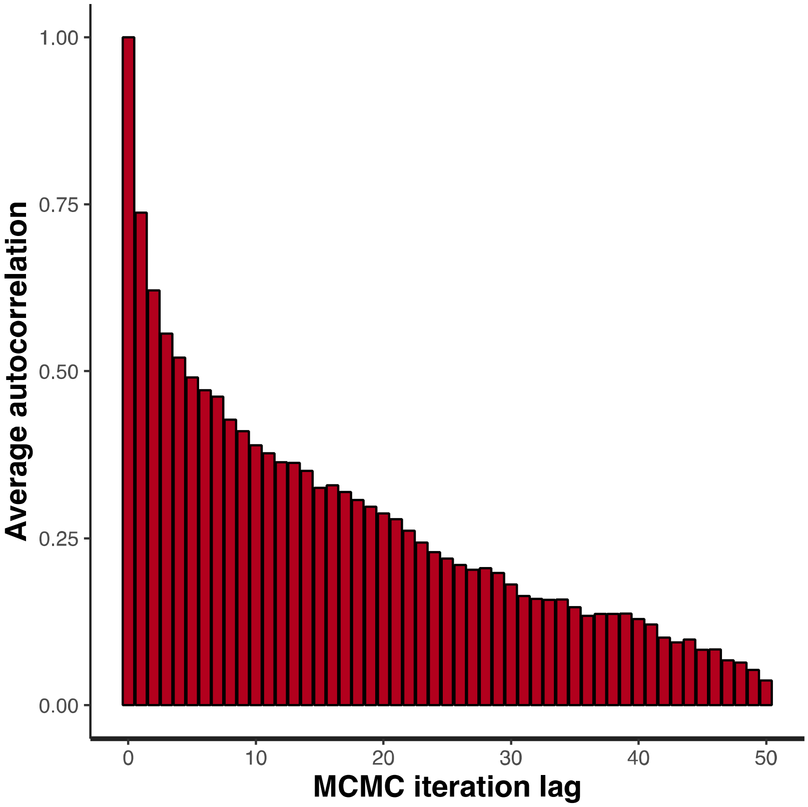

Supplementary Figure 14. **Bayesian MCMC inference autocorrelation diagnostic plot.** Rapid decline of autocorrelation in fitted model probability indicates effective exploration of parameter space.

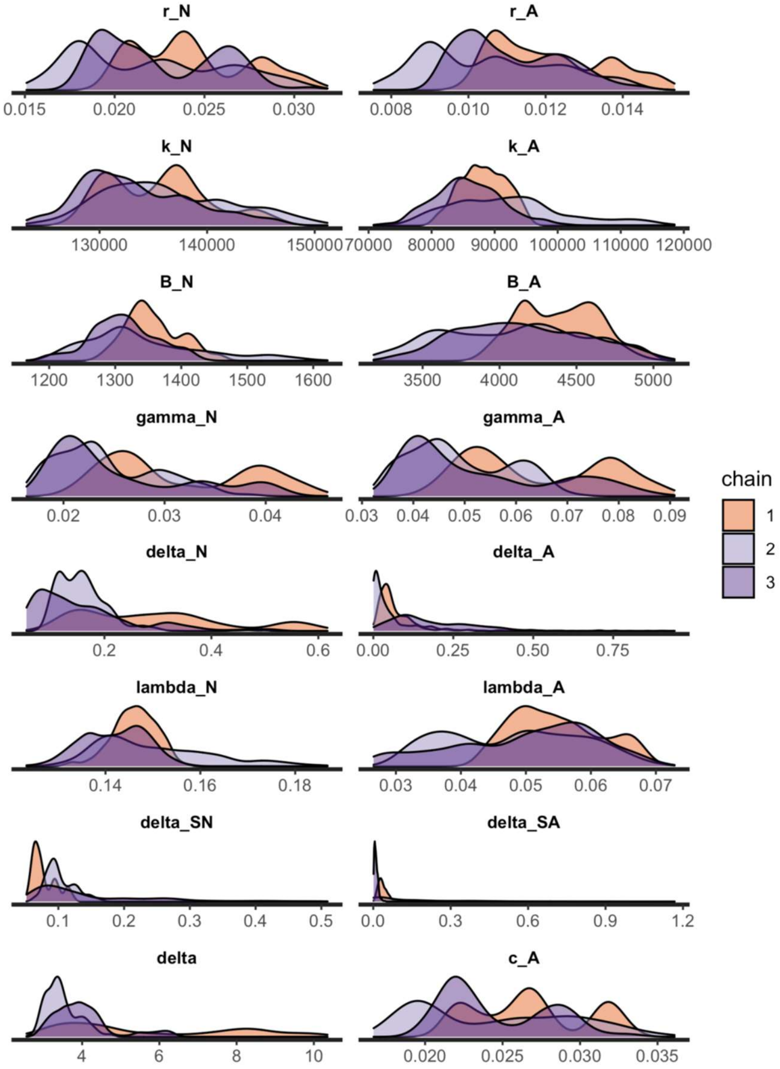

Supplementary Figure 15 Estradiol mediated facilitation model parameter estimates obtained from the inference phase of three independent MCMC chains had a convergent posterior distribution across all parameter. See methods for description of each parameters (briefly, r_R/r_S= resistant and sensitive cell baseline growth rate, α_R/α_S = resistant and sensitive cell competition coefficient = 1/carrying capacity (K_R/K_S), k_R/k_S= resistant and sensitive cell half maximum drug concentration γ_R/γ_S = resistant and sensitive cell facilitation factor production, φ_R/φ_S = resistant and sensitive cell senescence rate, λ_R/λ_S = resistant and sensitive cell baseline quiescence , δ_R/δ_S = resistant and sensitive cell death rate, δ_E = facilitation factor degradation rate, c = resistant and sensitive cell limitation of facilitation factor uptake and binding).

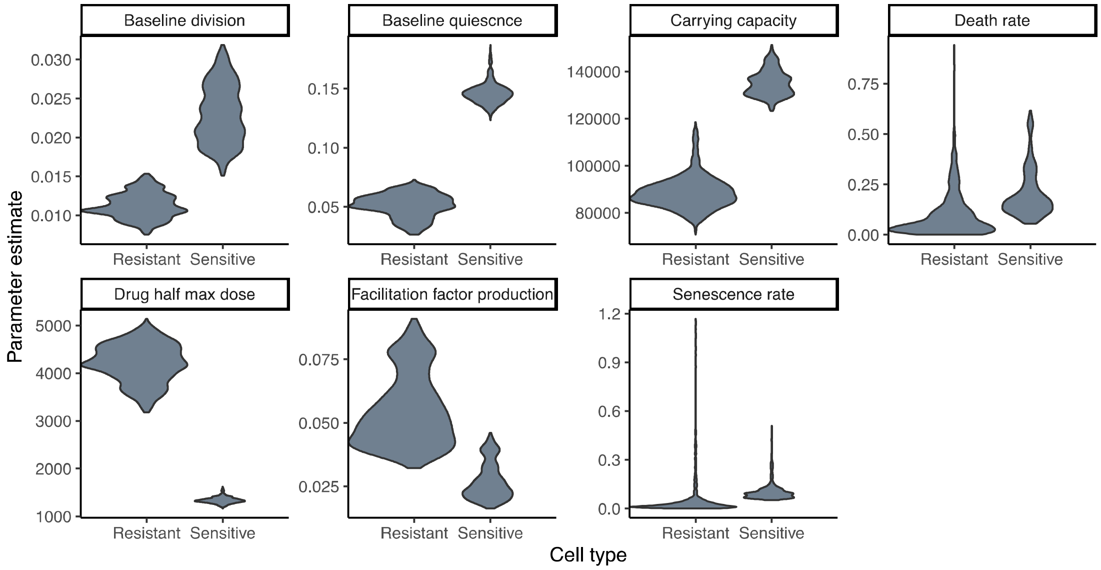

Supplementary Figure 16 Comparison of estradiol mediated facilitation model parameter estimate of biological processes (panels) in sensitive and resistant cells (x axis). Violin plots show the posterior distribution of parameter estimates (y axis), showing the range of uncertainty in the parameter for each cell type. Each biological parameter that was allowed to vary between resistant and sensitive cells (panels) had equal prior distributions for the cell types' rates. Therefore differences in parameter estimates between cell types are informed by the likelihood of the spheroid growth trajectory data.

Uncropped and unprocessed scans of blots from Supplemental Figure 6.

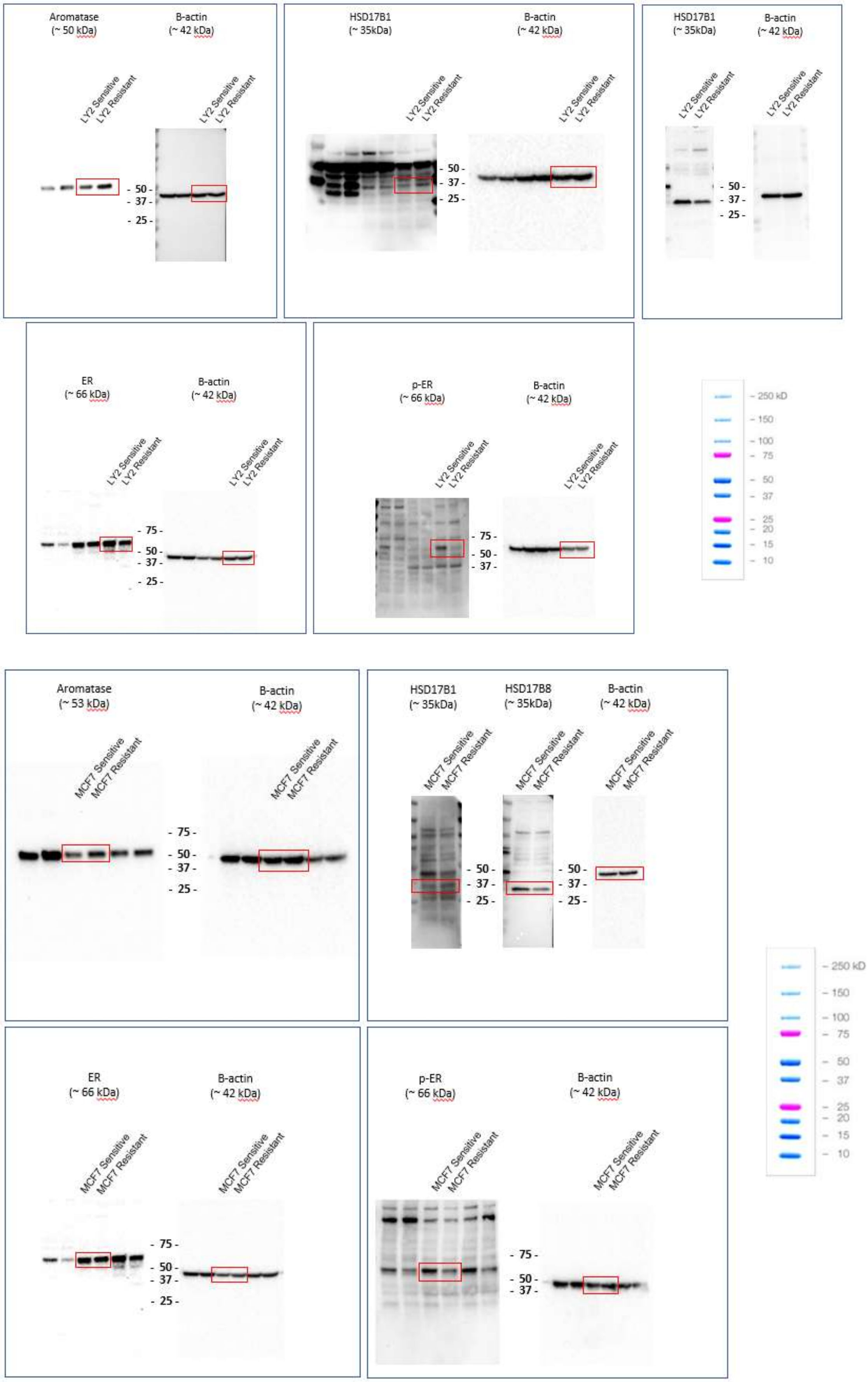

Aromatase
(~ 50 kDa)
B-actin
(~ 42 kDa)
LY2 Sensitive
LY2 Resistant
- 50 -
- 37 -
- 25 -
HSD17B1
(~ 35kDa)
B-actin
(~ 42 kDa)
HSD17B1
(~ 35kDa)
B-actin
(~ 42 kDa)
ER
(~ 66 kDa)
B-actin
(~ 42 kDa)
- 75 -
p-ER
(~ 66 kDa)
B-actin
(~ 42 kDa)
- 250 kD
- 150
- 100
- 75
- 50
- 37
- 25
- 20
- 15
- 10
Aromatase
(~ 53 kDa)
B-actin
(~ 42 kDa)
MCF7 Sensitive
MCF7 Resistant
HSD17B1
(~ 35kDa)
HSD17B8
(~ 35kDa)
B-actin
(~ 42 kDa)
ER
(~ 66 kDa)
B-actin
(~ 42 kDa)
p-ER
(~ 66 kDa)
B-actin
(~ 42 kDa)

REFERENCES:

1. De Roos AM, Schellekens T, Van Kooten T, Van De Wolfshaar K, Claessen D, Persson L. Simplifying a physiologically structured population model to a stage-structured biomass model. Theor Popul Biol. 2008;73(1):47-62. Epub 2007/11/17. doi: 10.1016/j.tpb.2007.09.004. PubMed PMID: 18006030.
2. R Core Team. R: A language and environment for statistical computing (2016).
3. Carpenter, B. et al. Stan: A Probabilistic Programming Language. J. Stat. Softw. (2016).
4. Vehtari, Aki, Andrew Gelman, and Jonah Gabry. "Practical Bayesian model evaluation using leave-one-out cross-validation and WAIC." *Statistics and computing* 27.5 (2017): 1413-1432.
