# Supplementary material for: Cell facilitation promotes growth and survival under drug pressure in breast cancer
Source: Nat Commun. 2023 Jun 29;14:3851. doi: 10.1038/s41467-023-39242-6 (PMC10310817; doi:10.1038/s41467-023-39242-6)
Supplement: Supplementary file 2 — Reporting Summary [file 41467_2023_39242_MOESM2_ESM.pdf]

nature portfolio

Corresponding author(s): Rena Emond, Jason I. Griffiths, Vince Kornél Grolmusz, Aritro Nath, Jinfeng Chen, Eric F. Medina, Rachel S. Sousa, Timothy Synold, Frederick R. Adler, Andrea H. Bild

Last updated by author(s): May 5, 2023

# Reporting Summary

Nature Portfolio wishes to improve the reproducibility of the work that we publish. This form provides structure for consistency and transparency in reporting. For further information on Nature Portfolio policies, see our Editorial Policies and the Editorial Policy Checklist.

## Statistics

For all statistical analyses, confirm that the following items are present in the figure legend, table legend, main text, or Methods section.

| n/a | Confirmed | |
|---|---|---|
| ☐ | ☒ | The exact sample size (*n*) for each experimental group/condition, given as a discrete number and unit of measurement |
| ☐ | ☒ | A statement on whether measurements were taken from distinct samples or whether the same sample was measured repeatedly |
| ☐ | ☒ | The statistical test(s) used AND whether they are one- or two-sided<br>*Only common tests should be described solely by name; describe more complex techniques in the Methods section.* |
| ☐ | ☒ | A description of all covariates tested |
| ☐ | ☒ | A description of any assumptions or corrections, such as tests of normality and adjustment for multiple comparisons |
| ☐ | ☒ | A full description of the statistical parameters including central tendency (e.g. means) or other basic estimates (e.g. regression coefficient) AND variation (e.g. standard deviation) or associated estimates of uncertainty (e.g. confidence intervals) |
| ☐ | ☒ | For null hypothesis testing, the test statistic (e.g. *F*, *t*, *r*) with confidence intervals, effect sizes, degrees of freedom and *P* value noted<br>*Give P values as exact values whenever suitable.* |
| ☐ | ☒ | For Bayesian analysis, information on the choice of priors and Markov chain Monte Carlo settings |
| ☐ | ☒ | For hierarchical and complex designs, identification of the appropriate level for tests and full reporting of outcomes |
| ☒ | ☐ | Estimates of effect sizes (e.g. Cohen's *d*, Pearson's *r*), indicating how they were calculated |

*Our web collection on statistics for biologists contains articles on many of the points above.*

## Software and code

Policy information about availability of computer code

| | |
|---|---|
| Data collection | For 3D organoid experiments, data was collected by imaging organoid populations using Cytation 5 Imager (Biotek Instruments) to capture 3D structures and fluorescence intensity, and raw data processing was performed using Gen5 (v.3.05 and v.3.10 software - Biotek Instruments). For LC-MS/MS experiments, Analyst software (version 1.5.1) was used for data acquisition. For the HPLC/MS experiments, MassLynx (version 4.1) software was used for data acquisition. Western blots were visualized with BioRad Image Lab software (v.6.1). For bulk sequencing experiments - reads were aligned with STAR aligner (v.2.7.0) to human reference genome 19 (hg19) and transcripts quantified using RSEM (v1.3.1). For single cell sequencing, CellRanger (v3.0.2) was used to align reads using reference genome (GRChg37) \. The custom code used in analyses, mathematical modeling and to produce figures 1-5 are available on GitHub at: https://github.com/U54Bioinformatics/FacilitationRibociclibBreast |
| Data analysis | For 3D organoid experiments, image analysis was performed using Gen5 v.3.05 and v.3.10 software (Biotek Instruments) and GraphPad Prism v.7.02 software. For LC-MS/MS experiments, Analyst software v.1.5.1 was used for data processing. For the multiplex cytokine analysis experiments Flexmap 3D Luminex system (Luminex corp.) and Bio-Plex Manager 6.2 software were used. For the HPLC/MS experiments, MassLynx v.4.1 software was used for data processing and analysis. For bulk RNA sequencing data analyses, Library normalization was performed with the R package edgeR (v3.40.2) and differential expression analyses was performed with the stats package (v4.2.1). Dot plots were generated with the ggplot2 (v3.4.1). For single-cell RNA-sequencing data analyses, the R packages Seuart (v3.1.1.9023), gsva (v1.46.0), and msigdbr (v7.5.1), and the python package scrublet (v0.2). R programming language (v4.2.1) was used for bioinformatics analyses. The custom code used in analyses, mathematical modeling and to produce figures 1-5 are available on GitHub at: https://github.com/U54Bioinformatics/FacilitationRibociclibBreast |

For manuscripts utilizing custom algorithms or software that are central to the research but not yet described in published literature, software must be made available to editors and reviewers. We strongly encourage code deposition in a community repository (e.g. GitHub). See the Nature Portfolio guidelines for submitting code & software for further information.

## Data

Policy information about availability of data

All manuscripts must include a data availability statement. This statement should provide the following information, where applicable:

- Accession codes, unique identifiers, or web links for publicly available datasets
- A description of any restrictions on data availability
- For clinical datasets or third party data, please ensure that the statement adheres to our policy

For bulk sequencing analysis, reads were aligned to human reference genome 19 (hg19). For the single cell sequencing analysis, sequence reads were processed using reference genome (GRChg37). RNA data is available under accession codes GSE143944 (CAMA-1 bulk RNA-Seq) and GSE193278 (CAMA-1 scRNA-Seq). All original experimental datasets produced and used are available in provided source file with manuscript submission and made available at https://github.com/U54Bioinformatics/FacilitationRibociclibBreast.

## Human research participants

Policy information about studies involving human research participants and Sex and Gender in Research.

| | |
|---|---|
| Reporting on sex and gender | N/A |
| Population characteristics | N/A |
| Recruitment | N/A |
| Ethics oversight | N/A |

Note that full information on the approval of the study protocol must also be provided in the manuscript.

# Field-specific reporting

Please select the one below that is the best fit for your research. If you are not sure, read the appropriate sections before making your selection.

☒ Life sciences ☐ Behavioural & social sciences ☐ Ecological, evolutionary & environmental sciences

For a reference copy of the document with all sections, see nature.com/documents/nr-reporting-summary-flat.pdf

# Life sciences study design

All studies must disclose on these points even when the disclosure is negative.

| | |
|---|---|
| Sample size | 3D organoid experiments were performed on available and established ribociclib sensitive and resistant cell lines by plating 2,000 cells per well (for CAMA-1 and LY2) and 5,000 cells per well (for MCF7). Plating densities were optimized to allow for experimental time frame (18-21 days) while considering cell growth and spacial capacity of round bottom wells for 96 well plate. The observed consistency of spheroid growth trajectories across replicate experiments was used to determine sufficiency of sample size. For Western blots, cell numbers were plated to obtain ~80% confluency for collection and sample preparation. For sequencing experiments, no variables were predetermined prior to this study for measurement and power calculations. |
| Data exclusions | Some data points were excluded from analysis due to quality control related to image acquisition failure and sample loss during culture. |
| Replication | 3D organoid experiments, RNA seq (bulk) experiments, and Western blots were performed in triplicates and successful. For RNAseq (single-cell), LC-MS/MS, and HPLC/MS experiments, due to time and cost restraints, experiments were performed once across technical replicates. |
| Randomization | Randomization was not implemented as cell line experiments involved cell lines receiving similar treatment conditions and controls, thus not requiring bias-reducing measures. |
| Blinding | Blinding was not required as human subject data was not used and cell line experiments did not require bias-reducing measures. Although investigators were not blinded to allocation during experiments, outcomes were qualitatively assessed using automated imaging protocols and software. |

# Reporting for specific materials, systems and methods

We require information from authors about some types of materials, experimental systems and methods used in many studies. Here, indicate whether each material, system or method listed is relevant to your study. If you are not sure if a list item applies to your research, read the appropriate section before selecting a response.

## Materials & experimental systems

| n/a | Involved in the study |
|---|---|
| ☐ | ☒ Antibodies |
| ☐ | ☒ Eukaryotic cell lines |
| ☒ | ☐ Palaeontology and archaeology |
| ☒ | ☐ Animals and other organisms |
| ☒ | ☐ Clinical data |
| ☒ | ☐ Dual use research of concern |

## Methods

| n/a | Involved in the study |
|---|---|
| ☒ | ☐ ChIP-seq |
| ☒ | ☐ Flow cytometry |
| ☒ | ☐ MRI-based neuroimaging |

# Antibodies

Antibodies used

- anti-aromatase monoclonal antibody (Invitrogen, rabbit, Cat #: MA5-32628, Lot #: WH3361115, clone # JM10-68)
- anti-HSD17β1 monoclonal antibody (Abnova, mouse, Cat #: H00003292-M03A, lot #: 10215-2E5, clone #: 2E5; R&D systems, mouse, Cat #: MAB7178, Lot #: CHXF0116081, clone # 860020)
- anti-HSD17β8 polyclonal antibody (Proteintech, Cat #: 16752-1-AP, Lot #: 0098114)
- anti-ER monoclonal antibody (Cell Signaling, rabbit, Cat #: 8644S, Lot #: 10, Clone #: D8H8)
- anti-phospho-ER monoclonal antibody (Cell Signaling, mouse, Cat #: 2511S, Lot #:4, Clone #: 16J4)
- anti-β-actin monoclonal antibody (Santa Cruz Biotechnology, mouse, Cat #: sc-47778, Lot #: G0121, Clone #: C-4 )
- anti-rabbit peroxidase-linked secondary antibody (GE Healthcare, Cat #: NA9341ML, Lot #: 9814746)
- anti-mouse peroxidase-linked secondary antibody (GE Healthcare, Cat #: NXA9311ML, Lot #: 17246087)

Validation

- Anti-aromatase monoclonal antibody tested by ThermoFisher for Western Blot at dilution 1:500-1:2000. The expression systems are developed by cloning in the specific antibody DNA sequences from immunoreactive rabbits
- Anti-HSD17β1 monoclonal antibody tested by Abnova for Western Blot at 1 ~ 5 ug/ml. Mouse monoclonal antibody raised against a partial recombinant HSD17B1. Antibody use referenced in publications (Kumata et al, 2018; McNamara et al, 2016; Inoue et al, 2011; Chanplakorn et al, 2010).
- Anti-HSD17β1 monoclonal antibody tested by R&D Systems for Western Blot with 0.25ug/mL of mAb. Source: monoclonal Mouse IgG2B Clone # 860020.
- anti-HSD17β8 polyclonal antibody tested by Proteintech for Western Blot at dilution 1:500-1:2000 with positive WB detected in HeLa cells, mouse liver tissue, mouse small intestine tissue, human testis tissue.
- anti-ER tested by Cell Signaling for Western Blot at dilution 1:1000 with validation statement: "This antibody has been validated using SimpleChIP® Enzymatic Chromatin IP Kits. The CUT&RUN dilution was determined using CUT&RUN Assay Kit #86652". Antibody use currently referenced in 192 publications (e.g. Chioma et al, 2023; Chen et al, 2022; Li et. al, 2022).
- anti-phospho-ER tested by Cell Signaling for Western Blot at dilution 1:1000. Monoclonal antibody is produced by immunizing animals with a synthetic phosphopeptide corresponding to residues surrounding Ser118 of human ER α. Antibody use currently referenced in 84 publications (e.g. Chen et al, 2022; Wang et al, 2021; DiGiacomo et al, 2021)
- anti-β-actin monoclonal antibody. Antibody use currently referenced in 13338 publications (e.g. Teicher et al, 1990; Zou et al, 2021)
- anti-rabbit peroxidase-linked secondary antibody tested by GE Healthcare for Western Blot with dilution 1:5000 with statement: "For every batch of enzyme-linked antibody that is produced, the antibody titre is determined on an ELISA...every batch is also QC tested in a Western blotting syste,. This is performed using Hyband ECL membrane containing serially diluted Beta-galactosidase protein and immundetected with primary antibody anti-beta-galactosidase and secondary antibody NA934, anti-rabbit HRP. Blots are detected using ECL and ECL Plus detection systems."
- anti-mouse peroxidase-linked secondary antibody tested by GE Healthcare for Western Blot with dilution 1:2000-1:5000 with statement: "For every batch of enzyme-linked antibody that is produced the antibody titre is determined in an ELISA. The substrate used for the peroxidase is 2,2′-azinobis[3-ethylbenzothiazoline sulphonate, diammonium salt], ABTSTM. Every batch is also QC tested in a Western blotting system. This is performed using HybondTM ECLTM membrane containing tubulin protein and immunodetected with: primary antibody, monoclonal anti-tubulin; and secondary antibody NA 931, antimouse IgG, HRP. Blots are detected using ECL and ECL PlusTM detection systems."

# Eukaryotic cell lines

Policy information about cell lines and Sex and Gender in Research

Cell line source(s)

MCF7, LY2, and CAMA-1 from ATCC.

Authentication

Authentication of cell lines was accomplished at ATCC using STR profiling against ATCC STR database.

Mycoplasma contamination

All cell lines tested negative for mycoplasma contamination.

Commonly misidentified lines
(See ICLAC register)

MCF7 is listed in the ICLAC register. This cell line was used in the study to provide evidence of scientific findings in more than 1 cell line (3 cell lines tested for facilitation cell-cell interaction) and was chosen from a specific/limited group of resistant cell lines previously generated and fluorescently labeled in our lab. This cell line was a supporting cell line shown in supplementary figures but not main figures.
